# Exploring the 3,5-Dibromo-4,6-dimethoxychalcones and Their Flavone Derivatives as Dual α-Glucosidase and α-Amylase Inhibitors with Antioxidant and Anticancer Potential

**DOI:** 10.3390/antiox13101255

**Published:** 2024-10-17

**Authors:** Jackson K. Nkoana, Malose J. Mphahlele, Garland K. More, Yee Siew Choong

**Affiliations:** 1 Department of Chemistry, College of Science, Engineering and Technology, University of South Africa, Private Bag X06, Florida 1710, South Africa; nkoankj@unisa.ac.za; 2 College of Agriculture and Environmental Sciences, University of South Africa, Private Bag X06, Florida 1710, South Africa; moregk@unisa.ac.za; 3 Institute for Research in Molecular Medicine (INFORMM), Universiti Sains Malaysia, George Town 11800, Penang, Malaysia; yeesiew@usm.my

**Keywords:** 3,5-dibromo-4,6-dimethoxychalcones, 6,8-dibromo-5,7-dimethoxyflavones, carbohydrate-hydrolyzing enzymes, antioxidant activity, cytotoxicity, molecular docking

## Abstract

The rising levels of type 2 diabetes mellitus (T2DM) and the poor medical effects of the commercially available antidiabetic drugs necessitate the development of potent analogs to treat this multifactorial metabolic disorder. It has been demonstrated that targeting two or more biochemical targets associated with the onset and progression of diabetes along with oxidative stress and/or cancer could be a significant strategy for treating complications related to this metabolic disorder. The 3,5-dibromo-4,6-dimethoxychalcones (**2a**–**f**) and the corresponding flavone derivatives (**3a**–**f**) were synthesized and characterized using spectroscopic (NMR, HR-MS and FT-IR) techniques. The inhibitory effect of both series of compounds against α-glucosidase and α-amylase was evaluated in vitro through enzymatic assays. Selected compounds were also evaluated for potential to activate or inhibit superoxide dismutase. Compound **3c** was selected as a representative model for the flavone series and evaluated spectrophotometrically for potential to coordinate Cu(II) and/or Zn(II) ions implicated in the metal-catalyzed free radical generation. A plausible mechanism for metal-chelation of the test compounds is presented. Furthermore, the most active compounds from each series against the test carbohydrate-hydrolyzing enzymes were selected and evaluated for their antigrowth effect on the human breast (MCF-7) and lung (A549) cancer cell lines and for cytotoxicity against the African Green Monkey kidney (Vero) cell line. The parent chalcone **2a** and flavone derivatives **3a**, **3c** and **3e** exhibited relatively high inhibitory activity against the MCF-7 cells with IC_50_ values of 4.12 ± 0.55, 8.50 ± 0.82, 5.10 ± 0.61 and 6.96 ± 0.66 μM, respectively. The chalcones **2a** and **2c** exhibited significant cytotoxicity against the A549 cells with IC_50_ values of 7.40 ± 0.67 and 9.68 ± 0.80 μM, respectively. Only flavone **3c** exhibited relatively strong and comparable cytotoxicity against the MCF-7 and A549 cell lines with IC_50_ values of 6.96 ± 0.66 and 6.42 ± 0.79 μM, respectively. Both series of compounds exhibited strong activity against the MCF-7 and A549 cell lines compared to the analogous quercetin (IC_50_ = 35.40 ± 1.78 and 35.38 ± 1.78 μM, respectively) though moderate compared to nintedanib (IC_50_ = 0.53 ± 0.11 and 0.74 ± 0.15 μM, respectively). The test compounds generally exhibited reduced cytotoxicity against the Vero cells compared to this anticancer drug. Molecular docking revealed strong alignment of the test compounds with the enzyme backbone to engage in hydrogen bonding interaction/s and hydrophobic contacts with the residues in the active sites of α-glucosidase and α-amylase. The test compounds possess favorable drug-likeness properties, supporting their potential as therapeutic candidates against T2DM.

## 1. Introduction

Type 2 diabetes mellitus (T2DM) is a chronic metabolic disease that is characterized by persistent elevated blood sugar (hyperglycemia). The latter is the result of insufficient production of insulin by pancreatic beta (β)-cells or development of insulin resistance mainly due to obesity and the release of inflammatory cytokines [1]. These effects lead to disturbances in carbohydrate, lipid and protein metabolism. One of the strategies to control or reduce postprandial hyperglycemia (PPHG) is to inhibit the activity of α-glucosidase and/or α-amylase in the digestive tract [1]. α-Amylase facilitates the hydrolysis of large starch molecules into absorbable components that are, in turn, metabolized by α-glucosidase into glucose for intestinal absorption. Inhibition of α-amylase activity generally suppresses carbohydrate digestion by decreasing the bioavailability of oligosaccharides and absorbable sugars, resulting in reduced blood sugar levels. α-Amylase inhibitors serve as dietary starch blockers by limiting the metabolism and absorption of starch in the gastrointestinal diet [2]. α-Glucosidase inhibitors, on the other hand, retard the release of glucose from dietary complex carbohydrates and thus delay glucose absorption, in turn suppressing PPHG [3]. Selective and strong inhibition of α-amylase would result in a considerable amount of undigested complex carbohydrates and starch accumulating in the gastrointestinal tract [2,4,5,6]. The accumulated biomaterial will eventually be degraded by bacteria in the colon, resulting in stomach distention, flatulence, diarrhea and abdominal pain. Although strong anti-α-glucosidase activity is desirable, selective α-glucosidase inhibition may also result in gastrointestinal side effects due to the localized action of this enzyme in the intestine [4]. Better efficacy and significantly reduced side effects require antihyperglycemic agents with dual anti-α-glucosidase and anti-α-amylase activity, preferably exhibiting a moderate α-amylase inhibitory effect [4,5]. Previous studies have revealed that hyperglycemia also triggers auto-oxidative glycosylation activating protein kinase C, leading to the generation of the reactive oxygen species (ROS) and reactive nitrogen species (RNS) [7]. This process results in oxidative stress, which is also implicated in the early stages and progression of T2DM due to the development of insulin resistance caused by the disruption of the pro-oxidant/antioxidant balance [7,8,9]. Substantial evidence from the scientific and clinical studies links impaired glucose tolerance with cancer [10]. Furthermore, an inhibition of α-glucosidase has been found to have positive effects in the treatment of different types of cancers [11]. Metformin, a blood glucose-lowering drug prescribed to T2DM patients, for example, has been found to lower the risk of estrogen-positive breast cancer in females [12]. It is envisaged that targeting two or more biochemical targets linked to the onset and progression of T2DM along with oxidative stress and/or cancer could be a significant strategy for the treatment of this multifactorial metabolic disorder.

**Figure 1:**
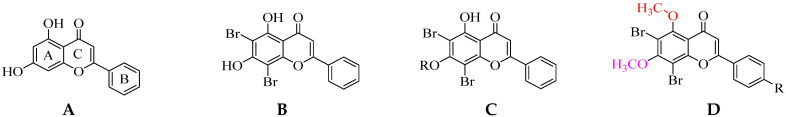
Chemical structures of chrysin **A**, bromo derivatives (**B** & **C**) and the target compounds **D**.

The chalcones [13] and flavones [14] have demonstrated beneficial effects against a wide range of biochemical targets in in vitro and in vivo studies. Several studies highlight these flavonoids as potential antidiabetic agents because of their strong α-glucosidase inhibition and moderate α-amylase inhibition, which make them suitable candidates for their development as antihyperglycemic drugs with reduced or no adverse side effects [4,5]. Chrysin (5,7-dihydroxyflavone) **A**, shown in Figure 1, is an example of a naturally occurring flavonoid with antidiabetic, anti-obesity, antioxidant, anti-inflammatory and anticancer properties as well as neuroprotective and hepatoprotective effects [15]. However, the pharmaceutical application of this flavone derivative is limited by its reduced solubility and oral bioavailability due to the hydroxyl groups, which are also prone to rapid intestinal or hepatic conjugation via glucuronidation or sulfation [16,17]. As a result, structural manipulation and derivatization of the chrysin scaffold continue to be undertaken with the aim of addressing these hurdles to increase its absorption, and in turn, improve its in vivo therapeutic efficacy [18,19]. Methoxylation of the flavone scaffold has been found to improve the aqueous solubility of the ligand and to enhance the membrane-penetrating properties, leading to facilitated absorption and increased oral bioavailability compared to the corresponding parent compounds [18,19,20,21,22]. Kumar et al. previously observed that substitution of the hydroxyl group at position-7 of the flavone scaffold with a methoxy group promotes anti-α-glucosidase activity [23]. The structure activity relationship (SAR) study of 6,8-dibromochrysin **B** and the 7-alkyloxy derivatives **C** (R = ethyl, butyl or hexyl) against α-glucosidase, on the other hand, revealed that the former exhibited increased activity in vitro compared to chrysin [16]. However, the presence of an O-alkyl group at the 7-position of chrysin or its 6,8-dibromo-substituted derivative **C** resulted in significantly reduced inhibition of α-glucosidase compared to the parent compound. Hitherto, the analogous 8-iodo-5,7-dimethoxychrysin derivatives were found to exhibit the strongest antigrowth activity against the human gastric adenocarcinoma (SGC-7901) cell line [24]. We envisage the observed significantly reduced anti-α-glucosidase activity of compounds **C** to be due to the bulky 7-alkoxy group.

It has previously been observed that a 5-methoxy group on the flavonoid scaffold results in resistance to oxidative metabolism in vivo by preventing the formation of glucuronic acid or sulfate conjugates [19]. The 7-methoxy group on the chromone framework is reported to be prone to oxidative metabolism by cytochrome P450 enzymes in vivo to afford phenolic metabolites with enhanced biological activity [25,26]. The glucuronide and sulfate conjugates of the 7-methoxylated flavones may provide a pool from which active hydroxyflavones can be released in vivo, which makes methoxyflavonoids suitable prodrugs. Prodrug design is a widely used molecular modification strategy to optimize the physicochemical and pharmacological properties of drugs, resulting in improved solubility and pharmacokinetic features as well as decreased toxicity [27]. The presence of polar substituents on the fused benzo (A) ring of the flavone imparts unique molecular characteristics, including polarity, stability and binding properties, among others. Polar substituents on this ring have been found to enable the dual anti-α-glucosidase and anti-α-amylase agents to engage in hydrogen bonding and/or hydrophobic interactions with residues in the catalytic site of these enzymes [5]. In this regard, the methoxy group can enhance the ligand’s target binding, physicochemical and ADME (absorption, distribution, metabolism and excretion) properties [28]. We considered the antihyperglycemic properties of methoxyflavonoids and their potential to serve as prodrugs to afford bioactive phenolic metabolites in vivo, and decided to synthesize the 6,8-dibromochrysin derivatives of the generalized structure **D**. The target 6,8-dibromoflavone scaffold is decorated with the 5- and 7-methoxy group as well as different substituents on the *para* position of the phenyl substituent (B-ring). Bromine atom has a σ-hole (region with a positive charge) and the lone pair electrons to engage in halogen bonding and hydrogen bonding interactions, respectively, to stabilize the drug–receptor complexes [29]. We made use of 3,5-dibromo-2-hydroxy-4,6-dimethoxyacetophenone as a precursor for the base-mediated Claisen–Schmidt aldol condensation with benzaldehyde derivatives followed by the cyclodehydration of the chalcone intermediates to afford the corresponding 6,8-dibromo-5,7-dimethoxyflavones. The chalcone intermediates and their flavone derivatives were, in turn, subjected to enzymatic assays in vitro for potential to inhibit α-glucosidase activity. The most active derivatives against α-glucosidase were also used for further evaluation against α-amylase. Their antioxidant potential, on the other hand, was evaluated spectrophotometrically using the nitric oxide (NO) radical scavenging assay. The derivatives with significant or strong NO radical scavenging activity were also screened through enzymatic assays in vitro for the potential to inhibit or activate superoxide dismutase (SOD). SOD enzymes prevent the toxic effects of free radicals by maintaining a balance between the production and scavenging of biological oxidants in the body [30]. Since flavones possessing only methoxy group/s are reported not to exhibit a direct radical scavenging effect like hydroxyflavones [31], a representative example was also evaluated spectrophotometrically for potential to chelate/coordinate Cu(II) and/or Zn(II) ions implicated in the metal-catalyzed free radical generation [32]. Due to the link between diabetes and cancer and the multifactorial origins of these human disorders, we screened selected compounds for their antiproliferative effect on the MCF-7 and A549 cancer cell lines. The compounds were also evaluated for cytotoxicity against the non-cancerous Vero cell line to establish their selectivity. A molecular docking (in silico) study was performed to predict the fit and orientation of the chalcones and flavones into the catalytic active sites of α-glucosidase and α-amylase to correlate the in silico and in vitro results. The pharmacokinetic (ADMET: absorption, distribution, metabolism, excretion and toxicity) properties have also been evaluated to predict their drug-likeness.

## 2. Materials and Methods

### 2.1. Materials and Instrumentation

The chemicals and solvents used in this study were purchased from Merck (Pty) Ltd. (Modderfontein, Johannesburg, South Africa) and were used without undergoing any further purification. The melting point (mp.) values of the prepared compounds were recorded on a Stuart SMP5 melting point apparatus (Coleparmer, Stone, Staffordshire, UK) and were not corrected. The Nuclear Magnetic Resonance (^1^H NMR and ^13^C NMR) spectra were recorded as deuterated dimethyl sulfoxide ((CD_3_)_2_SO) solutions using an Agilent 500 MHz NMR spectrometer (Agilent Technologies, Oxford, UK) operating at 500 MHz and 125 MHz for ^1^H and ^13^C nuclei, respectively. The chemical shifts were quoted relative to the residual protonated solvent or to tetramethylsilane (δ = 0.00 ppm) used as an internal reference standard. The ^19^F NMR spectra were recorded at 470 MHz and the chemical shift values (ppm) were quoted relative to CCl_3_F used as an external reference standard. The HR-MS analysis was performed at the University of Stellenbosch CAF (Central Analytical Facility) on a Waters Cyclic select IMS QTOF mass spectrometer coupled to a Waters UPLC, ESI probe, ESI Positive, Cone Voltage 15 V (Waters, Milford, MA, USA). The metal complexation studies, on the other hand, were performed using a Shimadzu UV/Vis spectrometer, model: UV-1800 240V (Shimadzu Europa GmbH, Albert-Han Strasse 6-10, 47269 Duisburg, Germany) at wavelengths ranging from 200 to 600 nm. 3,5-Dibromo-2-hydroxy-3,6-dimethoxyacetophenone (**1**) was prepared as described in the literature [33].

### 2.2. A Typical Method for the Synthesis of Chalcone Derivatives ***2a***–***f***

A mixture of 3,5-dibromo-2-hydroxy-3,6-dimethoxyacetophenone (2.00 g, 5.65 mmol) and a benzaldehyde derivative (5.65 mmol) in ethanol (30 mL) was treated with a 20% aqueous solution of potassium hydroxide (10 mL). The mixture was stirred at room temperature (RT) for 24 h and then quenched with an ice-cold aqueous ammonium chloride solution. The precipitate was filtered on a sintered funnel, washed with cold water and recrystallized from a mixture of dimethyl formamide (DMF) and toluene. The following compounds were prepared in this manner.

(*E*)-1-(3,5-Dibromo-2-hydroxy-4,6-dimethoxyphenyl)-3-phenylprop-2-en-1-one (**2a**)

Yellow solid (1.70 g, 68%), mp. 106–108 °C; ν_max_ (ATR) 572, 680, 752, 832, 962, 1205, 1332, 1386, 1440, 1544, 1631, 2938 cm^−1^; ^1^H NMR (500 MHz): δ 3.83 (s, 3H, -OCH_3_), 3.83 (s, 3H, -OCH_3_), 7.24 (d, *J* = 16.0 Hz, 1H, H-α), 7.43 (m, 3H, H-4′ and H-3′,5′), 7.47 (d, *J* = 16.0 Hz, 1H, H-β), 7.73 (dd, *J* = 2.5 and 8.5 Hz, 2H, H-2′,6′), 10.76 (s, 1H, -OH); ^13^C NMR (125 MHz): δ 60.6, 62.4, 103.7, 104.0, 120.3, 127.5, 129.0, 129.2, 131.1, 134.4, 145.8, 153.9, 155.5, 156.7, 192.3; ESI-MS: calculated for C_17_H_14_Br_2_O_4_, *m*/*z* = 439.9259; found: 439.9181.

(*E*)-1-(3,5-Dibromo-2-hydroxy-4,6-dimethoxyphenyl)-3-(4-fluorophenyl)prop-2-en-1-one (**2b**)

Yellow solid (1.38 g, 53%), mp. 132–134 °C; ν_max_ (ATR) 504, 685, 826, 968, 1095, 1205, 1322, 1394, 1510, 1563, 1631, 2947 cm^−1^; ^1^H NMR (500 MHz): δ 3.69 (s, 3H, -OCH_3_), 3.82 (s, 3H, -OCH_3_), 7.20 (d, *J* = 16.0 Hz, 1H, H-α), 7.25 (dd, *J* = 2.5 and 8.5 Hz, 2H, H-3′,5′), 7.47 (d, *J* = 16.0 Hz, 1H, H-β), 7.83 (dd, *J* = 2.5 and 8.5 Hz, 2H, H-2′,6′), 10.69 (s, 1H, -OH); ^13^C NMR (125 MHz): δ 61.1, 62.9, 104.1, 104.5, 116.8 (d, ^2^*J*_C-F_ = 21.8 Hz), 121.0, 128.0, 131.6 (d, ^4^*J*_C-F_ = 2.9 Hz), 132 (d, ^3^*J*_C-F_ = 8.5 Hz), 145.1, 154.2, 155.9, 157.1, 164.3 (d, ^1^*J*_C-F_ = 248.4 Hz), 192.8; ESI-MS: calculated for C_17_H_13_Br_2_FO_4_, *m*/*z* = 459.9144; found: 459.9120.

(*E*)-1-(3,5-Dibromo-2-hydroxy-4,6-dimethoxyphenyl)-3-(4-chlorophenyl)prop-2-en-1-one (**2c**)

Yellow solid (1.67 g, 62%), mp. 150–152 °C; ν_max_ (ATR) 626, 702, 824, 956, 1089, 1202, 1347, 1326, 1385, 1544, 1620, 2936 cm^−1^; ^1^H NMR (500 MHz): δ 3.70 (s, 3H, -OCH_3_), 3.83 (s, 3H, -OCH_3_), 7.25 (d, *J* = 16.0 Hz, 1H, H-α), 7.47 (m, 3H, H-3′,5′ and H-β), 7.77 (d, *J* = 8.5 Hz, 2H, H-2′,6′), 10,72 (s, 1H, -OH); ^13^C NMR (125 MHz): δ 60.6, 62.4, 103.6, 104.0, 120.4, 128.3, 129.2, 129.3, 130.7, 133.4, 135.7, 144.3, 153.7, 155.4, 156.6, 192.3 ESI-MS: calculated for C_17_H_13_Br_2_ClO_4_, *m*/*z* = 473.8869; found: 473.8791.

(*E*)-1-(3,5-Dibromo-2-hydroxy-4,6-dimethoxyphenyl)-3-(4-methoxyphenyl)prop-2-en-1-one (**2d**)

Yellow solid (1.25 g, 47%), mp. 155–158 °C; ν_max_ (ATR) 544, 685, 832, 928, 1047, 1089, 1205, 1326, 1391, 1541, 1631, 2930 cm^−1^; ^1^H NMR (500 MHz): δ 3.71 (s. 3H, -OCH_3_), 3.81 (s, 3H, -OCH_3_), 3.84 (s, 3H, -OCH_3_), 6.98 (d, *J* = 8.5 Hz, 2H, H-3′,6′), 7.15 (d, *J* = 16.0 Hz, 1H, H-α), 7.47 (d, *J* = 16.0 Hz, 1H, H-β), 7.69 (d, *J* = 8.5 Hz, 2H, H-2′,6′), 10.91 (s, 1H, -OH); ^13^C NMR (125 MHz): δ 55.6, 60.6, 62.4, 103.6, 103.9, 114.8, 114.8, 120.3, 125.1, 126.9, 131.0, 146.1, 153.9, 155.5, 156.5, 161.9, 192.1; ESI-MS: calculated for C_18_H_16_Br_2_O_5_, *m*/*z* = 469.9364; found: 469.9286.

(*E*)-1-(3,5-Dibromo-2-hydroxy-4,6-dimethoxyphenyl)-3-(p-tolyl)prop-2-en-1-one (**2e**)

Yellow solid (1.50 g, 58%), mp. 151–152 °C; ν_max_ (ATR) 502, 637, 705, 812, 962, 812, 962, 1095, 1196, 1326, 1388, 1541, 1625, 2919 cm^−1^; ^1^H NMR (500 MHz): δ 2.48 (s, 3H, -CH_3_), 3.71 (s, 3H, -OCH_3_), 3.84 (s, 3H, -OCH_3_), 7.21 (d, *J* = 16.0 Hz, 1H, H-α), 7.24 (d, *J* = 8.5 Hz, 2H, H-3′,5′), 7.46 (d, *J* = 16.0 Hz, 1H, H-β), 7.62 (d, *J* = 8.5 Hz, 2H, H-2′,6′), 10.84 (s, 1H, -OH); ^13^C NMR (125 MHz): δ 21.3, 60.6, 62.4, 103.5, 103,9, 120.2, 126.5, 129.0, 129.8, 131.7, 141.3, 145.8, 154.1, 155.6, 156.8, 192.2; ESI-MS: calculated for C_18_H_16_Br_2_O_4_, *m*/*z* = 453.9415; found: 453.9337.

(*E*)-1-(3,5-Dibromo-2-hydroxy-4,6-dimethoxyphenyl)-3-(4-isopropylphenyl)prop-2-en-1-one (**2f**)

Yellow solid (1.62 g, 59%), mp. 128–130 °C; ν_max_ (ATR) 544, 688, 832, 934, 1047, 1089, 1182, 1326, 1385, 1541, 1628, 2930 cm^−1^; ^1^H NMR (500 MHz): δ 1.19 (d, *J* = 8.5 Hz, 6H, 2 x -CH_3_), 2.91 (sept, *J* = 8.5 Hz, 1H, -CH), 3.70 (s, 3H, -OCH_3_), 3.83 (s, 3H, -OCH_3_), 7.22 (d, *J* = 16.0 Hz, 1H, H-α), 7.29 (d, *J* = 8.5 Hz, 2H, H-3′,5′), 7.46 (d, *J* = 16.0 Hz, 1H, H-β), 7.65 (d, *J* = 8.5 Hz, 2H, H-2′,6′), 10.85 (s, 1H, -OH); ^13^C NMR (125 MHz): δ 23.7, 33.6, 60.7, 62.4, 103.6, 103.9, 120.1, 126.5, 126.6, 127.2, 127.3, 129.2, 129.3, 132.1, 132.1, 145.9, 152.1, 154.2, 155.7, 156.9, 192.2; ESI-MS: calculated for C_20_H_20_Br_2_O_4_, *m*/*z* = 481.9728; found: 481.9647.

### 2.3. Typical Method for the Synthesis of the 6,8-Dibromo-5,7-dimethoxyflavones ***3a***–***f***

A stirred mixture of chalcone **2a** (0.2 g, 0.45 mmol) and iodine (10%) in DMSO (30 mL) was refluxed for 30 min. with thin layer chromatography (TLC) monitoring. The mixture was quenched with ice-cold water. The precipitate was filtered on a sintered glass funnel and washed with a saturated aqueous solution of sodium thiosulphate to remove excess iodine. The crude product was recrystallized from a dichloromethane–methanol mixture to afford **3a**. The flavone derivatives **3a**–**f** below were prepared in this fashion.

6,8-Dibromo-5,7-dimethoxy-2-phenyl-4*H*-chromen-4-one (**3a**)

White solid (0.12 g, 58%), mp. 216–218 °C; ν_max_ (ATR) 555, 685, 764, 920, 1097, 1267, 1360, 1450, 1572, 1654, 2938, 3063 cm^−1^; ^1^H NMR (500 MHz): δ 3.91 (s, 3H, -OCH_3_), 4.04 (s, 3H, -OCH_3_), 7.05 (s, 1H, H-3), 7.71 (m, 3H, H-3′,5′ and H-4), 8.22 (d, *J* = 8.5 Hz, 2H, H-2′,6′); ^13^C NMR (125 MHz): δ 61.4, 62.1, 103.7, 108.5, 111.6, 117.0, 126.7, 129.7, 130.8, 132.5, 154.1, 156.6, 158.6, 161.2, 175.3; ESI-MS: calculated for C_17_H_12_Br_2_O_4_, *m*/*z* = 439.9082; found: 439.9181.

6,8-Dibromo-2-(4-fluorophenyl)-5,7-dimethoxy-4*H*-chromen-4-one (**3b**)

White solid (0.12 g, 61%), mp. 288–289 °C; ν_max_ (ATR) 552, 691, 835, 920, 1100, 1233, 1340, 1416, 1507, 1654, 2938, 3071 cm^−1^; ^1^H NMR (500 MHz): δ 3.82 (s, 3H, -OCH_3_), 3.92 (s, 3H, -OCH_3_), 7.05 (s, 1H, H-3), 7.45 (t, *J* = 8.9 Hz, 2H, H-3′,5′), 8.18 (dd, *J* = 5.3 and 8.9 Hz 2H, H-2′,6′); ^13^C NMR (125 MHz): δ 61.4, 62.1, 103.7, 108.4, 111.7, 116.9 (d, ^2^*J*_C-F_ = 22.5 Hz), 127.4, 129.4 (d, ^3^*J*_C-F_ = 8.8 Hz), 154.0, 156.6, 158.6, 160.3, 164.8 (d, ^1^*J*_C-F_ = 248.8 Hz), 175.3; ^19^F NMR (470 MHz, CDCl_3_): δ −107.6; ESI-MS: calculated for C_17_H_11_Br_2_FO_4_, *m*/*z* = 457.8988; found: 457.9086.

6,8-Dibromo-2-(4-chlorophenyl)-5,7-dimethoxy-4*H*-chromen-4-ONE (**3c**)

White solid (0.15 g, 74%), mp. 280–283 °C; ν_max_ (ATR) 473, 555, 688, 829, 1013, 1100, 1337, 1414, 1569, 1654, 2941, 3065 cm^−1^; ^1^H NMR (500 MHz): δ 3.97 (s, 3H, -OCH_3_), 4.07 (s, 3H, -OCH_3_), 7.22 (s, 1H, H-3), 7.81 (d, *J* = 8.5 Hz, 2H, H-3′,5′), 8.26 (d, *J* = 8.5 Hz, 2H, H-2′,6′); ^13^C NMR (125 MHz): δ 60.8, 61.8, 103.2, 108.1, 111.9, 116.6, 127.2, 128.9, 129.3, 138.0, 153.6, 156.8, 158.8, 160.4, 175.4; ESI-MS: calculated for C_17_H_11_Br_2_ClO_4_, *m*/*z* = 473.8692; found: 473.8791.

6,8-Dibromo-5,7-dimethoxy-2-(4-methoxyphenyl)-4*H*-chromen-4-one (**3d**)

White solid (0.14 g, 71%), mp. 233–236 °C; ν_max_ (ATR) 552, 694, 829, 942, 1027, 1097, 1247, 1360, 1569, 1649, 2851, 2947 cm^−1^; ^1^H NMR (500 MHz): δ 3.91 (s, 3H, -OCH_3_), 3.97 (s, 6H, 2 x -OCH_3_), 6.94 (s, 1H, H-3), 7.24 (d, *J* = 8.5 Hz, 2H, H-3′,5′), 8.17 (d, *J* = 8.5 Hz, 2H, H-2′,6′); ^13^C NMR (125 MHz): δ 56.0, 61.4, 62.0, 98.5, 103.6, 106.9, 111.5, 115.2, 116.9, 122.9, 128.6, 156.6, 158.0, 158.4, 161.4, 175.1; ESI-MS: calculated for C_18_H_14_Br_2_O_5_, *m*/*z* = 469.9188; found: 469.9286.

6,8-Dibromo-5,7-dimethoxy-2-(*p*-tolyl)-4*H*-chromen-4-one (**3e**)

White solid (0.14 g, 71%), mp. 205–206 °C; ν_max_ (ATR) 479, 555, 694, 826, 962, 1112, 1289, 1352, 1445, 1555, 1642, 2936 cm^−1^; ^1^H NMR (500 MHz): δ 2.53 (s, 3H, -CH_3_), 3.97 (s, 3H, -OCH_3_), 4.07 (s, 3H, -OCH_3_), 7.12 (s, 1H, H-3), 7.54 (d, *J* = 8.5 Hz, 2H, H-3′,5′), 8.13 (d, *J* = 8.5 Hz, 2H, H-2′,6′); ^13^C NMR (125 MHz): δ 21.3, 61.1, 61.8, 103.3, 107.6, 111.2, 116.7, 126.4, 127.7, 130.0, 142.6, 153.8, 156.4, 159.1, 161.2, 175.0; ESI-MS: calculated for C_18_H_14_Br_2_O_4_, *m*/*z* = 453.9238; found: 453.9337.

6,8-Dibromo-5,7-dimethoxy-2-(4-isopropylphenyl)-4*H*-chromen-4-one (**3f**)

White solid (0.11 g, 57%), mp. 165–167 °C; ν_max_ (ATR) 561, 651, 688, 832, 922, 968, 1100, 1349, 1416, 1558, 1651, 2868 cm^−1^; ^1^H NMR (500 MHz): δ 1.36 (d, *J* = 8.5 Hz, 6H, 2 x -CH_3_), 3.11 (sept, *J* = 8.5 Hz, 1H, -CH), 3.95 (s, 3H, -OCH_3_), 4.05 (s, 3H, -OCH_3_), 7.10 (s, 1H, H-3), 7.59 (d, *J* = 8.5 Hz, 2H, H-3,5), 8.16 (d, *J* = 8.5 Hz, 2H, H-2′,6′); ^13^C NMR (125 MHz): δ 24.2, 34.1, 61.7, 62.3, 103.9, 108.2, 111.8, 117.3, 127.1, 128.0, 128.7, 153.7, 154.3, 156.9, 158.8, 161.8, 175.5; ESI-MS: calculated for C_20_H_18_Br_2_O_4_, *m*/*z* = 481.9551; found: 481.9650.

### 2.4. Single-Crystal X-ray Diffraction Data Collection and Refinement for ***2a***

Intensity data for this compound was determined on a Bruker D8 Venture Microfocus equipped with a Photon III CCD area detector diffractometer with graphite-monochromated MoKa_1_ (l = 0.71073 Å) radiation at 173 K using an Oxford Cryostream 600 cooler (Oxford Cryosystems, Oxford, UK). Data reduction, empirical absorption corrections and space group assignments were carried out using the program SAINT+, version 6.02. The empirical absorption corrections were made using SADABS (Version 2, Bruker AXS Inc., Madison, WI, USA, 2016). The crystal structure of this compound was solved in the WinGX [34] Suite of programs, using intrinsic phasing through SHELXT-2018/2 [35]. The data were refined using full-matrix least-squares/difference Fourier techniques on F^2^ using SHELXL-2019/3 [36]. All the carbon-bound hydrogen atoms were placed at idealized positions and refined as riding atoms with isotropic parameters 1.2 or 1.5 times those of their parent atoms. The oxygen-bound hydrogen atom, on the other hand, was located in the difference Fourier Map. Its fractional coordinates and isotropic displacement parameters were refined freely. The diagrams and publication records for this compound were generated using ORTEP-3 [35] and PLATON [37]. The X-ray analysis, crystal data and structure refinement for **2a** are included as Table S1 of the Supplementary Materials.

### 2.5. Enzyme Inhibition Studies

#### 2.5.1. In Vitro α-Glucosidase Inhibitory Assay of **2a**–**f** and **3a**–**f**

The consumables used for this assay, namely, α-glucosidase type 1 from *Saccharomyces cerevisiae* (Baker’s yeast, G5003), *p*-nitrophenyl-α-D-glucopyranoside (PNP-G, N1377) and acarbose (A8980) were purchased from Merck Life Science (Pty) Ltd. (Modderfontein, Johannesburg, South Africa). The assays and analyses were performed in triplicates following a method described in a previous study [38]. The stock solutions (200 μM) of the test compounds **2**, **3** and acarbose were prepared in DMSO, followed by dilution with a 100 mM phosphate buffer to obtain the concentrations of 1, 5, 10, 25 and 50 μM. A solution of α-glucosidase type 1 from *Saccharomyces cerevisiae* (0.48 u/mL, 17 μL), 100 mM phosphate buffer (pH 6.8; 50 μL) and the test sample in DMSO (17 μL) were incubated at 37 °C for 10 min. This pre-incubation was followed by addition of 2 mM *p*-nitrophenyl-α-D-glucopyranoside (PNP-G, 17 μL) to each of the wells containing reaction mixtures to initiate the reaction. The plate was then incubated for 30 min. at 37 °C. The five absorbance readings were recorded at a wavelength of 405 nm for each triplicate run using a Varioskan flash microplate spectrophotometer (Thermo Scientific, Waltham, MA, USA). The average values obtained from the readings were used to determine the IC_50_ and standard deviation values. The values were calculated by the nonlinear regression analysis using the Graph Pad Prism version 8.0.1 software (San Diego, CA, USA) and expressed as the mean SD of three distinct experiments.

#### 2.5.2. In Vitro α-Amylase Inhibitory Assay of Selected Compounds **2** and **3**

This assay was performed in triplicate using a 96-well plate following the procedure outlined in the α-Amylase Inhibitor Screening Kit (Catalog No. ab283391; Abcam (Cambridge, UK) as described in a previous study [38]. The stock solutions (1000 μM) of the test compounds and acarbose were prepared in DMSO and further diluted with α-amylase assay buffer to obtain a final concentration of 200 μM. These samples were further diluted in the 96-well plate to produce final concentrations of 1, 5, 10, 25 and 50 μM. A solution of α-amylase enzyme (50 μL) was prepared by adding the assay buffer (490 μL) to α-amylase enzyme (10 μL) and then added to each of the wells. The mixtures were incubated at 37 °C for 10 min. and the substrate prepared in the same fashion as the enzyme was added to the mixtures to initiate the reaction. The plate was then incubated at RT for 25 min. in the dark. Five different absorbance readings were recorded for each triplicate run at a wavelength of 405 nm using a Varioskan flash microplate spectrophotometer (Thermo Scientific, Waltham, MA, USA). The IC_50_ and SD values were calculated using the Graph Pad Prism software.

### 2.6. Antioxidant Activity Assays of ***2a***–***f*** and ***3a***–***f***

#### 2.6.1. Nitric Oxide (NO) Free Radical Scavenging Assays of **2a**–**f** and **3a**–**f**

The in vitro NO radical scavenging activity of compounds **2a**–**f** and **3a**–**f** was measured in triplicate following a protocol described in the literature [38]. Different concentrations (1, 5, 10, 25 and 50 μM) of the test compounds and quercetin (reference standard) were prepared in DMSO. A mixture of the compound (5 μL), 10 mM sodium nitroprusside (20 μL) and the phosphate buffer (5 μL) were incubated at 25 °C in a 96-well plate. The plate was incubated for 2.5 h at 25 °C followed by the addition of the Griess reagent (1.00 g of sulphanilic acid + 0.10 g naphthylethylene diamine dihydrochloride, 20 μL). The mixtures were allowed to stand for 30 min. The absorbance of the color developed during the diazotization of nitrite with sulphanilamide and its subsequent coupling with naphthyl ethylenediamine hydrochloride were observed at 550 nm on a Varioskan flash microplate spectrophotometer. The IC_50_ and SD values were calculated using the Graph Pad Prism software.

#### 2.6.2. An In Vitro SOD Inhibitory Assay on Compounds **2a**, **2b**, **2c**, **3a**, **3c**, **3e** and **3f**

The chalcones (**2a**–**c**) and flavones (**3a**, **3c**, **3e** and **3f**) were selected for in vitro enzymatic assay against superoxide radicals using the superoxie dismutase (SOD) activity assay kit (CS-0009, Sigma-Aldrich^®^, St. Louis, MO, USA; Merck, Darmstadt, Germany). The procedure was carried out in triplicates according to the manufacturer’s protocol with slight modifications by referring to the reported instructions from the literature [38]. The compounds and the positive controls, namely, quercetin and ascorbic acid, were tested in the concentration range of 1, 5, 10, 25 and 50 μM. The SOD enzyme (3 U/mL; 10 μL) was combined with the test samples (10 μL) in a 96-well plate and the mixtures were gently shaken for 10 min. and then allowed to stand at 25 °C for another 10 min. The water-soluble tetrazolium (WST) dye (160 μL) was added to each of the wells followed by the addition of xanthine oxidase (20 μL) to initiate the reaction. The inhibition of SOD activity was determined by the increase in superoxide anions generated by the enzyme xanthine oxidase which reacts with the WST. The absorbance of the mixtures was measured at a wavelength of 450 nm using a microplate reader (VarioSkan Flash, Thermo Fisher Scientific, Finland). The SOD inhibition values were corrected with a blank and the IC_50_ values were calculated using a nonlinear regression algorithm as implemented in the GraphPad Prism software (Version 8).

#### 2.6.3. Metal Ions (Zn^2+^ and/or Cu^2+^) Chelation Assays on **3c** and Quercetin

The metal ion chelating ability of quercetin and the flavone derivative **3c** towards Cu^2+^ and Zn^2+^ ions were evaluated using an ultraviolet-visible (UV-Vis) spectrophotometer following a method described in the literature [39]. A solution of the test compound in methanol alone (60 μM, final concentration) or in the presence of CuCl_2_ or ZnCl_2_ was stirred at RT for 30 min. The absorption spectra were recorded at RT at wavelengths ranging from 200 to 600 nm using a Shimadzu UV/Vis spectrometer, model: UV-1800 240V (Shimadzu Europa GmbH, Albert-Han Strasse 6-10, 47269 Duisburg, Germany).

### 2.7. Cytotoxicity Studies of ***2a***–***c***, ***3a***, ***3c***, ***3e*** and ***3f***

The cytotoxic activity (triplicate run) of the chalcones (**2a**–**c**) and flavones (**3a**, **3c**, **3e** and **3f**) was evaluated in vitro on the MCF-7, A549 and Vero cell lines using the CellTiter-Blue Cell viability assay (Promega, Madison, WI, USA) referring to the reported instructions as described in the literature [40]. The cells were maintained in Dulbecco’s Modified Eagle’s Medium (DMEM, Gibco, Carlsbad, CA, USA) supplemented with 10% fetal bovine serum (FBS) and 1% penicillin/streptomycin in culture flasks and incubated at 37 °C in 5% CO_2_. When the cells reached 85% confluency, they were detached using 2% trypsin. The cell count was performed using a hand-held automated cell counter (Scapter 3.0™, Merck, Burlington, MA, USA) and then seeded at 1 × 105 cells/well. The well-plates were incubated overnight to allow cell attachment. After 24 h, treatments were administered with different concentrations (1, 5, 10, 25, 50 and 100 μM) of the test compounds and the positive controls (quercetin and nintedanib). The mixtures were incubated for another 24 h and then 20 μL of the CellTiter-Blue (Cytek Biosciences, San Diago, CA, USA) solution (5 mg/mL) was added to all the wells. Fluorescence was measured at 560 nm excitation/590 nm emission using an Elisa microplate reader (ThermoFisher Scientific, Vario SkanFlash, Vantaa, Finland). The cell viability of the triplicate results was calculated using the GraphPad Prism software (Version 8, GraphPad Software Inc., San Diego, CA, USA) to fit the nonlinear regression dose–response curves to obtain the IC_50_ and standard deviation values.

### 2.8. Molecular Docking Studies of ***2a***–***f*** and ***3a***–***f*** into α-Glucosidase and α-Amylase

The X-ray crystal structures of α-glucosidase and α-amylase in complex with acarbose were obtained from RCSB PDB (with accession number 5NN8 and 5E0F, respectively). The polar hydrogen atoms, Kollman–Amber united atom partial charges and solvation parameters were added using AutoDockTools [41]. The starting structures of the chalcones **2a**–**f** and flavones **3a**–**f** were generated using Avogadro [42]. The united-atom format, Gasteiger charges and torsional angles of the compounds were assigned using AutoDockTools. The grid box for α-glucosidase was centered at −12.175, −35.415, 88.753 (Cartesiam coordinates) and for α-amylase at −7.946, 10.438, −21.863 (Cartesiam coordinates) with 50 × 50 × 50 grid points and 0.375 Å grid spacing. All test compounds were subjected to 100 docking runs using the Lamarckian genetic algorithm employed in AutoDock4.2.6 [41]. Other docking parameters were as follows: energy evaluation of 2,500,000, generation of 27,000, population of 150, crossover rate of 0.8 and mutation rate of 0.02. The interaction between the enzyme and test compound was analyzed using Protein–Ligand Interaction Profiler [43] on the most favorable binding free energy conformation in the most populated cluster.

### 2.9. Pharmacokinetic of Compounds ***2a***–***f*** and ***3a***–***f***

The pharmacokinetic properties and drug-likeness of compounds **2a**–**f** and **3a**–**f** were calculated using Molinspiration (www.molinspiration.com; accessed on 18 July 2024). The Lipinski rule of five was applied in the calculation as follows: less than 5 hydrogen bond donors, less than 10 hydrogen bond acceptors, less than 500 Da molecular mass 500 and less than 5 clogP.

## 3. Results and Discussion

### 3.1. Chemical Synthesis and Characterization of the Chalcones and Flavone Derivatives

**Scheme 1:**
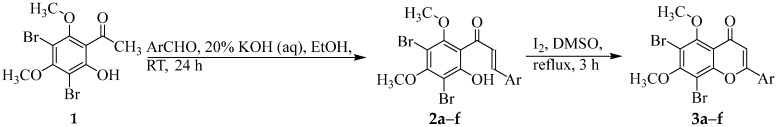
Synthesis and transformation of the chalcones **2a**–**f** into flavone derivatives **3a**–**f**. Ar = -C_6_H_5_ (**a**); -C_6_H_4_(4-F) (**b**); -C_6_H_4_(4-Cl) (**c**); -C_6_H_4_(4-OCH_3_) (**d**); -C_6_H_4_(4-CH_3_) (**e**) and -C_6_H_4_(4-CH(CH_3_)_2_) (**f**).

Chemists continue to adopt and/or improve the existing methodologies to synthesize various kinds of chalcones and flavone derivatives with therapeutic potential [44]. The initial base-mediated Claisen–Schmidt condensation of the known 3,5-dibromo-2-hydroxy-4,6-dimethoxyacetophenone **1** [33] with benzaldehyde derivatives and the subsequent iodine–dimethyl sulfoxide-promoted cyclization of the chalcone intermediates **2** afforded the corresponding 6,8-dibromo-5,7-dimethoxyflavones **3** in 47–71% yield (Scheme 1). The observed yields for the Claisen–Schmidt condensation can be rationalized in terms of the influence of the substituents on the benzaldehyde ring on the electrophilicity of the carbaldehyde carbon. The strong π-electron-donating effect of the 4-methoxy group on the scaffold of **2d** or the relatively weak inductive electron-donating effects of the 4-methyl or 4-isopropyl group of **2e** and **2f**, respectively, reduced the electrophilicity of the carbonyl carbon, resulting in a reduced yield of these chalcones compared to **2a**. Similar observations were reported for different *para*-substituted benzaldehydes with acetophenones [45]. The strong electron-withdrawing inductive effect of chlorine atom at the *para* position of the benzaldehyde ring activated the carbonyl carbon, resulting in an increased yield of the chalcone derivative **2c**. Although a strong electron-withdrawing atom by inductive effect operated through the sigma bond, the resonance effect of the moderate π-electron-donating fluorine atom predominated. This effect reduces the electrophilic character of the carbonyl carbon and, in turn, favors electrophilic aromatic substitution reaction/s on the ring more so compared to the other halogens [46]. Increased electron density on the carbonyl carbon makes it less susceptible to nucleophilic attacks resulting in a reduced yield of the chalcone derivative. The transformation of the chalcone derivatives **2** to the flavone derivatives **3**, on the other hand, was generally accompanied by an increased yield. The chalcone derivatives **2** were easily distinguished from the corresponding precursors by their intense yellow color and the presence of increased signals in the aromatic region of their ^1^H NMR spectra due to the incorporated B-ring. The chalcones can exist in either *E* or *Z* isomeric form around the Cα=Cβ moiety; however, the *trans* geometry is thermodynamically more stable and the most favorable conformation in most cases [47]. A set of doublets which resonates around δ = 7.25 ppm and δ = 7.48 ppm with a vicinal coupling constant value (*J*vic) of 16.0 Hz corresponds to the olefinic protons. This vicinal coupling constant value is characteristic of the *E* (*trans*) geometry about the olefinic bond. A singlet observed significantly downfield in the region δ = 10.72–10.91 ppm corresponds to the intramolecularly hydrogen-bonded hydroxyl group. The infrared spectra of these chalcones included as Figure S1 of the Supplementary Materials revealed the carbonyl group vibration in the region ν_C=O_ = 1620–1631 cm^−1^ due to its participation in intramolecular hydrogen bonding interaction. The observed significant red shift of the infrared X–H stretching frequency in the solid state to below 3000 cm^−1^ is attributed to the increased X–H bond length upon intramolecular hydrogen bond formation to form a six-membered ring-like motif [48]. The O–H stretching bands of these *ortho*-hydroxycarbonyl compounds are generally inconspicuous and the band center was not possible to localize due to strong intramolecular bonding interaction [48]. The aromatic C–H stretch for the chalcone **2b** and its flavone derivative **3b** bearing a *para*-fluorobenzene group as the B-ring is significantly red-shifted compared to the other derivatives. It has previously been observed that the strong electron-withdrawing inductive effect of a fluorine atom in fluorobenzenes reduces the C–H bond length and the corresponding vibrational stretching frequency [49]. The ^1^H NMR spectra of the cyclic derivatives **3** revealed a singlet around δ = 6.97 ppm for the proton at the C-3 position and lacked the singlet significantly downfield for OH observed in the spectra of the corresponding chalcones.

**Figure 2:**
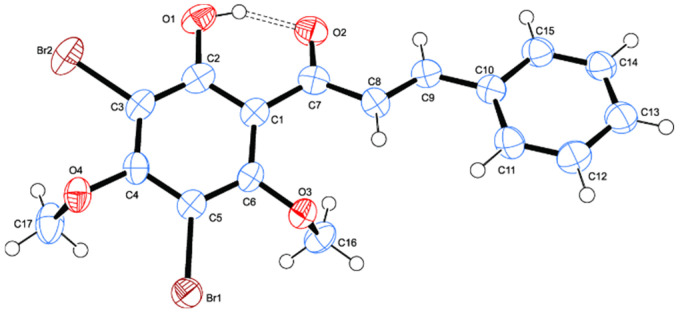
X-ray crystal structure of **2a** showing crystallographic numbering (CCDC 2374158). For clarity, the hydrogen atoms on this scaffold are not labelled.

We obtained crystals for **2a** of a quality suitable for single-crystal XRD analysis. XRD analysis showed that the compound crystallized in a monoclinic space group *P*21n. The conjugated scaffold and the respective atoms bonded directly to it are coplanar with both groups about the carbon–carbon double bond of the α,β-unsaturated carbonyl framework in *trans* geometry (Figure 2). The aromatic-assisted intramolecular hydrogen bonding interaction between the hydroxyl and the carbonyl groups (O(1)-H(1)…O(2) bond distance = 2.486(7) Å) formed a thermodynamically favored six-membered ring-like motif, which restricts conformational changes of the molecule. Such rigidification of the conformation tends to favor the alignment of drug molecules with the protein pocket, resulting in increased ligand–receptor interactions, lipophilicity, membrane permeability and pharmacological activity [50]. Hitherto, the chalcones substituted with halogen (F, Cl or Br) atom, hydroxyl, methoxyl, methyl or nitro group were evaluated for inhibitory activity against α-glucosidase and α-amylase [51].

The antidiabetic activities of the chalcones [51,52] and chrysin derivatives [53] prompted us to evaluate compounds **2a**–**f** and **3a**–**f** for antihyperglycemic properties in vitro against α-glucosidase and α-amylase activities. The free radical scavenging potential of both series of compounds was evaluated spectrophotometrically using the nitric oxide radical (NO^•^) scavenging assay. The structure activity relationship of the two series of compounds has been rationalized with respect to the nature of substituent on the aryl (Ar) group of the open-chain α,β-unsaturated carbonyl scaffold and that of the rigid flavone framework.

### 3.2. Biological Activity Evaluation of Chalcones (***2a***–***f***) and Flavones (***3a***–***f***) with SAR

#### 3.2.1. Inhibition of α-Glucosidase

The inhibitory activity of the chalcones **2a**–**f** and the flavone derivatives **3a**–**f** was first evaluated against α-glucosidase from Baker’s yeast (*Saccharomyces cerevisiae*). Although the yeast-based α-glucosidases differ considerably from the human maltase–glucoamylase and sucrase–isomaltase enzymes, the in vitro enzymatic assays against α-glucosidase are routinely conducted on the enzyme derived from this yeast species [4,51]. The half-maximal inhibitory concentration (IC_50_) values of the test compounds are summarized in Table 1 and these values were determined in the concentration range 1, 5, 10, 25 and 50 μM of the ligand. Acarbose used as a positive control for both assays is a dual inhibitor of these carbohydrate-hydrolyzing enzymes with a higher inhibitory effect against pancreatic α-amylase compared to intestinal α-glucosidase. The chalcones **2a**–**f** and flavone derivatives **3a**–**f** exhibited varied degrees of α-glucosidase inhibition compared to the positive control (8.3 ± 0.002 μM) with the IC_50_ values ranging from 7.3 ± 0.004 μM to 27.4 ± 0.020 μM and from 0.8 ± 0.002 μM to 19.5 ± 0.005 μM, respectively. Compound **2a** with the basic chalcone framework among the series exhibited moderate and comparable activity to the corresponding flavone derivative **3a** against α-glucosidase with the IC_50_ values of 14.5 ± 0.010 μM and 14.2 ± 0.005 μM, respectively. The presence of strong inductive electron-withdrawing, but moderately π-electron-delocalizing fluorine atom at the *para* position of β-phenyl ring resulted in significant activity against this enzyme (IC_50_ = 6.9 ± 0.004 μM) compared to the flavone derivative **3b** (IC_50_ = 19.5 ± 0.005 μM). It is envisaged that the increased conjugative effect and co-planarity of the chalcone scaffold of **2b** resulted in increased aromatic—aromatic (π–π, π–H) interactions with the residues in the active site of this enzyme to inhibit its activity. In contrast, the presence of a relatively weak π-electron-delocalizing chlorine atom at the *para* position of ring-B of **2c** and its flavone derivative **3c** resulted in significant and strong inhibitory effect against α-glucosidase with the IC_50_ values of 12.3 ± 0.003 μM and 0.8 ± 0.002 μM, respectively. The literature precedent revealed that the electron-withdrawing inductive effect of the chlorine atom increases the lipophilicity of the adjacent phenyl group and also the overall lipophilicity of the organochlorine molecule, leading to increased adsorption to enzymes or proteins [54]. Moreover, this halogen atom is capable of engaging in hydrogen bonding and/or halogen bonding (XB) interactions to stabilize the drug–receptor interactions [29]. The presence of a strong π-electron-donating methoxy group on the *para*-position of the 2-phenyl ring of **2d** and **3d** resulted in moderate inhibition against this enzyme with the IC_50_ values of 16.3 ± 0.02 μM and 15.2 ± 0.002 μM, respectively. The presence of a hydrophobic methyl group on the scaffold of chalcone **2e** resulted in a reduced inhibitory effect against α-glucosidase (IC_50_ = 27.4 ± 0.020 μM). The corresponding flavone derivative **3e**, on the other hand, exhibited significant inhibitory activity against this enzyme with an IC_50_ value of 10.9 ± 0.007 μM. The observed significant inhibitory effect may be due to the presence of the methyl group, which is reported to have the capacity to induce a conformational change in the molecule to modulate the biological activity, selectivity, solubility, metabolism and pharmacokinetic or pharmaco-dynamic properties of the drug molecule [55]. The presence of a relatively bulky hydrophobic isopropyl group at the *para*-position of ring-B of flavone **3f**, on the other hand, resulted in a strong inhibitory effect against α-glucosidase (IC_50_ = 5.6 ± 0.006 μM) compared to its chalcone precursor **2f** (IC_50_ = 23.6 ± 0.100 μM). The steric effect of the isopropyl group probably induced a conformational change in the adjacent phenyl ring relative to the chromone scaffold to favor effective interactions in the α-glucosidase active site. α-Glucosidase inhibitors are the most effective in the management of T2DM to reduce PPHG as they increase the sensitivity of insulin to release the stress on the islet β-cells and thus slow down the progression of this metabolic disorder [5,56]. However, partial inhibition of multiple targets is preferable over complete inhibition of a single target to maintain the balance between the normal physiological functions of protein targets and prevention of the progression of the disease [57]. Moreover, better efficacy against T2DM and significantly reduced side effects require antihyperglycemic agents with a dual inhibitory effect against α-glucosidase and α-amylase activity, preferably exhibiting a moderate α-amylase inhibitory effect. Consequently, we evaluated the strong anti-α-glucosidase chalcones **2a**–**c** and flavone derivatives **3a**, **3c**, **3e** and **3f** for inhibitory activity against α-amylase using acarbose as a positive control.

#### 3.2.2. Inhibition of α-Amylase

The chalcone derivative **2a** exhibited a significant inhibitory effect against α-amylase (IC_50_ = 7.3 ± 0.110 μM) compared to acarbose (IC_50_ = 5.2 ± 0.330 μM) a strong inhibitor of this enzyme’s activity. Its flavone derivative **3a** exhibited strong inhibition of α-amylase (IC_50_ = 4.9 ± 0.190 μM) and moderate inhibitory effect against α-glucosidase (IC_50_ = 14.2 ± 0.005 μM). The 3-(4-fluorophenyl) substituted chalcone derivative **2b** exhibited comparable inhibitory activity against α-glucosidase and α-amylase with IC_50_ values of 7.3 ± 0.004 μM and 6.5 ± 0.070 μM, respectively. The chalcone derivatives **2a** and **2b** probably penetrated the relatively deeper cavity of α-amylase to interact with the catalytic residues inside this enzyme’s active pocket resulting in the observed significant inhibitory effect against α-amylase among this series of open-chain α,β-unsaturated carbonyl compounds. A moderate inhibitory effect against α-amylase (IC_50_ = 18.0 ± 0.060 μM) was observed for the 4-chloro substituted chalcone **2c**. The flavone derivative **3c** with strong anti-α-glucosidase activity also exhibited a significant inhibitory effect against α-amylase with an IC_50_ value of 6.3 ± 0.120 μM. Hitherto, the analogous 5,7,8,4′-tetramethoxyflavone and 5,6,7,8,3′,4′-hexamethoxyflavone (nobiletin) exhibited a stronger inhibitory effect against α-glucosidase and moderate activity against α-amylase compared to acarbose [58]. The 2-(4-methylphenyl) **3e** and the 2-(4-isopropylphenyl) substituted flavone **3f** with significant and strong anti-α-glucosidase activity exhibited a modest inhibitory effect against α-amylase among the series with the IC_50_ values of 25.4 ± 0.040 μM and 28.0 ± 0.060 μM, respectively. The selectivity index (SI = IC_50_(α-glucosidase)/IC_50_(α-amylase)) values for the chalcones **2a**, **2b** and **2c** are 1.99, 1.12 and 0.68, respectively. The chalcone **2a** and its flavone derivative **3a** have SI values of 1.99 and 2.90, respectively, which are higher than the value for the antidiabetic drug, acarbose (SI = 1.60). Acarbose is known to occupy the catalytic active sites of both α-glucosidase and α-amylase that lead to substantial starch digestion inhibition, thus causing undesirable side effects from carbohydrate dumping [59]. The chalcone **2a** and flavone **3a** with strong inhibition of α-amylase and moderate inhibition of α-glucosidase will probably result in some amounts of non-digested complex carbohydrates and starch accumulating in the gastrointestinal tract and cause similar side effects observed with acarbose. The chalcone derivative **2b** with SI value of 1.12, which is less than that of acarbose has potential to act as a strong antihyperglycemic agent with dual inhibitory activity against these carbohydrate-hydrolyzing enzymes. Chalcone **2c** (SI = 0.68) is also a potential dual inhibitor of α-amylase and α-glucosidase; however, it has moderate antihyperglycemic activity. The corresponding flavone derivative **3c** (SI = 0.13) is a strong inhibitor of both enzymes with a strong inhibitory effect against α-glucosidase compared to the α-amylase in line with the design strategy. A chlorine atom at the *para* position of the B-ring of a flavone scaffold has been identified as one of the structural features required for dual inhibitory effects against α-glucosidase and α-amylase [5]. This flavone derivative will probably suppress the digestion of carbohydrates, in turn delaying glucose uptake. The dual inhibitory effect of this compound against these carbohydrate-hydrolyzing enzymes will probably result in reduced blood sugar levels with minimal or no gastrointestinal side effects. Slow but complete starch digestion may also be achieved with **3e** (SI = 0.43) which exhibited significant and moderate inhibitory activity against α-glucosidase and α-amylase, respectively. The flavone derivatives **3c** and **3e** will probably reduce side effects observed with selective anti-α-glucosidase inhibitors. The flavone derivative **3f** with an SI value of 0.2 is a strong anti-α-glucosidase and weak anti-α-amylase agent. The strong inhibitors of α-glucosidase are recommended as first-line drugs for the treatment of T2DM [60]. Moreover, the strong α-glucosidase inhibitors can also be used for the treatment of several other carbohydrate-mediated human disorders such as obesity, cancer and HIV [61,62].

#### 3.2.3. Evaluation of Compounds **2** and **3** for Nitric Oxide Radical Scavenging Activity

Classically, flavonoids were generally thought to owe most of their biological (e.g., antidiabetic, anticancer, anti-inflammatory, etc.) effects to their strong antioxidant properties due to the presence of the hydroxyl group/s predominantly on the fused benzo (A) ring [20,63]. However, polymethoxylated flavones such as the 5,7,8,4′-tetramethoxyflavone and nobiletin with no hydroxyl group on their scaffolds have been found to exhibit significant free radical scavenging activity and antioxidant capacity [63]. It has since been established that the flavones owe their antioxidant properties or free radical scavenging activity to the extended π-conjugation of the C2–C3 double bond onto the carbonyl group in the heterocyclic ring and the resultant planarity [63]. The substituents on the A-ring of flavones are envisaged not to be directly involved in the radical scavenging mechanism. However, the type of substituent/s on the B-ring is considered as a determinant of flavonoids’ antiradical potency. Despite the free radical scavenging ability and antioxidant capacity of the polymethoxylated flavones, corresponding data for the analogues with one to three methoxy groups and no hydroxyl functionality on their scaffold is not documented in the literature [20]. The aforementioned considerations and the effects of oxidative stress on inducing diabetic complications encouraged us to evaluate the methoxyflavones **3a**–**f** and their corresponding chalcone precursors **2a**–**f** for nitric oxide radical scavenging activity (Table 2). NO is a key regulator of cardiovascular function, metabolism, neurotransmission, immunity, etc. [64,65]. The presence of the relatively less bulky phenyl or 4-fluorophenyl group on the β-position of the α,β-unsaturated carbonyl framework of **2a** or **2b** resulted in significant NO radical scavenging activity compared to quercetin (IC_50_ = 4.8 ± 0.01 μM) among the chalcone series with the IC_50_ values of 11.0 ± 0.01 μM and 6.5 ± 0.005 μM, respectively. Quercetin is a strong scavenger of ROS, including superoxide radical (O_2_^•−^) and RNS like nitric oxide radical (NO^•^). It owes its increased free radical scavenging activity to the OH group at position-3 of the C-ring and the strong π-electron-delocalizing catechol moiety that stabilizes the free radicals through a resonance effect [66]. Despite the lack of hydroxyl group/s on their scaffolds, the flavones **3a**–**f** generally exhibited higher NO^•^ scavenging activity compared to the corresponding chalcone precursors. The flavone derivatives **3b** or **3c** substituted with a moderate π-electron-delocalizing 4-fluorophenyl or a 4-chlorophenyl group at the C-2 position of the chromone scaffold resulted in significant NO^•^ scavenging activity with the IC_50_ values of 5.4 ± 0.002 μM and 4.7 ± 0.020 μM, respectively. Laboratory studies have revealed that a compound exhibiting a higher glucose-lowering effect also has good antioxidant properties [67], which makes **3c** a potential multi-target-directed ligand for the treatment of this multifactorial disease. The flavone derivative **3d** substituted at the C-2 position with a strong π-electron-delocalizing 4-(methoxyphenyl) group also exhibited a significant NO^•^ scavenging effect (IC_50_ = 5.3 ± 0.005 μM) compared to the corresponding open chain precursor **2d** (IC_50_ = 9.5 ± 0.004 μM). The observed NO^•^ scavenging effect of the non-hydroxylated flavones **3** supports a view that the antioxidant activity of flavonoids is influenced by the extended π-conjugation of the β-phenyl-α,β-unsaturated carbonyl moiety and strongly so by the type of the B-ring substitution. The free radical scavenging capabilities of the test flavones are correlated with the different electronic effects (electron-donor and electron-acceptor properties) of the atoms or groups attached to the phenyl substituent. Increased electron density of the chromone scaffold by the moderate (F or Cl) or strong (-OCH_3_) π-electron-delocalizing substituent on the *para*-position of the B-ring resulted in a strong NO radical scavenging effect compared to the derivatives substituted with the relatively weak inductively donating alkyl group at the same position.

#### 3.2.4. Inhibition or Activation of SOD

The cells routinely produce highly reactive superoxide radicals, which are detoxified by SOD enzymes in the body. The SODs catalyze the conversion of the superoxide radical (O_2_^•−^) into hydrogen peroxide (H_2_O_2_) and oxygen (O_2_), thereby regulating ROS generation [68]. These enzymes protect the body against oxidative stress and inhibition of their activity causes the release of cytochrome c and free radical-mediated damage to mitochondrial membranes. In order to circumvent the disadvantage of a single approach towards NO detection [69], we also evaluated compounds **2a**–**c**, **3a**, **3c**, **3e** and **3f** with strong α-glucosidase activity for potential to activate or inhibit the antioxidant activity of SOD. The compounds were subjected to an enzymatic assay in vitro against SOD in the presence of quercetin and ascorbic acid as reference standards (Table 2). Except for the flavone derivative **3c** (IC_50_ = 24.2 ± 0.05 μM), the other test compounds exhibited a strong inhibitory effect against SOD compared to quercetin (IC_50_ = 22.0 ± 0.04 μM) with the IC_50_ values in the range 8.3 ± 0.04–15.1 ± 0.05 μM. Quercetin was previously found to significantly increase the activity of SOD and catalase (CAT) acute myocardial infarction rats [70]. The activity of both series of compounds, on the other hand, was slightly higher (IC_50_ = 8.3 ± 0.04–9.6 ± 0.02 μM) or less (IC_50_ = 11.5 ± 0.01–15.1 ± 0.05 μM) compared to ascorbic acid (IC_50_ = 10.2 ± 0.20 μM). Ascorbic acid previously significantly increased the activity of SOD in cultured cells in vitro and in vivo and this effect resulted in a reduced level of the superoxide radical anion and prevented oxidative stress [71]. The test chalcones and their flavone derivatives will probably form complexes with the enzyme and increase its activity, in turn scavenging superoxide and inhibiting the formation of ROS. SOD enzymes, on the other hand, compete with NO for superoxide anions and inactivate NO to form peroxynitrite. This reaction is accompanied by alternate oxidation–reduction of metal ions, such as Cu^2+^ and Zn^2+^, Fe^2+^, Mn^2+^ and Ni^2+^, which are vital to enzymatic activity and are present in the active site of SODs (CuZnSOD, FeSOD, MnSOD and NiSOD, respectively) [72]. Although essential for enzymatic function, increased levels of these metals in the body, on the other hand, may induce the formation of ROS and RNS, resulting in peroxidation of proteins, DNA, RNA and lipids in the plasma membrane [73]. Published data have shown that flavones possessing only methoxy group/s have the capacity to chelate metallic ions. The proximity of the methoxy group to the carbonyl moiety in the case of the 5-methoxyflavone scaffold has previously been found to facilitate coordination with metal ions such as Zn(II) and Cu(II) to form complexes [31].

#### 3.2.5. Metal Complexation of Flavone **3c**

**Figure 3:**
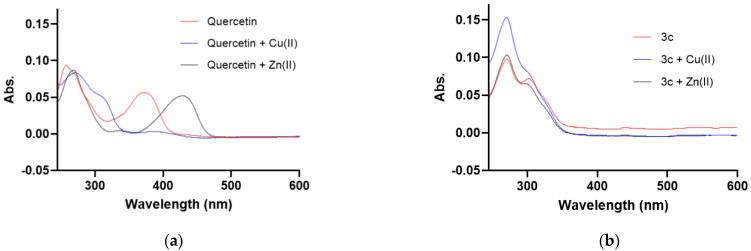
UV-Vis spectra of quercetin (**a**) and flavone **3c** (**b**) alone (final concentration of 60 μM) or in the presence of ZnCl_2_ and CuCl_2_ in methanol (2:1) at RT.

**Scheme 2:**
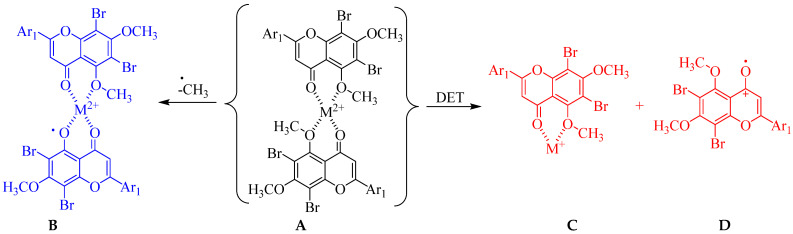
Proposed structure of the metal–5,7-dimethoxychrysin complex **A** and its fragmentation.

We selected the methoxyflavone derivative **3c** as a representative model for the flavone series and evaluated it through an Ultraviolet (Uv)-Visible (Vis) spectrophotometric assay for the ability to bind Cu^2+^ or Zn^2+^ ions. This 5,7-dimethoxy substituted flavone derivative and quercetin exhibited comparable NO radical scavenging and activation of SOD. Quercetin is known to coordinate metal ions through the catechol moiety in its structure, in turn inducing Cu^2+^ to play an antioxidant role. Both these flavonoids were assayed in vitro for their capability of chelating Cu^2+^ or Zn^2+^ ions in the wavelength region of λ = 200–600 nm. The UV-Vis spectra of flavones generally exhibit two major absorption bands in the regions, λ = 320–385 cm^−1^ and 250–285 cm^−1^ [74]. These bands originate from π–π* transitions within the aromatic 3-ring system of the ligand molecule and correspond to the B and A ring, respectively. The absorption spectrum of quercetin (Figure 3a) acquired in methanol at room temperature also showed these characteristic bands at λ = 372 nm and λ = 257 nm, respectively. These bands were also observed at λ = 330 nm and λ = 270 nm in the absorption spectrum of **3c** (Figure 3b). The two bands observed in the spectrum of quercetin merged in the presence of CuCl_2_ to form a broad band around λ = 271 nm due to the changes of the cinnamoyl moiety caused by the formation of a ligand–Cu^2+^ complex. A slight hypochromic shift was observed for the band at λ = 270 in the presence of ZnCl_2_ and another intense band resonated at λ = 430 nm. The latter band, in our view, is the result of the formation of a ligand–metal complex because the ligand does not absorb at this wavelength. The methanol solution of **3c** containing CuCl_2_ resulted in a significant increase in intensity of the maxima at λ = 330 nm and a decrease of the intensity of tne band at λ = 270 nm. Although not effective compared to quercetin with a catechol moiety, the observed changes in intensity of the two maxima, in our view, suggest some coordination of **3c** to the copper ion. The addition of ZnCl_2_ to the methanol solution of **3c**, on the other hand, resulted in a slight reduction in the intensity of the maxima at λ = 330 nm and also a slight hypochromic shift of the band at λ = 270 nm. This observation may suggest that the 5-methoxyflavones have higher affinity for Cu^2+^ compared to Zn^2+^. It is envisaged that by chelating oxidizing metal ions, the 5-methoxy substituted flavones **3** could prevent metal-catalyzed free radical generation, impart antioxidant effects and, in turn, protect the biomolecules from oxidative stress. We propose a mechanism outlined in Scheme 2 to show how these methoxyflavones would form metal complexes that could scavenge free radicals in the body, in turn reducing oxidative stress.

#### 3.2.6. Proposed Mechanism for the Metal Ion Chelation of **3**

The chelation process is envisaged to proceed through a metal–ligand complex of the generalized structure **A**. A similar metal–ligand complex was previously detected in the nanoelectrospray ionization (nano-ESI) LTQ Orbitrap tandem mass spectrum of the copper–5-methoxyflavone [2L+Cu]^2+^ complex [31]. Since the methoxy group is a site for free radical or photochemical reactions [26], a methyl radical is probably extruded from **A** to form M^2+^–complex **B**. Alternatively, the metal complex **A** may undergo a dissociative electron transfer (DET) to form a ligand–pro-oxidant (M^+^) complex **C** and cation–radical **D** [73]. The formation of either the ligand–M^n+^ complexes **B** or the reduction to ligand–pro-oxidant (M^+^) complexes **C** and a cation–radical **D** will prevent the M^+^ (Cu^+^ and Fe^2+^) ions from entering the Fenton (Fe^2+^ + H_2_O_2_ → Fe^3+^ + OH^−^ + ^•^OH) and Haber–Weiss (O_2_^•−^ + H_2_O_2_ → O_2_ + H_2_O + ^•^OH) reactions [74]. It is envisaged that the increased lipophilicity (membrane-penetrating properties) of the methoxyflavonoids will enable these compounds to enter the cell and chelate the heavy metals and also form complexes with SOD enzymes, for example, in turn suppressing the Fenton and Haber–Weiss reactions and therefore the formation of ROS.

### 3.3. Cytotoxicity Assay of the Chalcones ***2*** and Flavone Derivatives ***3***

Inhibition of α-glucosidase has been found to have positive effects in the treatment of different types of cancers [62]. Moreover, preclinical and clinical studies also underscore the link between high circulating glucose levels and cancer initiation, proliferation and invasiveness [75,76]. Indirect effects of hyperglycemia on cancer cells are also mediated by several factors including generation of ROS or oxidative stress [76]. It is envisaged that the drugs that can mitigate PPHG and oxidative stress, and also exhibit anticancer properties may offer an opportunity for development as multi-target-directed ligands (MTDLs). Due to the multifactorial origin of T2DM, we also screened the chalcones (**2a**–**c**) and flavone derivatives (**3a**, **3c**, **3e** and **3f**) for their antiproliferative effect using the MCF-7 and A549 cell lines as models to establish their anticancer properties. Quercetin used as a positive control for these assays has been shown through several in vitro and in vivo studies to retard the proliferation of a wide range of cancers including the brain, breast, cervix, colorectum, lung and prostate cancers [77]. Nintedanib, on the other hand, is a second-line therapeutic agent for the treatment for lung cancer [78] and this drug has also exhibited efficacy in early clinical trials for HER2-negative breast cancer [79]. The half-maximal inhibitory concentration (IC_50_) values determined in the concentration range, 1, 5, 10, 25, 50 and 100 μM, with respect to the MCF-7 and A549 cell lines are summarized in Table 2. The percentage inhibition curves used to calculate these IC_50_ values are included as Figures S6–S8 of the Supplementary Materials, respectively. The chalcones (**2a**–**c**) and flavones (**3a**, **3c**, **3e** and **3f**) exhibited moderate cytotoxicity against the MCF-7 and A549 cell lines compared to the anticancer drug, nintendanib (IC_50_ = 0.53 ± 0.11, 0.74 ± 0.15 and 0.89 ± 0.18 μM, respectively) with IC_50_ values in the range of 4.12 ± 0.55–15.07 ± 0.91 μM and 5.10 ± 0.61–10.66 ± 1.02 μM, respectively, and 7.40 ± 0.67–18.65 ± 0.89 μM and 6.42 ± 0.79–16.15 ± 1.15 μM, respectively. However, both series of compounds exhibited strong cytotoxicity against the MCF-7 and the A549 cell lines compared to quercetin (IC_50_ = 35.40 ± 1.78 and 35.38 ± 1.78, respectively). Compound **2a**, among the tested chalcones, exhibited significant cytotoxicity against the MCF-7 cell line (IC_50_ = 4.12 ± 0.55 μM) and the A549 cell line (IC_50_ = 7.40 ± 0.67 μM). The flavone derivatives **3a** and **3c** reduced the viability of the MCF-7 cells (IC_50_ = 8.50 ± 0.82 μM and 5.10 ± 0.61 μM, respectively) more so compared to the A549 cell line (IC_50_ = 16.23 ± 0.91 μM and 28.09 ± 1.29 μM, respectively). Compound **3c** with a dual inhibitory effect against the carbohydrate-hydrolyzing enzymes and strong NO free radical scavenging activity has potential to treat T2DM and also prevent the growth of breast cancer in women. Significant and comparable cytotoxicity against the MCF-7 (IC_50_ = 6.96 ± 0.66 μM) and the A549 cell line (IC_50_ = 6.42 ± 0.79 μM) were observed for the flavone derivative **3e**. An anticancer agent with a significant or strong cytotoxic effect on cancer cell lines and less effect on non-cancerous cell lines is preferable. Moreover, a stable long-term use of MTDLs for the treatment of T2DM will require such drugs to exert potency against the cancer cells with significantly reduced or no toxicity to the normal cells. Consequently, these compounds were also screened for cytotoxicity against the non-cancerous Vero cell line to establish their safety profile at least in vitro. Although the Vero cells are derived from the kidney of an African green monkey (*Cercopithecus aethiops*), they are routinely used for the screening programs to assess the toxicity of compounds [80,81] or microbial toxins in vitro [82]. Compound **2a**, among the tested chalcones exhibited significant cytotoxicity against the Vero cell line (IC_50_ = 6.04 ± 0.54 μM). A reduced cytotoxicity against the Vero cell line was observed for the flavones **3a** and **3c** with the IC_50_ values of 12.38 ± 0.97 μM and 11.66 ± 1.00 μM, respectively. The IC_50_ value of **3e** against the Vero cell line was established to be 9.45 ± 0.79 μM. Although less cytotoxic against the two cancer cell lines compared to nintedanib, the test chalcones and flavone derivatives generally exhibited reduced cytotoxicity against the normal Vero cells compared to this anticancer agent. The ability of **3c** to kill cancer cells with reduced effect on non-cancerous cells, makes this flavone derivative a suitable MTDL for the treatment of diabetes and cancer-related complications. Although the anticancer activity of flavones could be correlated with their antioxidant activity, the observed anticancer activity of the flavones **3** with no hydroxyl group on their scaffolds, in our view, suggests the possibility of other mechanism/s of action of these methoxyflavones besides the neutralizing effect of free radicals.

Hydrogen bonds, hydrophobicity, electronic distribution, flexibility and the size of molecules affect the bioavailability, toxicity, protein affinity, metabolic stability and transport properties of the drugs in biological systems [83]. These features have also been considered favorable for the inhibition of α-glucosidase and/or α-amylase activities. The aryl rings of the α,β-unsaturated carbonyl derivatives **2** and **3** are decorated with substituents that are more likely to interact with several residues in the active sites of α-glucosidase and α-amylase to enhance biological activity and probably improve their ADME properties. We employed molecular docking (in silico) to obtain a theoretical/hypothetical model for potential binding modes of the test compounds against α-glucosidase and α-amylase to predict the fit and orientation of the test compounds in the active sites of these enzymes.

### 3.4. Molecular Docking (In Silico) Studies

**Figure 4:**
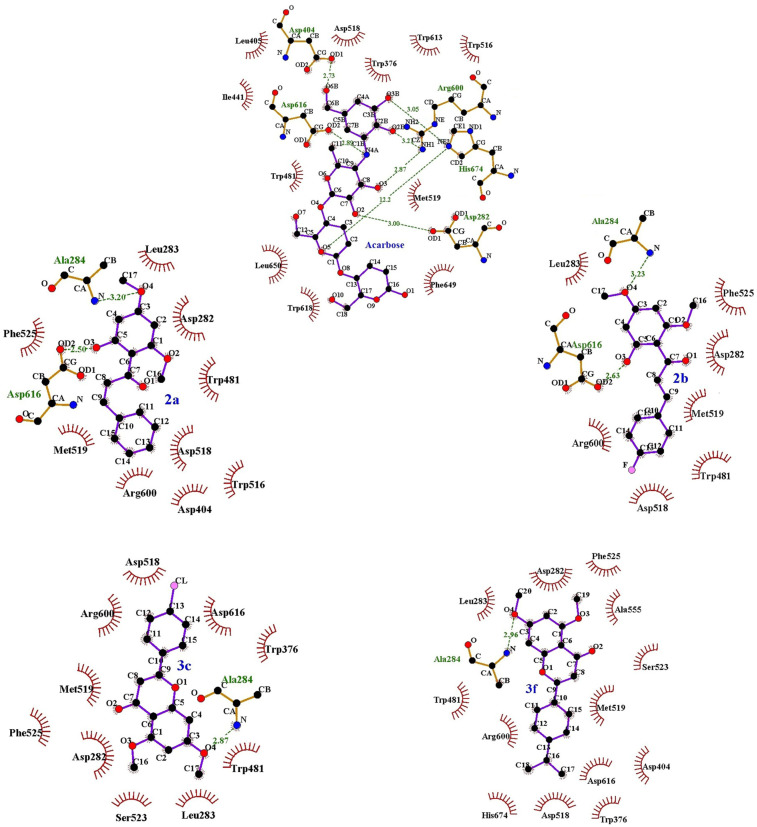
The interactions of acarbose, **2a**, **2b**, **3c** and **3f** with human lysosomal acid-α-glucosidase (PDB: 5NN8) in 2D format. Green dashed line and spoke arc represent hydrogen bonding and hydrophobic interactions, respectively.

The docking simulations were carried out on all the compounds against α-glucosidase and α-amylase to determine the conformation and ligand–protein interactions. Although the in vitro enzymatic assay against α-glucosidase is routinely conducted on the enzyme from Baker’s yeast (*Saccharomyces cerevisiae*) [5], the structure of the yeast α-glucosidase is yet to be solved. Consequently, we docked acarbose and the test compounds into the active site of the human lysosomal acid-α-glucosidase co-crystallized with acarbose with a PDB id 5NN8. The re-docked structure of acarbose into α-glucosidase shows an extensive network of hydrogen bonds between the hydroxyl groups and the amino acid residues, Asp404, Trp481, Asp581, Arg600, Asp616 and His674 as shown in Figure 4. Acarbose also forms eleven hydrophobic interactions with α-glucosidase. The docking results of all the chalcones **2** and their flavone derivatives **3** are represented in Figure S9 and the corresponding binding energy values as Table S2 of the Supplementary Materials. The docking poses of both series of compounds showed a good fit in the active site of α-glucosidase and they also interacted with some of the residues which interacted with acarbose. The interactions of the most active derivatives against α-glucosidase from each series with lower IC_50_ values, namely, the chalcones **2a** and **2b**, and the flavone derivatives **3c** and **3f** as representative models are presented in Figure 4. Molecular docking predicts the hydroxyl and methoxy groups of **2a** and **2b** to be involved in hydrogen bonding interaction with the residues, Asp616 and Ala284. The compounds are also involved in aromatic–aromatic (π–π stacked and/or π–π T-shaped) interactions with several amino acids in the catalytic site of α-glucosidase. The hydrophilic (hydrogen bonds) and hydrophobic (aromatic–aromatic and π–alkyl) interactions of these compounds with the residues in the catalytic site of α-glucosidase probably account for the observed significant or strong inhibitory effect against this enzyme. It has previously been demonstrated that the extended π-conjugation of the C2–C3 double bond onto the carbonyl group in the C-ring of flavones and the resultant planarity allow the selective inhibition of α-glucosidase through entry of the B-ring into the narrow and deep catalytic pocket of this receptor [84]. The flavone **3b** with a fluorine atom on ring-B has the least favorable binding free energy of −6.13 kcal/mol (see Table S2) among the flavones consistent with its reduced inhibitory activity against α-glucosidase. The hydrophobic (π–π stacked and π–alkyl) interactions are also predicted between the flavones and the hydrophobic residues in the active pocket of this enzyme. The scaffold of **3c** (binding free energy = −6.87 kcal/mol) forms a hydrogen bond between the oxygen of the 7-methoxy group and the residue Ala284. This compound is also involved in a complex network of hydrophobic interactions with the residues, Asp282, Leu283, Trp376, Trp481, Asp518, Met519, Ser523, Phe525, Arg600 and Asp616. Binding with the residues Trp376, Phe525 and/or Phe649 could inhibit the glucosidase hydrolysis activity. Hydrogen bonding interaction and the complex hydrophobic network with these residues probably account for the observed increased affinity and increased inhibitory effect of this compound against this enzyme. The flavone derivative **3f** with a bulky hydrophobic isopropyl group in the *para* position of ring-B has the most favorable binding free energy (−7.73 kcal/mol) among the test compounds. A hydrogen bond is predicted between the oxygen atom of the 7-methoxy group of this compound and Ala284. This flavone derivative is also involved in hydrophobic interactions similar to those observed for **3c**. Additional hydrophobic interactions are predicted with the residues Asp404, Ala555 and His674, which may explain the most favorable binding free energy for this flavone and the observed increased inhibitory effect against this enzyme’s activity. It is envisaged that the steric effect of the isopropyl group helped to direct the binding conformation of **3f,** resulting in multiple hydrophobic interactions with residues in the active site of this enzyme. The chalcones and flavone derivatives are predicted to interact with the catalytic residues Asp518 and/or Asp616 in the active site of α-glucosidase, which play an important role in the hydrolysis of glycosidic linkage in sugars [85].

**Figure 5:**
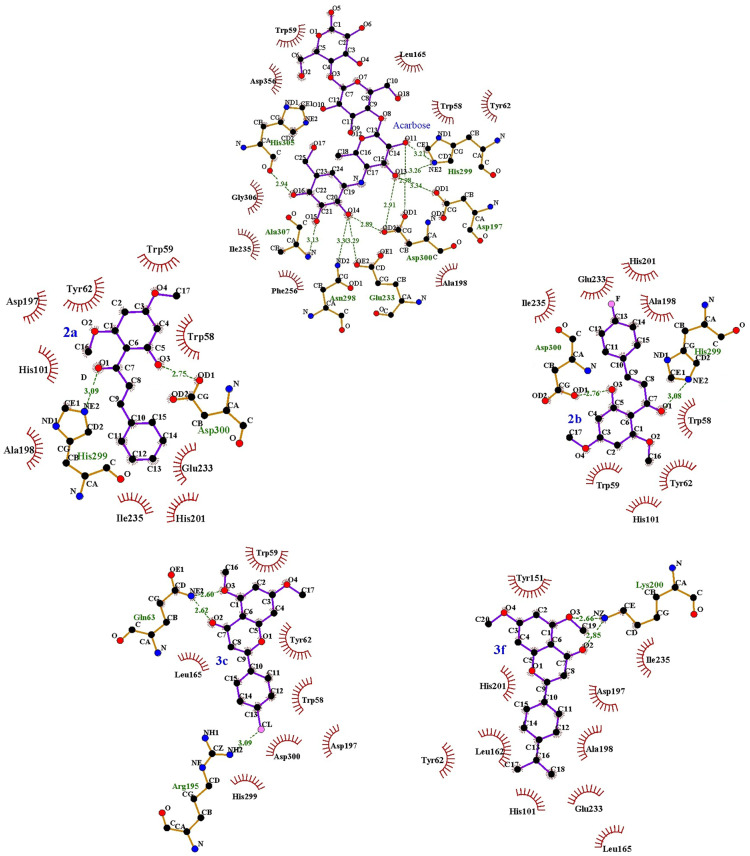
The interactions of acarbose, **2a**, **2b**, **3c** and **3f** with human pancreatic α-amylase (PDB: 5E0F) in 2D format. Green dashed line and spoke arc represent hydrogen bonding and hydrophobic interactions, respectively.

The docked structure of acarbose into the α-amylase binding pocket (Figure 5) shows the hydroxyl groups engaged in several hydrogen bonding interactions with several amino acid residues (Arg195, Asn298, His299, Asp300, His305, Ala307 and Asp356) which are responsible for the hydrolysis of starch [59]. Each of the chalcones **2a** and **2b** forms hydrogen bonds with His299 involving carbonyl oxygen. Bromine atom is known to improve the biological activity and selectivity of the drug molecules [86] and its presence facilitated the hydrogen bonding interaction of **2a** and **2b** with Asp300. These chalcone derivatives are also involved in hydrophobic interactions with the aromatic residues, Trp58, Trp59 and Tyr62 in the catalytic site of this enzyme. These hydrophobic interactions and hydrogen bonding interactions with the residues His299 and Asp300 probably account for the observed increased activity against this enzyme. Hydrogen bonding interaction with bromine, on the other hand, probably accounts for selectivity of these chalcone derivatives against α-amylase. The oxygen atoms of the 5-methoxy and carbonyl group of the flavone **3c** are involved in a bifurcated hydrogen bonding interaction with Gln63. An additional hydrogen bonding interaction is predicted between the chlorine atom of this compound and the residue Arg195. This flavone derivative is also involved in seven hydrophobic interactions with the amino acid residues Trp58, Trp59, Tyr62, Leu165, Asp197, His299 and Asp300. Hydrogen bonding interactions with the residues Gln63 and Arg195 as well as increased hydrophobic interactions also involving the residues His299 and Asp300 probably account for the observed increased activity of this flavone derivative against α-amylase. The flavone derivative **3f** forms a single bifurcated hydrogen bond involving interaction of oxygen atoms of the 5-methoxy and carbonyl group with Lys200. However, this flavone forms the highest number (10) of hydrophobic interactions with α-amylase amongst the test compounds. The reduced number of hydrogen bonds probably accounts for the reduced inhibitory effect of this flavone derivative against α-amylase compared to **3c**. The halogen (chlorine) atom at the *para* position of the B-ring of **3c** or alkyl (isopropyl) group of **3f** helped to direct the binding conformation of the ligand to form multiple π–alkyl interactions (4 and 7, respectively) with the residues in the active site of this enzyme. Increased hydrogen bonding and hydrophobic interactions between acarbose and several residues in the catalytic active sites of α-glucosidase and α-amylase is due to its high number of –OH groups and the large surface area occupied by this antidiabetic drug. These strong interactions probably account for its substantial inhibition of starch digestion and therefore the undesirable side effects due to carbohydrate dumping. The small ligand molecules tested in this study, on the other hand, will occupy a small surface area in the catalytic active sites of these enzymes to engage in hydrogen bonding and several hydrophobic (aromatic–aromatic) interactions with relatively fewer amino acid residues in the active sites of α-glucosidase and α-amylase. This, in our view, makes the test compounds suitable candidates to suppress carbohydrate digestion and delay glucose uptake with minimal or no gastrointestinal side effects.

The drug-likeness features play an important role in assessing the quality of compounds to narrow down the list of candidates for future in vivo studies and/or preclinical testing. The methoxy group can enhance the ligand’s target binding, physicochemical and pharmacokinetic properties [28]. Additionally, the ADME properties of the test chalcones and the flavone derivatives were calculated to predict their drug-likeness. The Lipinski rule of five (hydrogen bond donors ≤ 5, hydrogen bond acceptors ≤ 10, molecular mass < 500 Da and clogP < 5), which describes the relationship between the physicochemical and pharmacokinetic properties of drugs, was applied in the calculation. The corresponding data for the pharmacokinetic and drug-likeness are shown in Table 3. The estimations show that all compounds could be orally bioavailable, although there is a violation of the Lipinski rule of five, with the compounds showing the slightly higher hydrophobicity (poor aqueous solubility) characteristic of polymethoxyflavones [87]. This means that the test compounds can easily cross the phospholipid membrane, which favors transport and cellular uptake (bioavailability). In addition, toxicity prediction has been performed using ProTox 3.0 [88] and the data are represented as Table S3 of the Supplementary Materials. Toxicity is another important concern for the treatment of human disorders such as diabetes, which requires a stable long-term treatment, and therefore drugs with significantly reduced or no toxicity. According to the Globally Harmonized System of Classification and Labelling of Chemicals (GHS), both variants of compounds **2** and **3** are predicted to be within toxicity class V (median lethal dose or LD_50_ = 3000 mg/kg and 2570 mg/kg, respectively) whereby they have relatively low acute toxicity, but may be harmful if swallowed (2000 < LD_50_ ≤ 5000).

## 4. Conclusions

This study has identified the 3,5-dibromo-4,6-dimethoxychalcones (**2a**–**2c**) and the flavone derivatives (**3a**, **3c, 3e** and **3f**) as potential multi-target-ligands with anti-hyperglycemic activity and antioxidant properties. The flavone derivative **3c** also exhibited reduced inhibitory activity against SOD and a significant antigrowth effect against the MCF-7 cell line with reduced cytotoxicity towards the normal Vero cells. This compound will normalize PPHG and prevent complications of T2DM due to oxidative stress and/or cancer with a reduced cytotoxic effect on the normal cells. The proximity of the 5-methoxy group to the carbonyl moiety facilitated the coordination of the flavone derivatives with metal ions to form complexes capable of suppressing the Fenton and Haber–Weiss reactions, in turn inhibiting the formation of ROS. The test compounds will probably form complexes with the SOD enzymes and increase their activity, in turn scavenging superoxides and suppressing the formation of ROS. The structure–activity relationship (SAR) analysis and molecular docking studies of these chalcones and flavones highlight the significance of hydrophilic and hydrophobic interactions towards their inhibition of the two carbohydrate-hydrolyzing enzymes. The presence of the 7-methoxy group on the A-ring enabled the test compounds to form hydrogen bonds and increased hydrophobic interactions with residues in the catalytic site of α-glucosidase and α-amylase. The substituents on the B-ring of these flavones also play an important role in α-glucosidase and α-amylase inhibition and probably helped to direct the binding conformation to form multiple interactions with residues in the active sites of these enzymes. The pharmacokinetic and drug-likeness estimations show that the test compounds could cross the phospholipids membrane and have relatively low acute toxicity.

## Figures and Tables

**Table 1:** The IC_50_ values of the test compounds against α-glucosidase and α-amylase as well as NO and SOD.

Compounds	IC_50_ (μM ± SD)			
	α-Glucosidase	α-Amylase	NO	SOD
**2a**	14.5 ± 0.010	7.3 ± 0.110	11.0 ± 0.010	11.5 ± 0.01
**2b**	7.3 ± 0.004	6.5 ± 0.070	6.5 ± 0.005	9.3 ± 0.03
**2c**	12.3 ± 0.003	18.0 ± 0.060	28.0 ± 0.003	8.3 ± 0.04
**2d**	16.3 ± 0.020	-	9.5 ± 0.004	-
**2e**	27.4 ± 0.020	-	16.1.0 ± 0.030	-
**2f**	23.6 ± 0.100	-	17.4 ± 0.015	-
**3a**	14.2 ± 0.005	4.9 ± 0.190	16.7.0 ± 0.006	12.6 ± 0.04
**3b**	19.5 ± 0.005	-	5.4 ± 0.002	-
**3c**	0.8 ± 0.002	6.3 ± 0.120	4.7 ± 0.020	24.2 ± 0.05
**3d**	15.2 ± 0.002	-	5.3 ± 0.005	-
**3e**	10.9 ± 0.007	25.4 ± 0.040	13.3 ± 0.080	9.6 ± 0.02
**3f**	5.6 ± 0.006	28.0 ± 0.060	12.1 ± 0.070	15.1 ± 0.05
Acarbose	8.3 ± 0.002	5.2 ± 0.330	-	-
Quercetin	-	-	4.8 ± 0.010	22.0 ± 0.04
Ascorbic acid	-	-	-	10.2 ± 0.20

Data expressed as the mean ± SD.

**Table 2:** Cytotoxicity of chalcones **2a**–**c** and flavones **3a**, **3c**, **3e** and **3f** against the MCF-7, A549 and Vero cell lines.

Compounds	IC_50_ (μM ± SD)		
	MCF-7	A549	Vero
**2a**	4.12 ± 0.55	7.40 ± 0.67	6.04 ± 0.54
**2b**	12.11 ± 0.72	18.65 ± 0.89	25.00 ± 1.09
**2c**	15.07 ± 0.91	9.68 ± 0.80	20.28 ± 1.01
**3a**	8.50 ± 0.82	12.38 ± 0.97	16.23 ± 0.91
**3c**	5.10 ± 0.61	11.66 ± 1.00	28.09 ± 1.29
**3e**	6.96 ± 0.66	6.42 ± 0.79	9.45 ± 0.79
**3f**	10.66 ± 1.02	16.15 ± 1.15	11.73 ± 1.02
Quercetin	35.40 ± 1.78	35.38 ± 1.78	45.56 ± 1.80
Nintedanib	0.53 ± 0.11	0.74 ± 0.15	0.89 ± 0.18

**Table 3:** Pharmacokinetics properties calculation of compounds **2** and **3**.

Properties	Compound											
	2a	2b	2c	2d	2e	2f	3a	3b	3c	3d	3e	3f
Number of atoms	23	24	24	25	24	26	23	24	24	25	24	26
Rotatable bonds	5	5	5	6	5	6	3	5	5	6	5	6
Topological polar surface area (TPSA; Å)	55.77	55.77	55.77	65.00	55.77	55.77	48.68	55.77	55.77	65.00	55.77	55.77
Absorption (%) [109 − (0.345 × TPSA)]	89.76	89.76	89.76	86.58	89.76	89.76	92.21	89.76	89.76	86.58	89.76	89.76
Molecular volume	296.73	301.66	310.27	322.28	313.29	346.68	286.86	301.66	310.27	322.28	313.29	346.68
Hydrogen bond donor	4	4	4	5	4	4	4	4	4	5	4	4
Hydrogen bond acceptor	1	1	1	1	1	1	0	1	1	1	1	1
Molecular weight	442.10	460.09	476.55	472.13	456.13	484.18	440.09	460.09	476.55	472.13	456.13	484.18
miLogP	5.04	5.20	5.71	5.09	5.48	6.55	5.23	5.20	5.71	5.09	5.48	6.55
Lipinski’s violation	1	1	1	1	1	1	1	1	1	1	1	1

## Data Availability

The CIF file containing complete information on the studied structure was deposited with the Cambridge Crystallographic Data Center, CCDC 2374158, and is freely available upon request from the following website: www.ccdc.cam.ac.uk (accessed on 9 September 2024). You may also contact the Cambridge Crystallographic Data Center, 12, Union Road, Cambridge CB2 1EZ, UK; Fax: +44-1223-336033; email: deposit@ccdc.cam.ac.u.

## Supplementary Materials

The following supporting information can be downloaded at https://www.mdpi.com/article/10.3390/antiox13101255/s1, Figure S1: Copies of the NMR (^1^H and ^13^C) and IR spectra of compounds **2a**–**f** and **3a**–**f**; Table S1: X-ray analysis, crystal data and structure refinement for **2a**; Figures S2–S5: curves used to calculate the IC_50_ values against α-glucosidase, amylase, NO and SOD; Figure S6: curves used to calculate the IC_50_ values against the MCF-7 and A549; Figure S7: Curves used to calculate the IC_50_ value forVero cell lines; Figures S8 and S9: the interactions of compounds **2** and **3** with α-glucosidase and α-amylase, respectively; Table S2: estimated binding free energies of **2a**–**f** and **3a**–**f**; Table S3: the toxicity prediction of compounds **2** and **3** using ProTox 3.0.
