# Supplementary material for: Exploring the 3,5-Dibromo-4,6-dimethoxychalcones and Their Flavone Derivatives as Dual α-Glucosidase and α-Amylase Inhibitors with Antioxidant and Anticancer Potential
Source: Antioxidants (Basel). 2024 Oct 17;13(10):1255. doi: 10.3390/antiox13101255 (PMC11505200; doi:10.3390/antiox13101255)
Supplement: Supplementary file 1 [file antioxidants-13-01255-s001.zip › antioxidants-3248544-supplementary.pdf]

Supplementary Material

Exploring the 3,5-Dibromo-4,6-dimethoxychalcones and Their Flavone Derivatives as Dual α-Glucosidase and α-Amylase Inhibitors with Antioxidant and Anticancer Potential

J.K. Nkoana, M.J. Mphahlele, G.K. More and Y.S. Choong

Figure S1: Copies of NMR and IR spectra of **2a–f** and **3a–f**
Table S1: Crystal data collection and structure refinement for **2a**
Figure S2: Curves used to calculate the IC_{50} values against α-glucosidase
Figure S3: Curves used to calculate the IC_{50} values α-amylase
Figure S4: Curves used to calculate the IC_{50} values against NO
Figure S5: Curves used to calculate the IC_{50} values against SOD
Figure S6: Curves used to calculate the IC_{50} values against the MCF-7 (a) and A549 (b) cell lines
Figure S7: Curves used to calculate the IC_{50} values against the Vero cell line
Figure S8. The interactions of compounds **2** (a) and **3** (b) with the human lysosomal acid-α-glucosidase (PDB id: 5NN8).
Figure S9. The interactions of compounds **2** (a) and **3** (b) with the pancreatic α-amylase (PDB: 5E0F).
Table S2: Estimated binding free energies of **2a–f** and **3a–f** against α-glucosidase and α-amylase
Table S3: The toxicity prediction of compounds **2** and **3** using ProTox 3.0

.

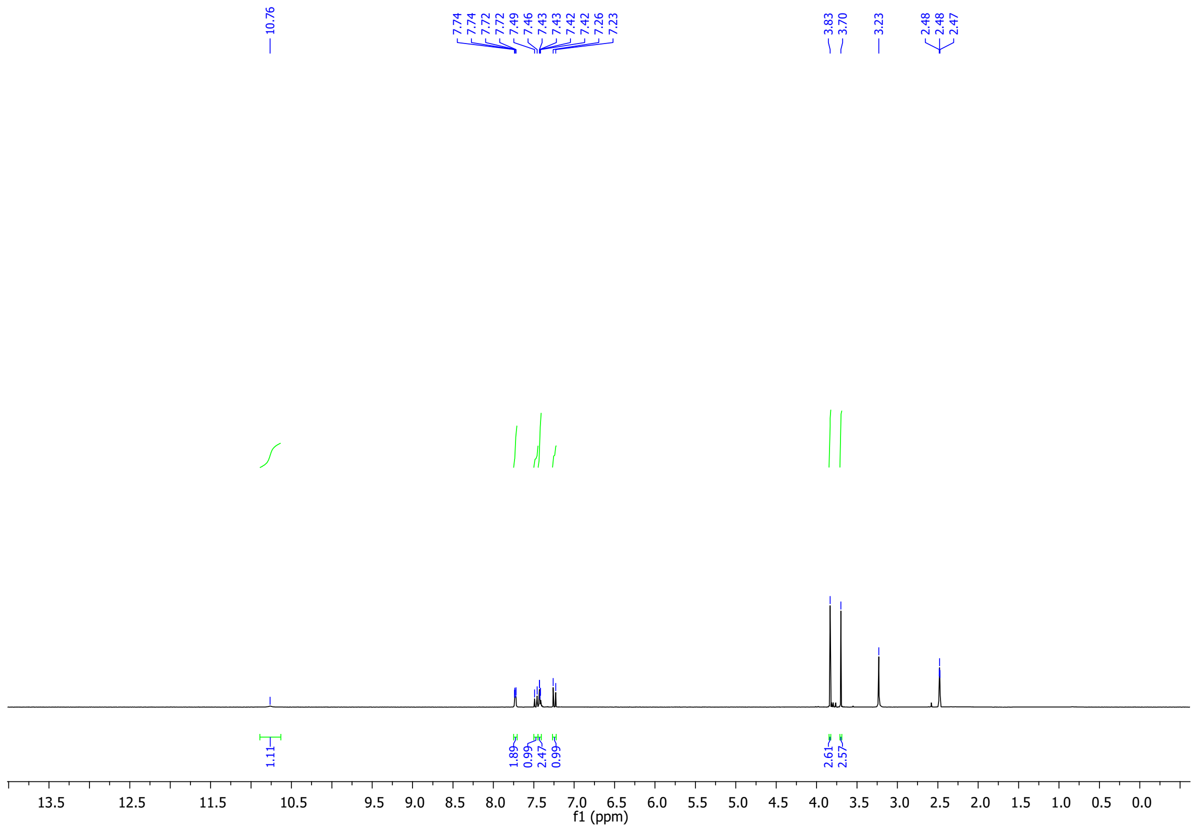
10.76
7.74
7.74
7.72
7.72
7.49
7.46
7.43
7.43
7.42
7.42
7.26
7.23
3.83
3.70
3.23
2.48
2.48
2.47
1.11
1.89
0.99
2.47
0.99
2.61
2.57
13.5
12.5
11.5
10.5
9.5
9.0
8.5
8.0
7.5
7.0
6.5
6.0
5.5
5.0
4.5
4.0
3.5
3.0
2.5
2.0
1.5
1.0
0.5
0.0
f1 (ppm)

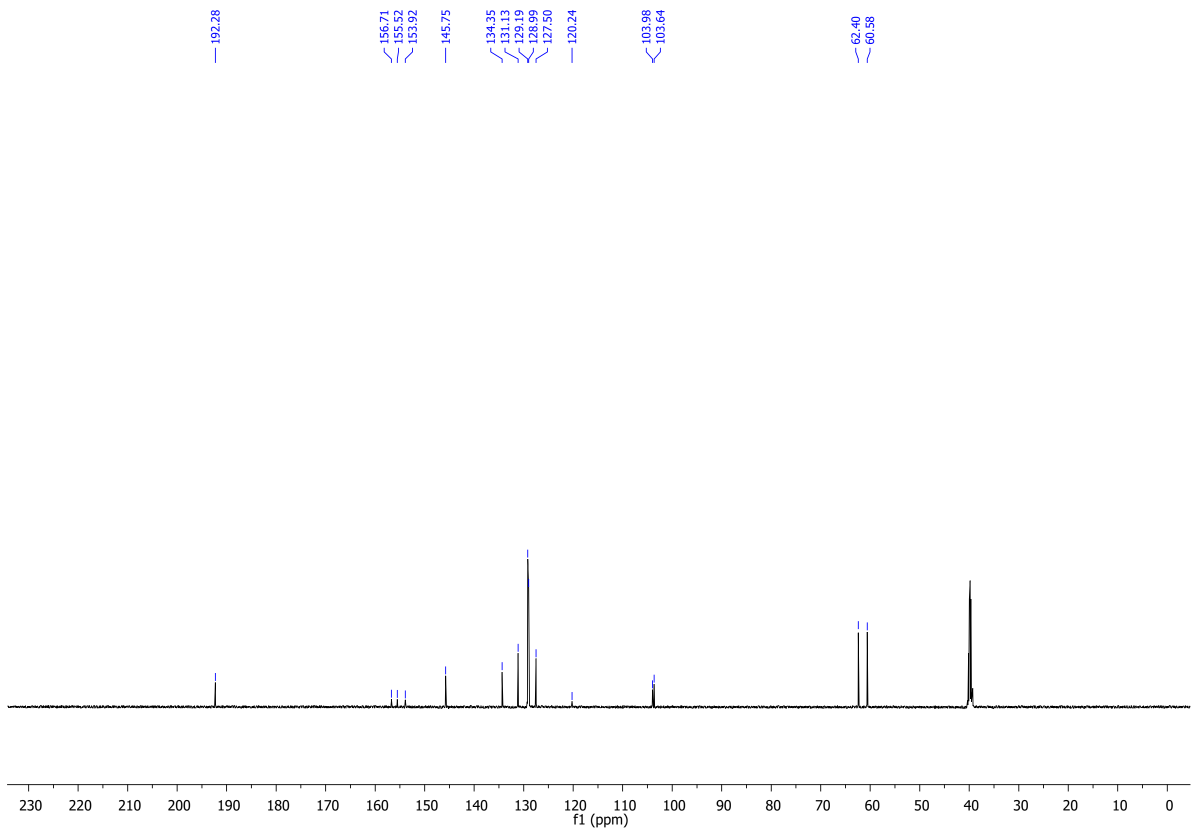
192.28
156.71
155.52
153.92
145.75
134.35
131.13
129.19
128.99
127.50
120.24
103.98
103.64
62.40
60.58
230 220 210 200 190 180 170 160 150 140 130 120 110 100 90 80 70 60 50 40 30 20 10 0
f1 (ppm)

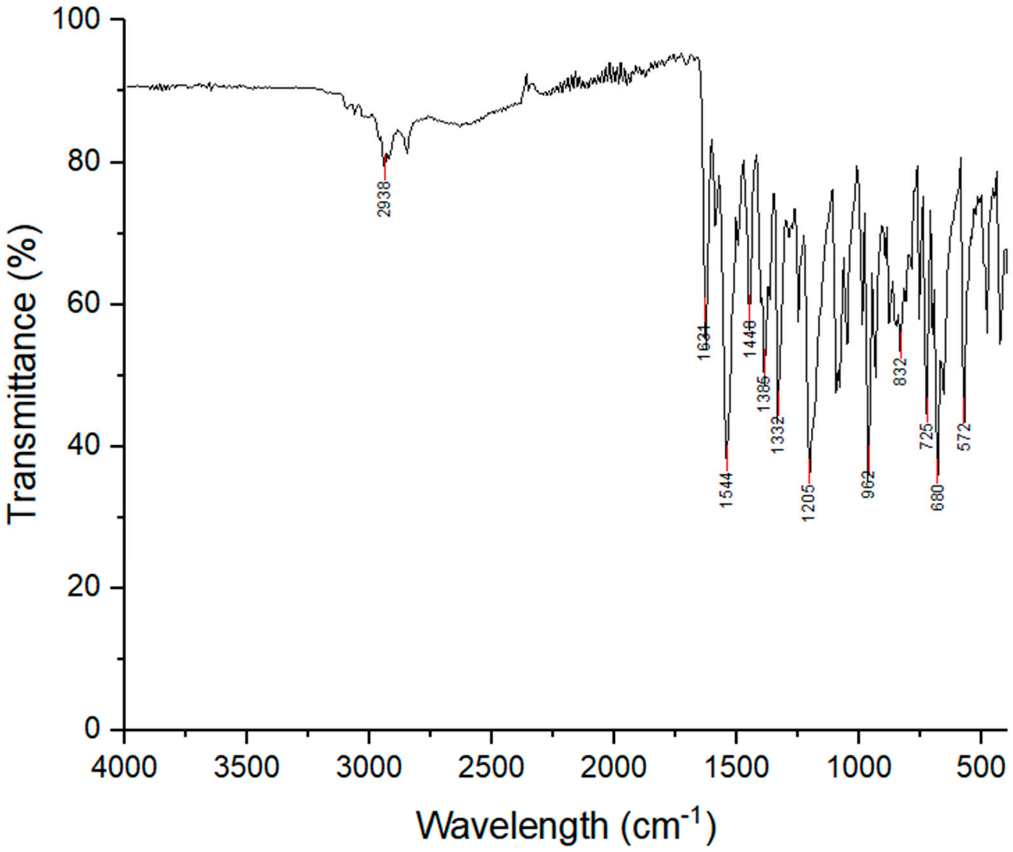

Figure S1.1. NMR (^{1}H- and ^{13}C in DMSO-d_6 at 500 and 125 MHz, respectively) and IR spectra of **2a**, respectively.

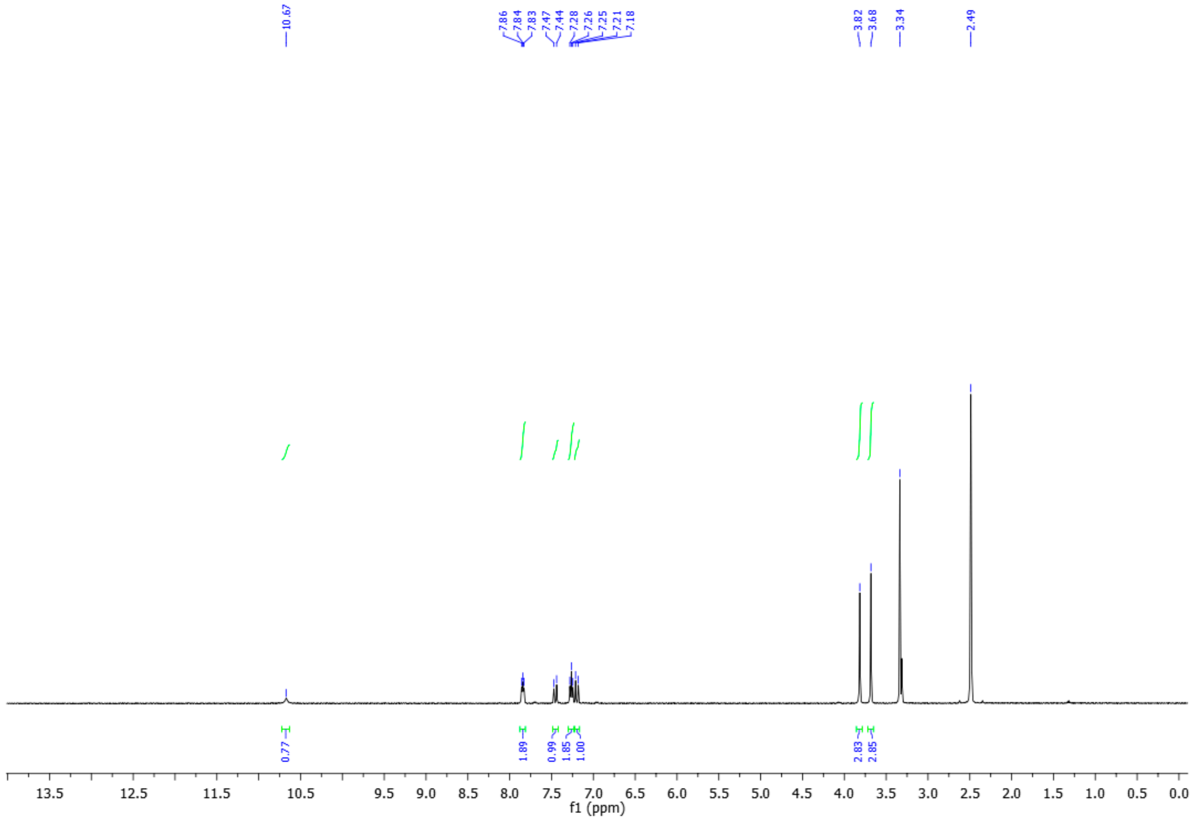
10.67
7.86
7.84
7.83
7.47
7.44
7.28
7.26
7.25
7.21
7.18
3.82
3.68
3.34
2.49
0.77
1.89
0.99
1.85
1.00
2.83
2.85
13.5
12.5
11.5
10.5
9.5
9.0
8.5
8.0
7.5
7.0
6.5
6.0
5.5
5.0
4.5
4.0
3.5
3.0
2.5
2.0
1.5
1.0
0.5
0.0
f1 (ppm)

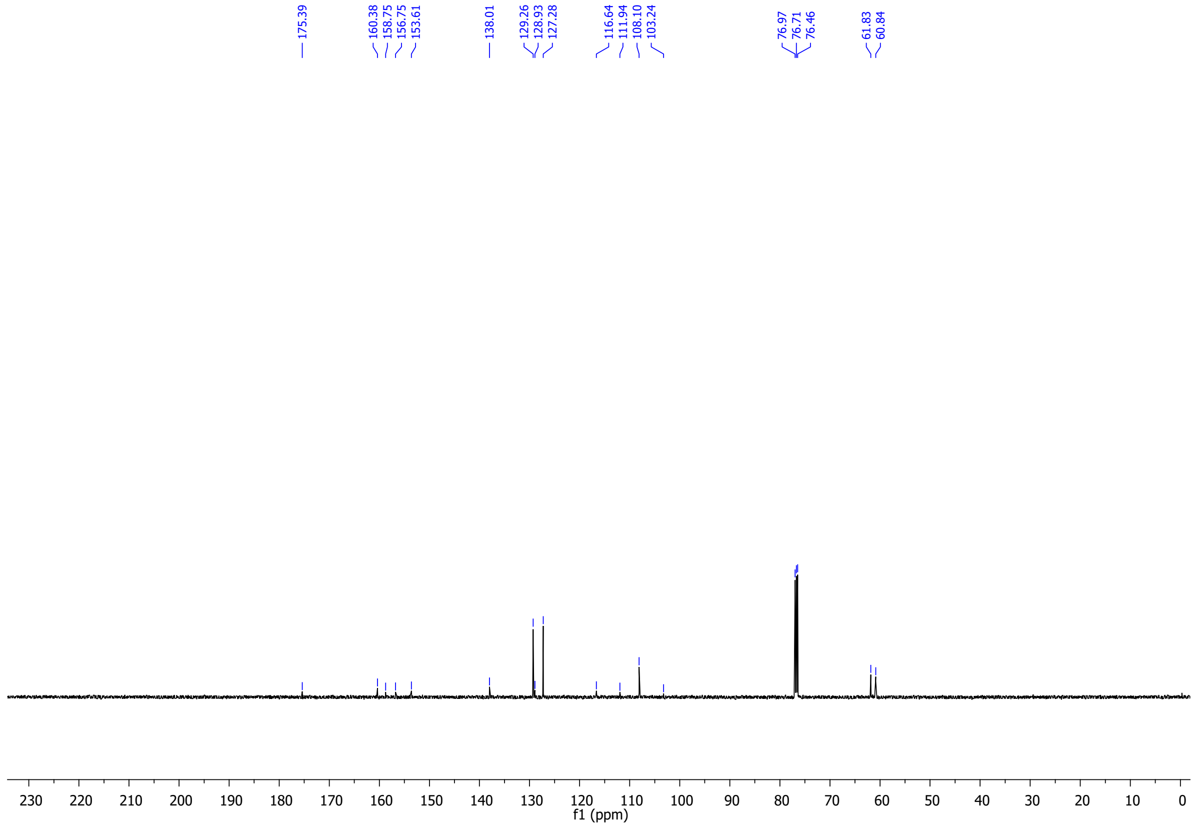
175.39
160.38
158.75
156.75
153.61
138.01
129.26
128.93
127.28
116.64
111.94
108.10
103.24
76.97
76.71
76.46
61.83
60.84
230 220 210 200 190 180 170 160 150 140 130 120 110 100 90 80 70 60 50 40 30 20 10 0
f1 (ppm)

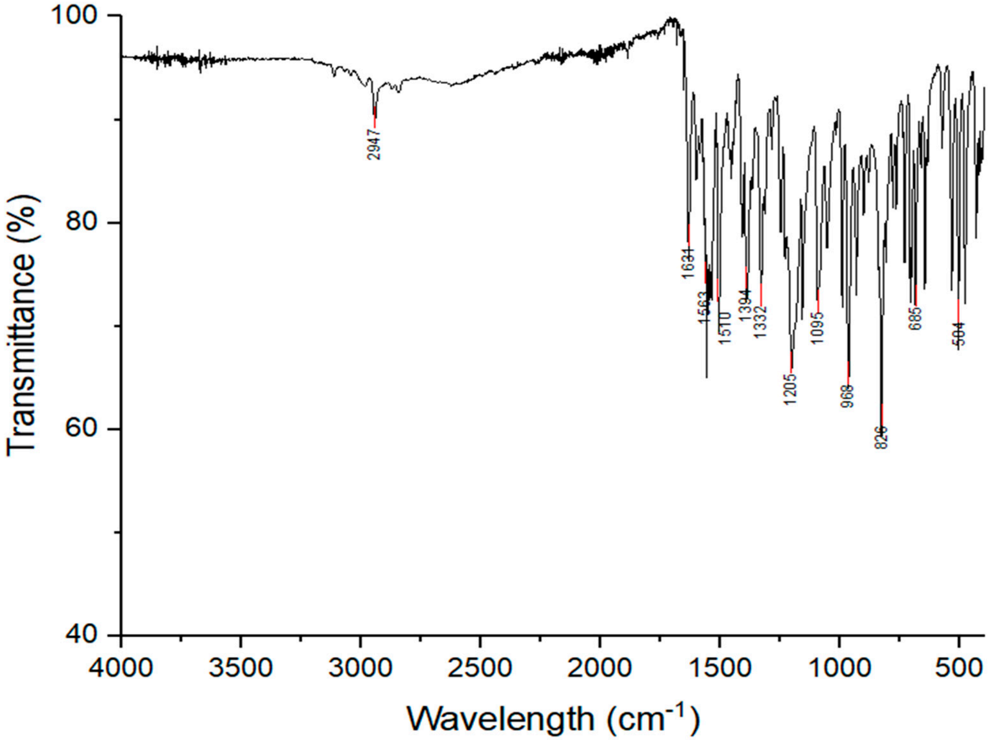

Figure S1.2. NMR (^{1}H- and ^{13}C in DMSO-d_6 at 500 and 125 MHz, respectively) and IR spectra of **2b**, respectively.

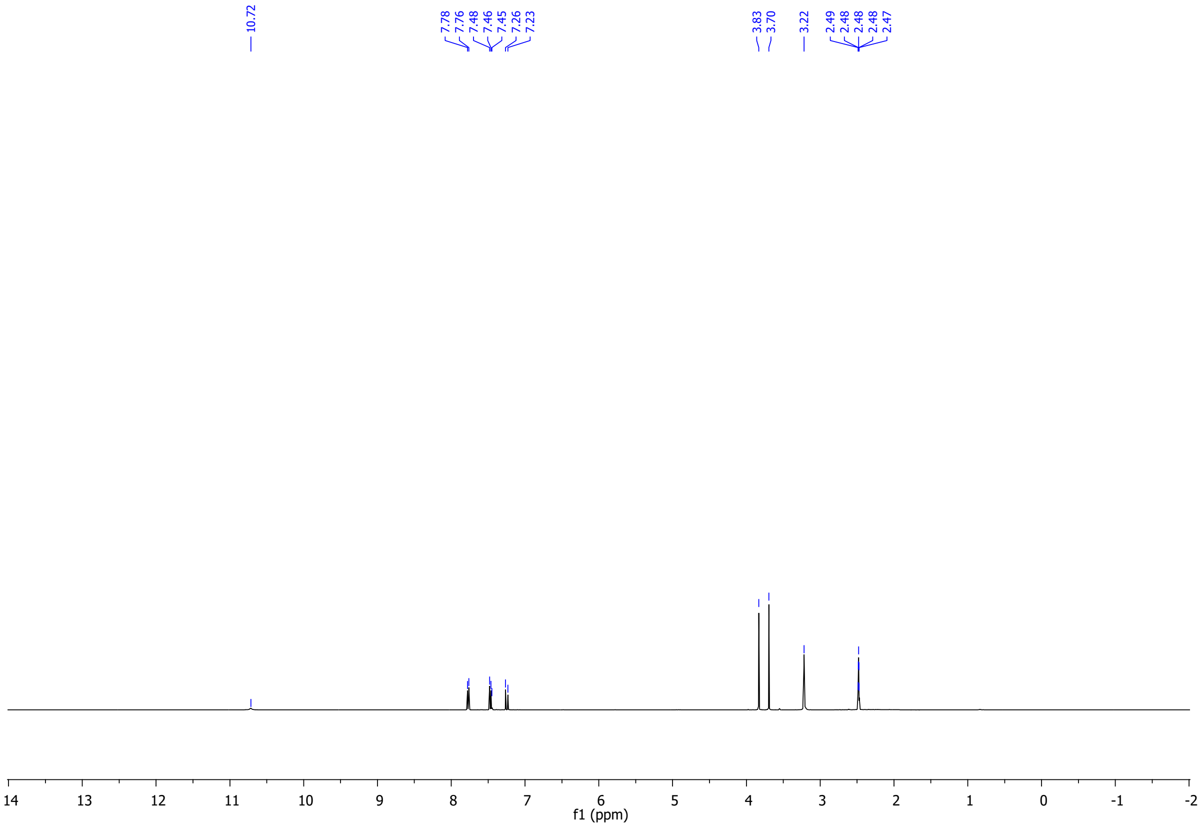
10.72
7.78
7.76
7.48
7.46
7.45
7.26
7.23
3.83
3.70
3.22
2.49
2.48
2.48
2.48
2.47
14 13 12 11 10 9 8 7 6 5 4 3 2 1 0 -1 -2
f1 (ppm)

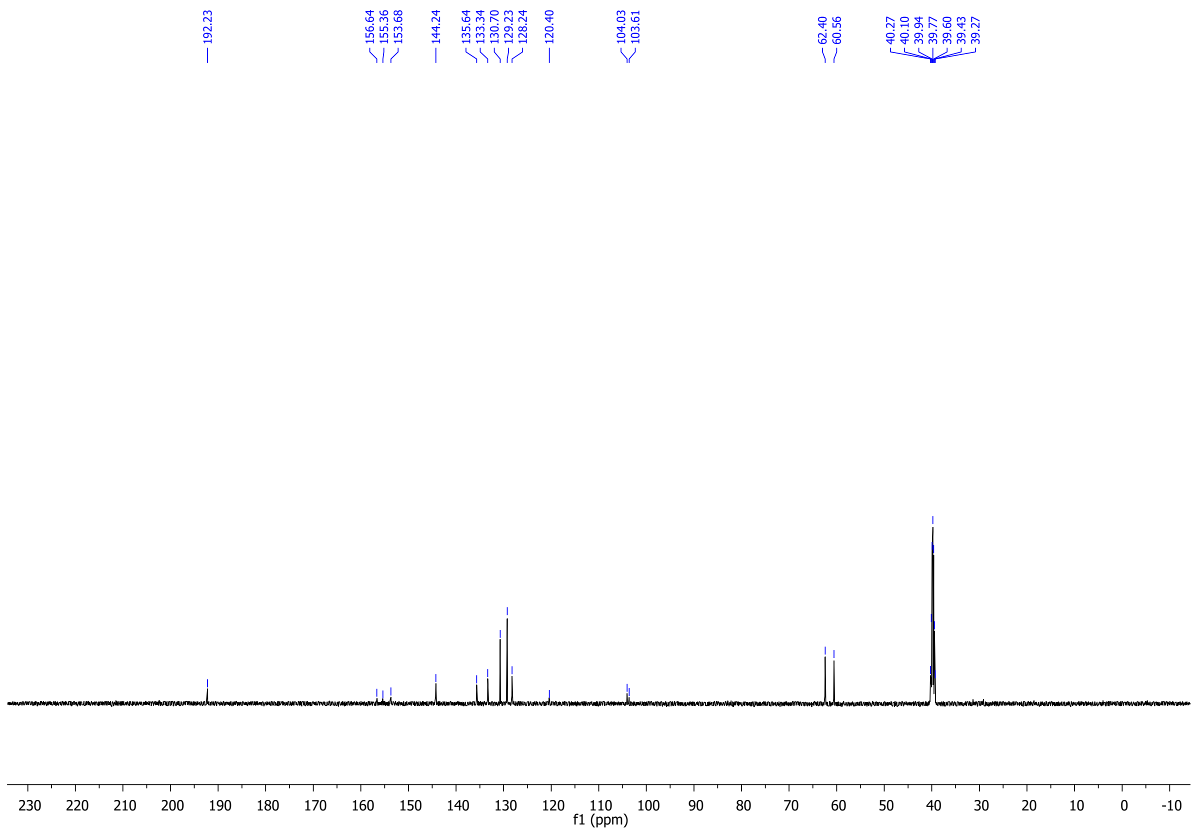
192.23
156.64
155.36
153.68
144.24
135.64
133.34
130.70
129.23
128.24
120.40
104.03
103.61
62.40
60.56
40.27
40.10
39.94
39.77
39.60
39.43
39.27
230 220 210 200 190 180 170 160 150 140 130 120 110 100 90 80 70 60 50 40 30 20 10 0 -10
f1 (ppm)

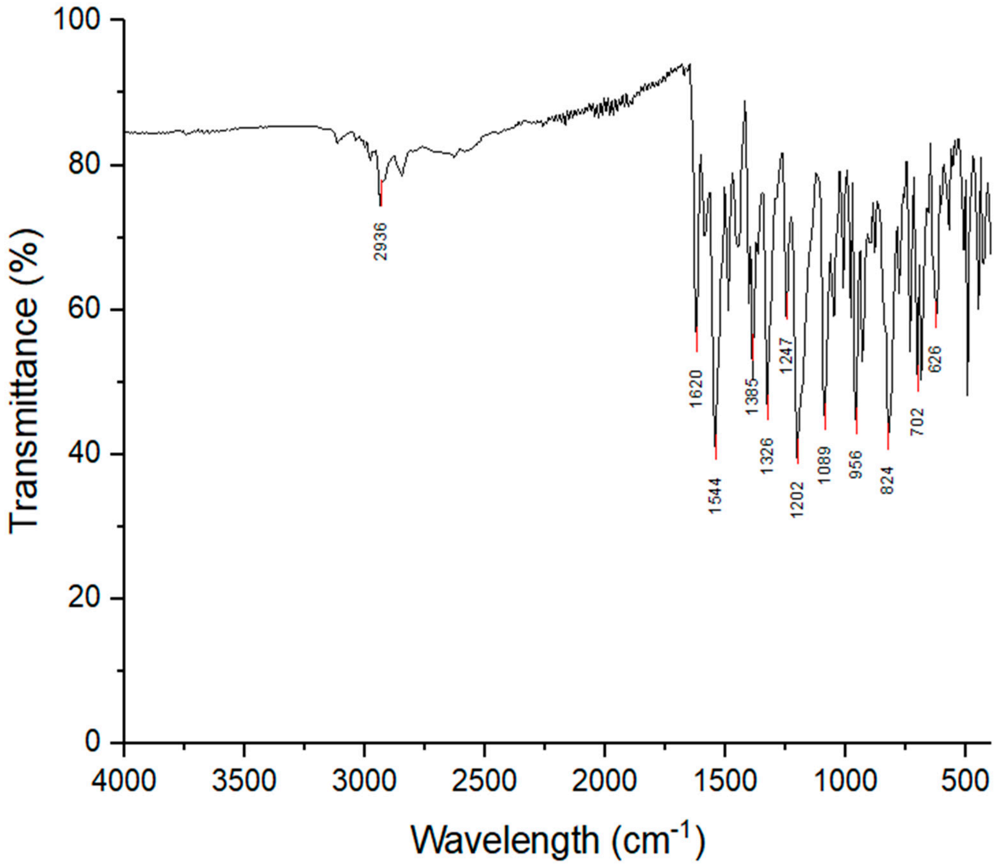

Figure S1.3. NMR (^{1}H- and ^{13}C in DMSO-d_6 at 500 and 125 MHz, respectively) and IR spectra of **2c**, respectively.

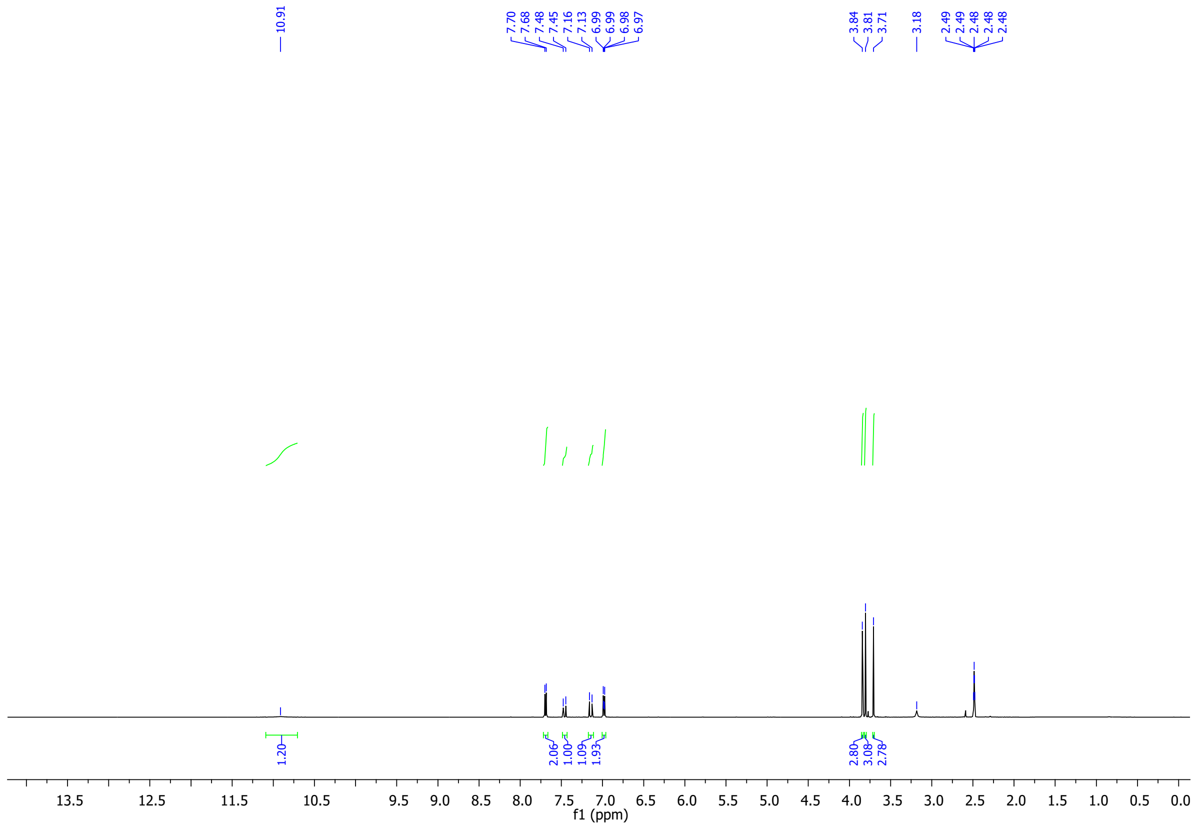
10.91
7.70
7.68
7.48
7.45
7.16
7.13
6.99
6.99
6.98
6.97
3.84
3.81
3.71
3.18
2.49
2.49
2.48
2.48
2.48
1.20
2.06
1.00
1.09
1.93
2.80
3.08
2.78
13.5 12.5 11.5 10.5 9.5 9.0 8.5 8.0 7.5 7.0 6.5 6.0 5.5 5.0 4.5 4.0 3.5 3.0 2.5 2.0 1.5 1.0 0.5 0.0
f1 (ppm)

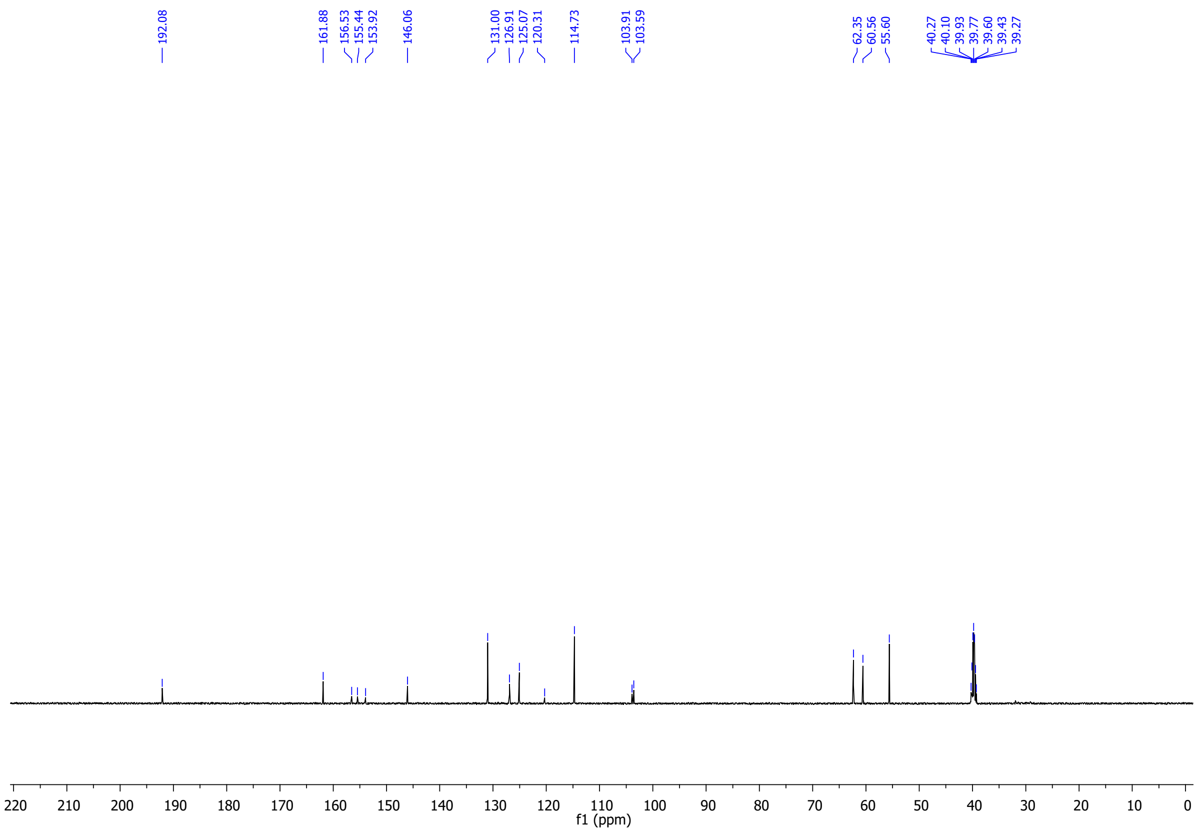
192.08
161.88
156.53
155.44
153.92
146.06
131.00
126.91
125.07
120.31
114.73
103.91
103.59
62.35
60.56
55.60
40.27
40.10
39.93
39.77
39.60
39.43
39.27
220 210 200 190 180 170 160 150 140 130 120 110 100 90 80 70 60 50 40 30 20 10 0
f1 (ppm)

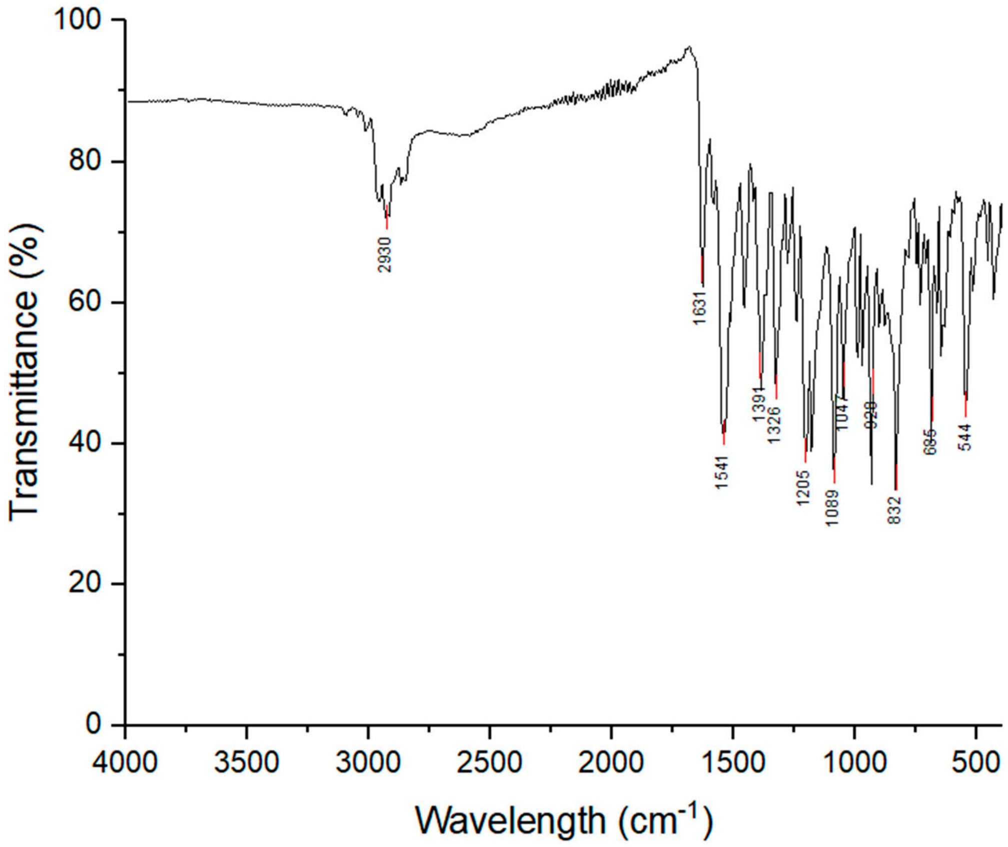

Figure S1.4. NMR (^{1}H- and ^{13}C in DMSO-d_6 at 500 and 125 MHz, respectively) and IR spectra of **2d**, respectively.

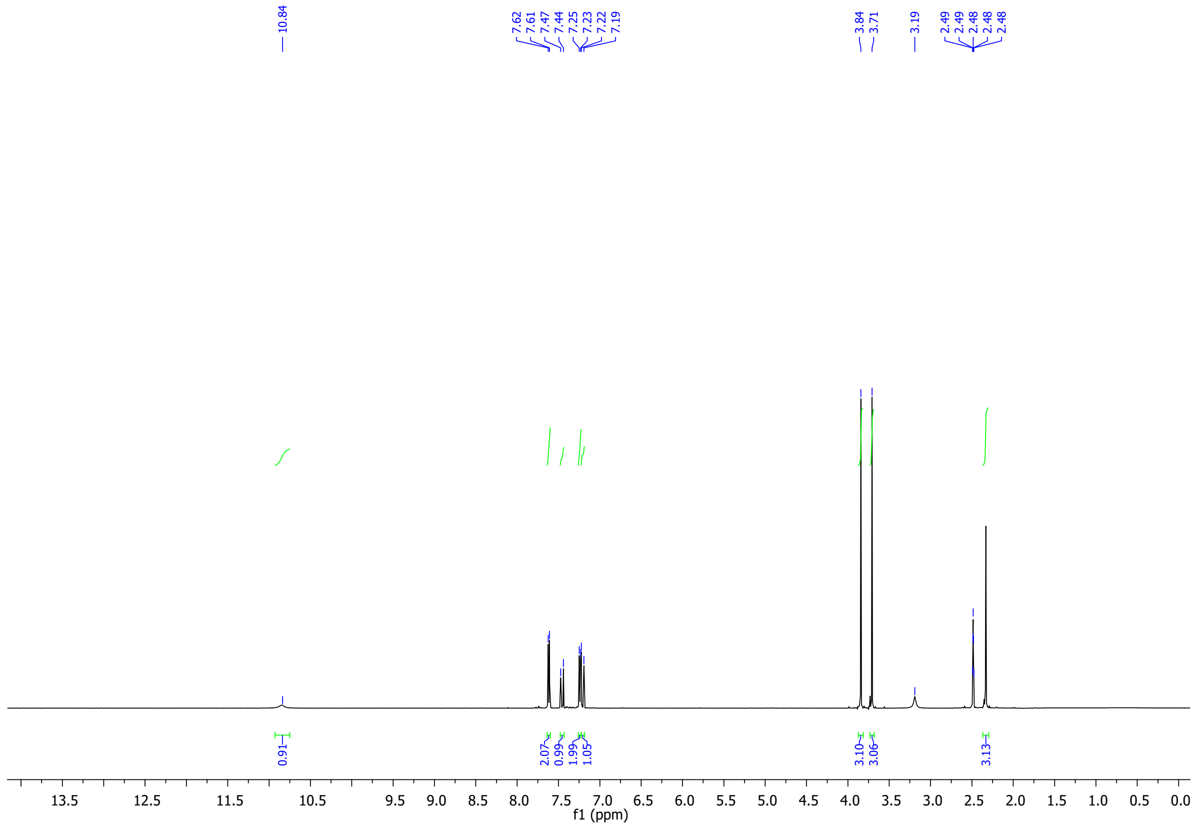
10.84
7.62
7.61
7.47
7.44
7.25
7.23
7.22
7.19
3.84
3.71
3.19
2.49
2.49
2.48
2.48
2.48
0.91
2.07
0.99
1.99
1.05
3.10
3.06
3.13
13.5
12.5
11.5
10.5
9.5
9.0
8.5
8.0
7.5
7.0
6.5
6.0
5.5
5.0
4.5
4.0
3.5
3.0
2.5
2.0
1.5
1.0
0.5
0.0
f1 (ppm)

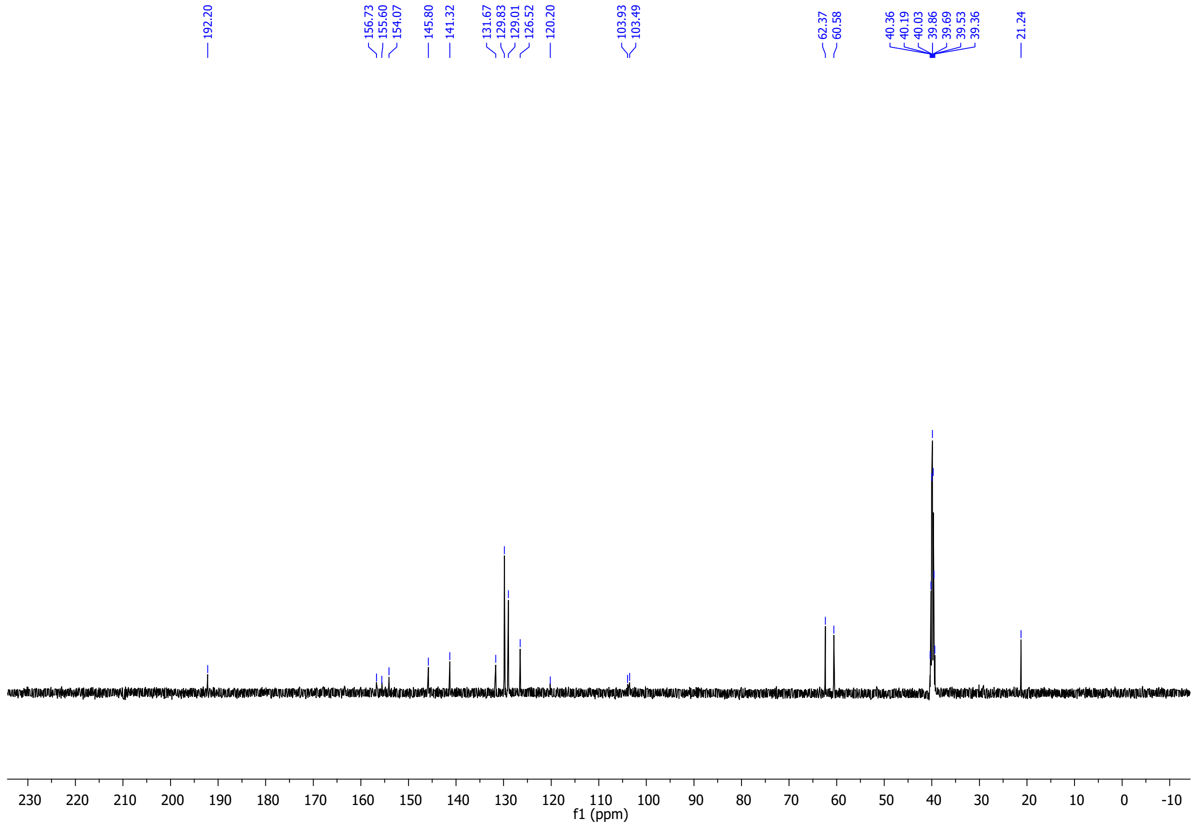
192.20
156.73
155.60
154.07
145.80
141.32
131.67
129.83
129.01
126.52
120.20
103.93
103.49
62.37
60.58
40.36
40.19
40.03
39.86
39.69
39.53
39.36
21.24
230 220 210 200 190 180 170 160 150 140 130 120 110 100 90 80 70 60 50 40 30 20 10 0 -10
f1 (ppm)

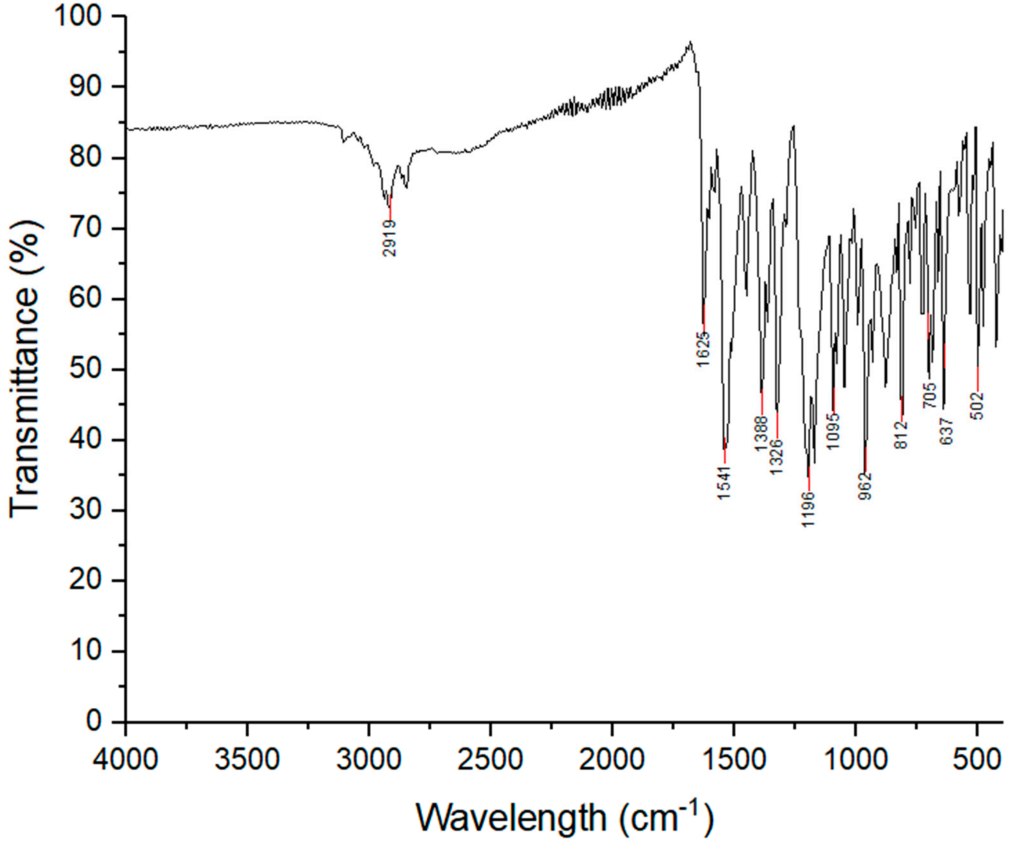

Figure S1.5. NMR (^{1}H- and ^{13}C NMR in DMSO-d_6 at 500 and 125 MHz, respectively) and IR spectra **2e**, respectively.

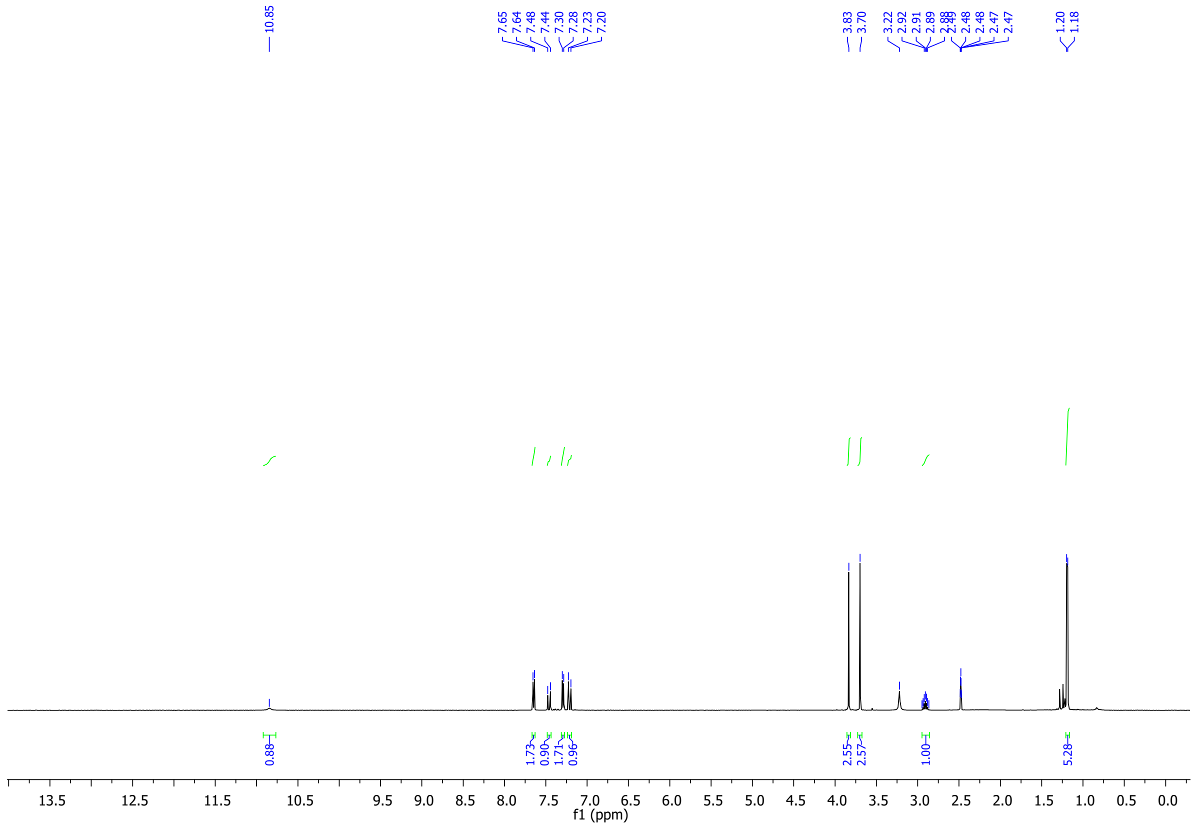

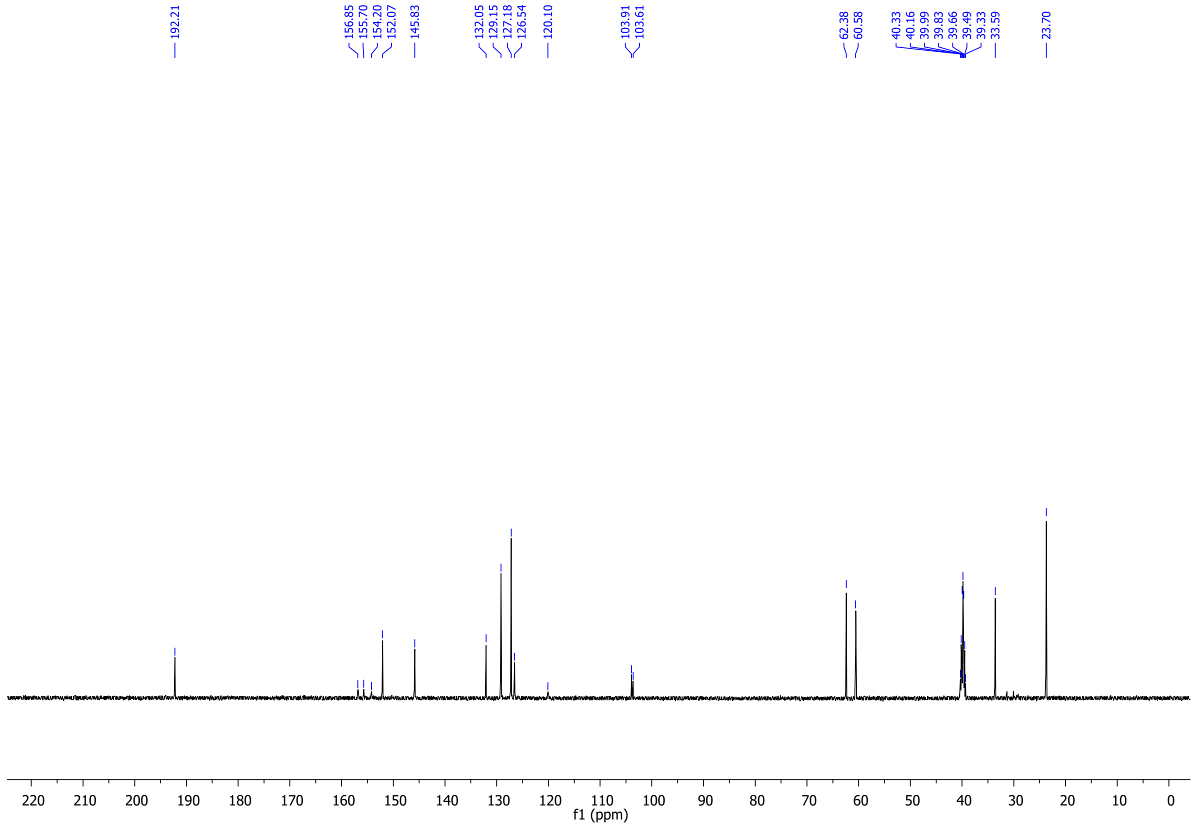
192.21
156.85
155.70
154.20
152.07
145.83
132.05
129.15
127.18
126.54
120.10
103.91
103.61
62.38
60.58
40.33
40.16
39.99
39.83
39.66
39.49
39.33
33.59
23.70
220 210 200 190 180 170 160 150 140 130 120 110 100 90 80 70 60 50 40 30 20 10 0
f1 (ppm)

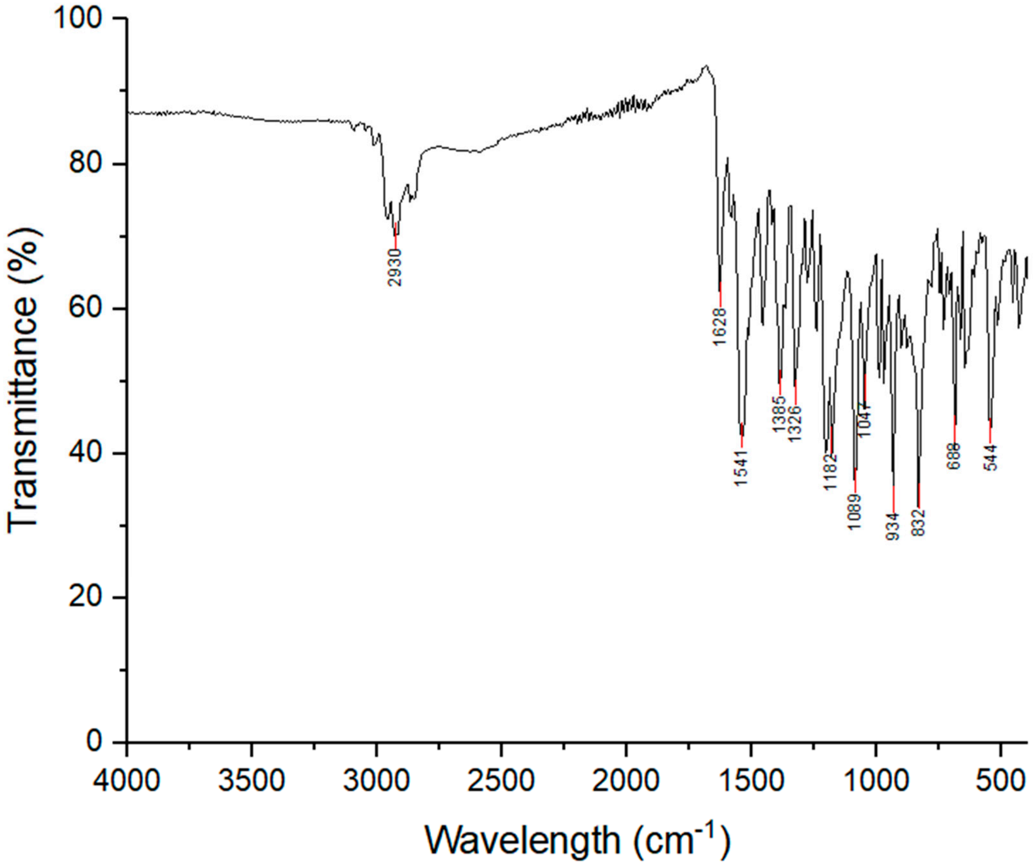

Figure S1.6. NMR (^{1}H- and ^{13}C in DMSO-d_6 at 500 and 125 MHz, respectively) and IR spectra of **2f**, respectively.

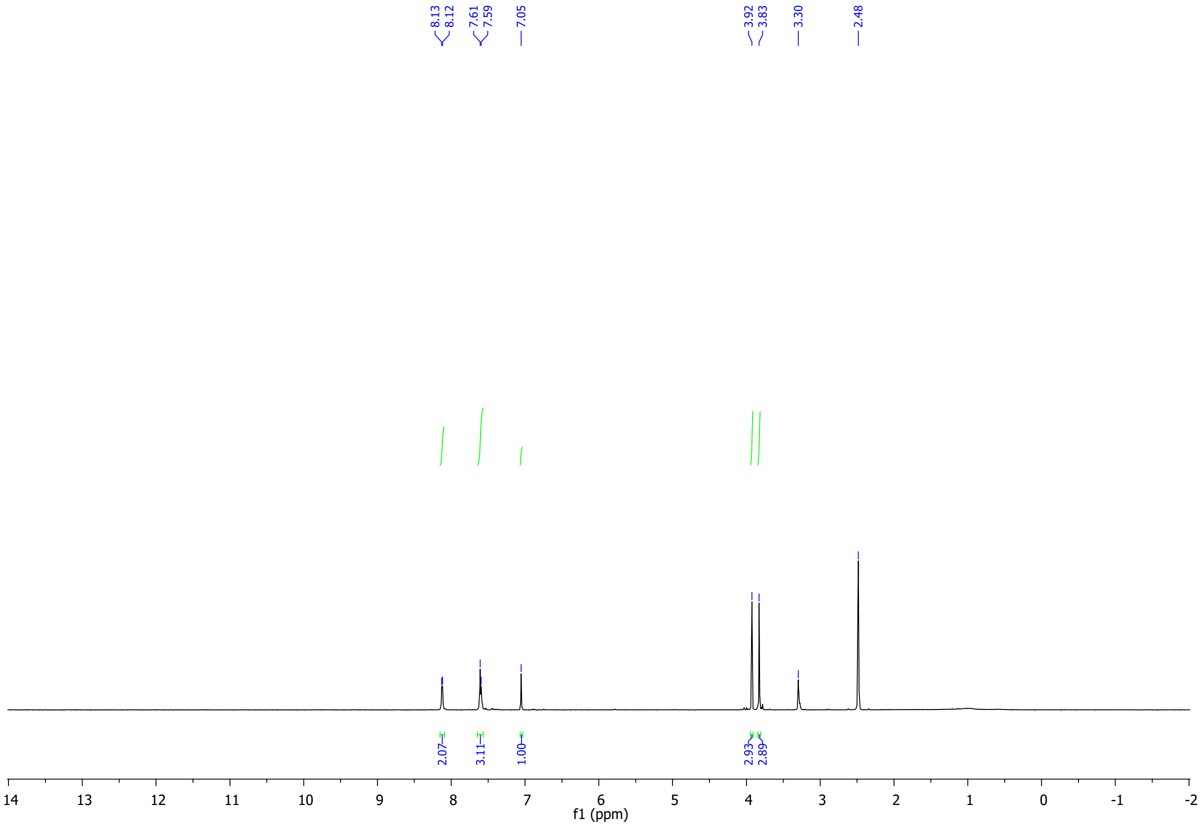
8.13
8.12
7.61
7.59
7.05
3.92
3.83
3.30
2.48
2.07
3.11
1.00
2.93
2.89
14 13 12 11 10 9 8 7 6 5 4 3 2 1 0 -1 -2
f1 (ppm)

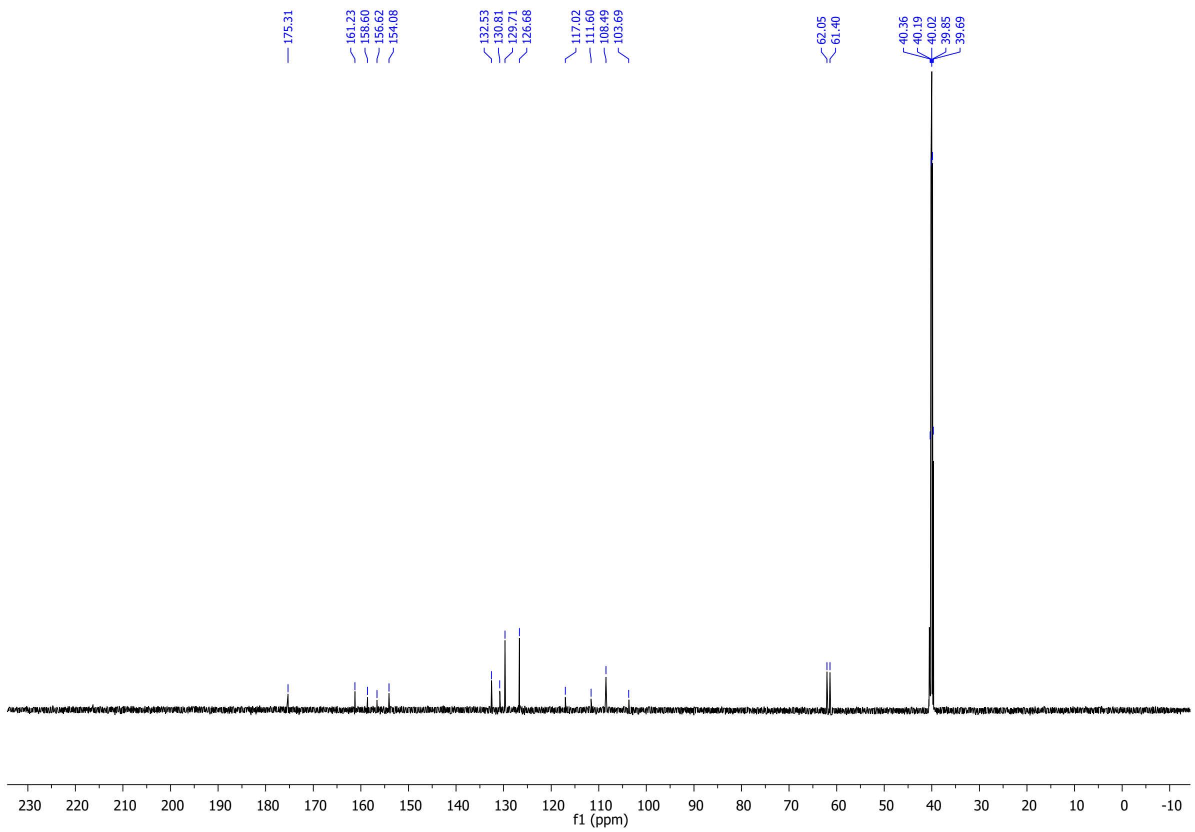
175.31
161.23
158.60
156.62
154.08
132.53
130.81
129.71
126.68
117.02
111.60
108.49
103.69
62.05
61.40
40.36
40.19
40.02
39.85
39.69
230 220 210 200 190 180 170 160 150 140 130 120 110 100 90 80 70 60 50 40 30 20 10 0 -10
f1 (ppm)

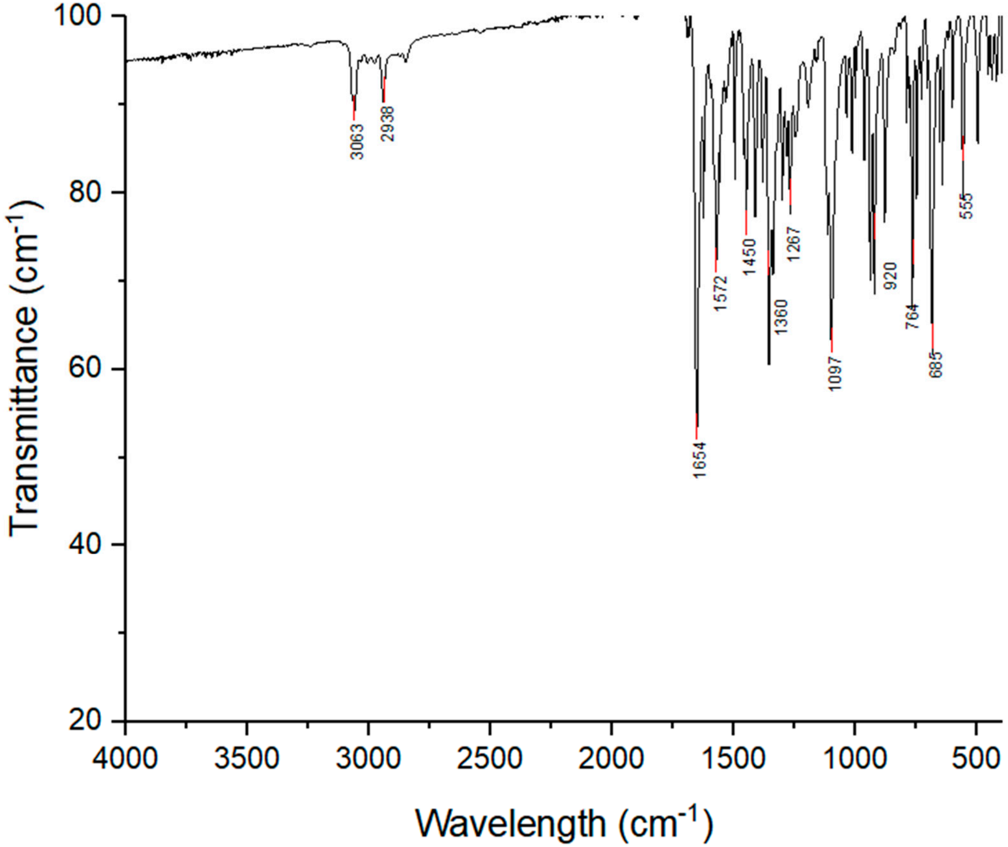

Figure S1.8. NMR (^{1}H- and ^{13}C in DMSO-d_6 at 500 and 125 MHz, respectively) and IR spectra of **3a**, respectively.

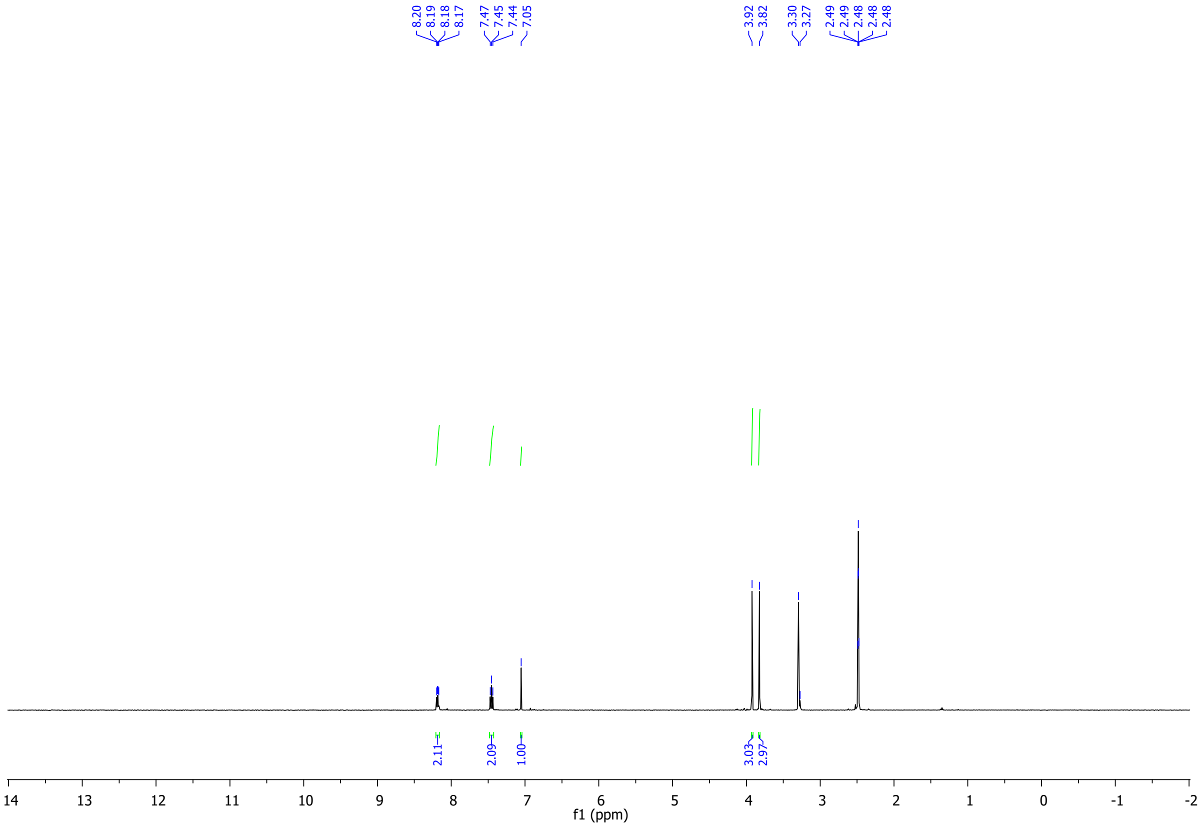
8.20
8.19
8.18
8.17
7.47
7.45
7.44
7.05
3.92
3.82
3.30
3.27
2.49
2.49
2.48
2.48
2.48
2.11
2.09
1.00
3.03
2.97
14
13
12
11
10
9
8
7
6
5
4
3
2
1
0
-1
-2
f1 (ppm)

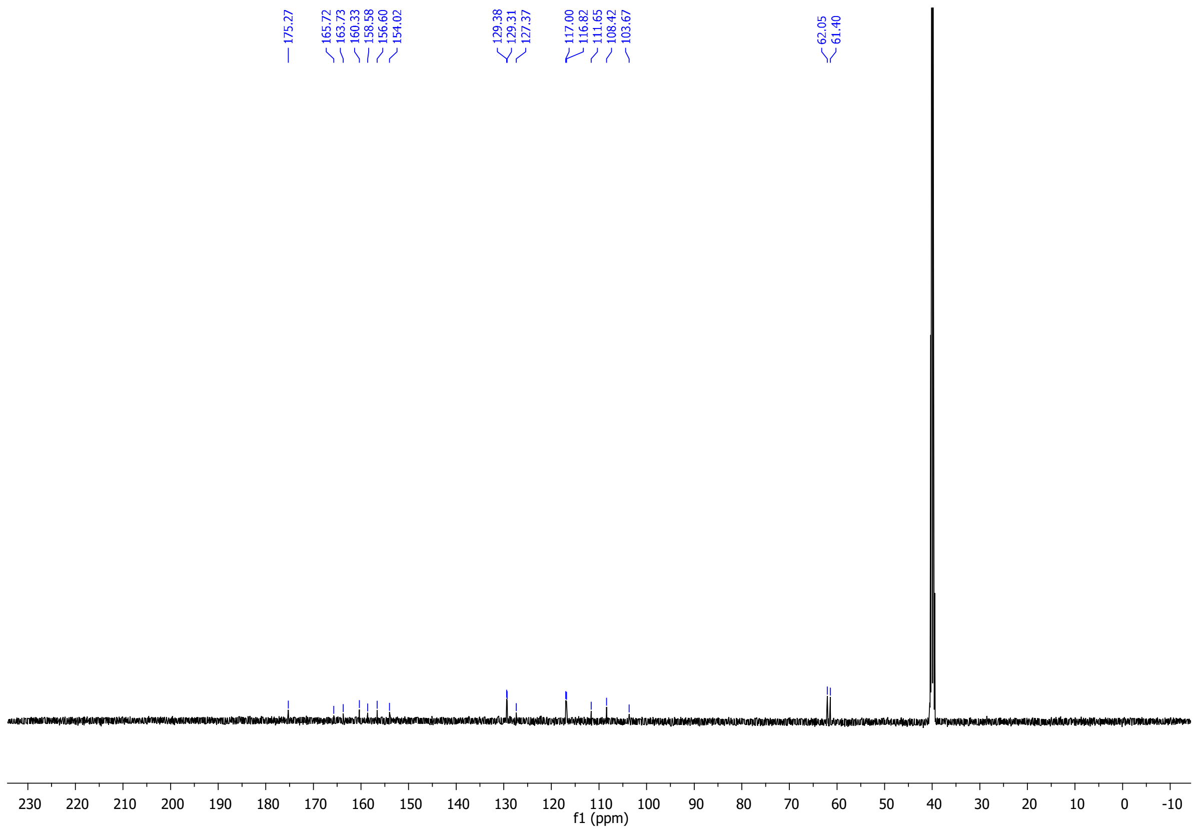
175.27
165.72
163.73
160.33
158.58
156.60
154.02
129.38
129.31
127.37
117.00
116.82
111.65
108.42
103.67
62.05
61.40
230 220 210 200 190 180 170 160 150 140 130 120 110 100 90 80 70 60 50 40 30 20 10 0 -10
f1 (ppm)

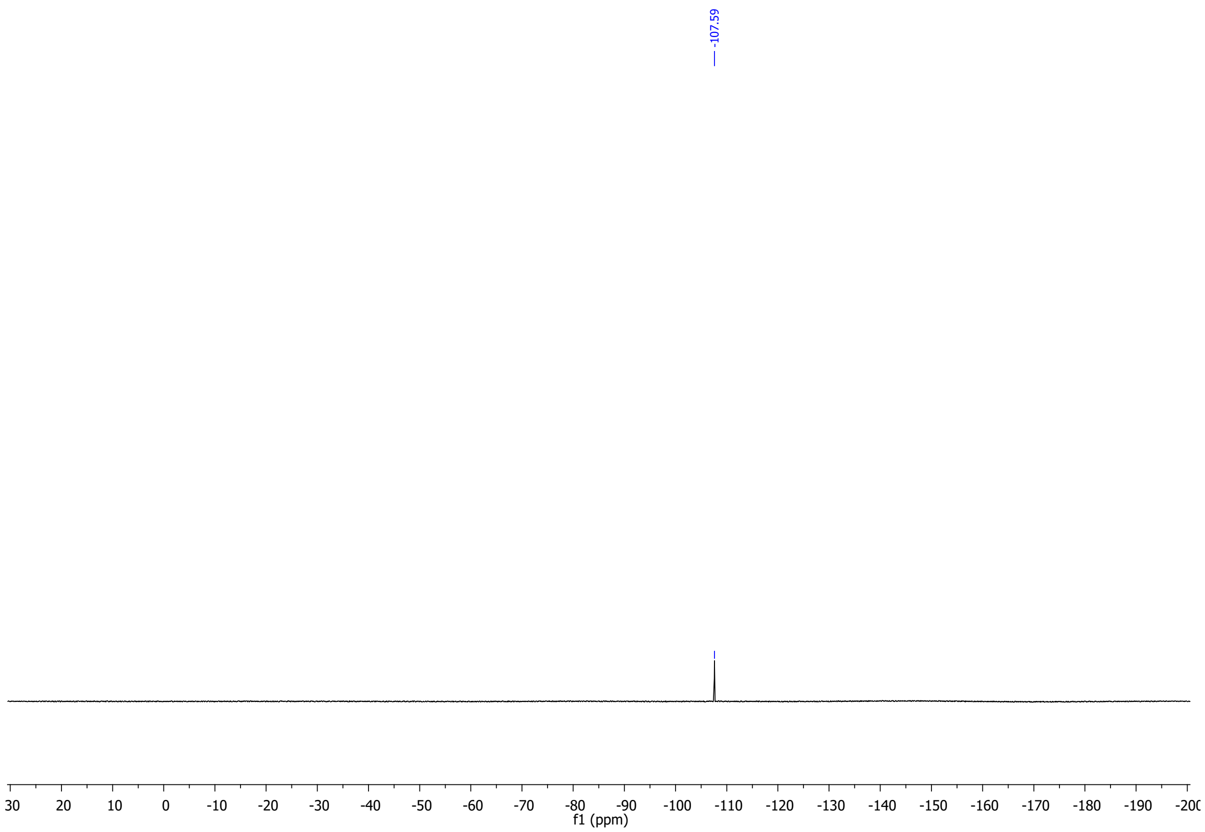

-107.59
30
20
10
0
-10
-20
-30
-40
-50
-60
-70
-80
-90
-100
-110
-120
-130
-140
-150
-160
-170
-180
-190
-200
f1 (ppm)

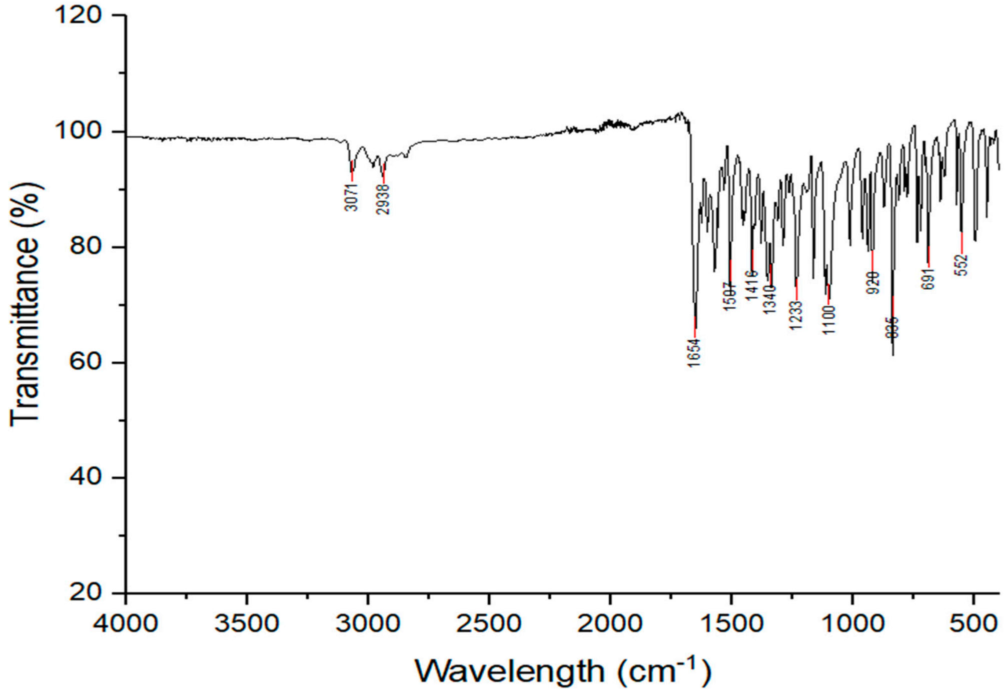

Figure S1.9. NMR (^{1}H- and ^{13}C in DMSO-d_6 at 500 and 125 MHz, respectively) and IR spectra of **3b**, respectively.

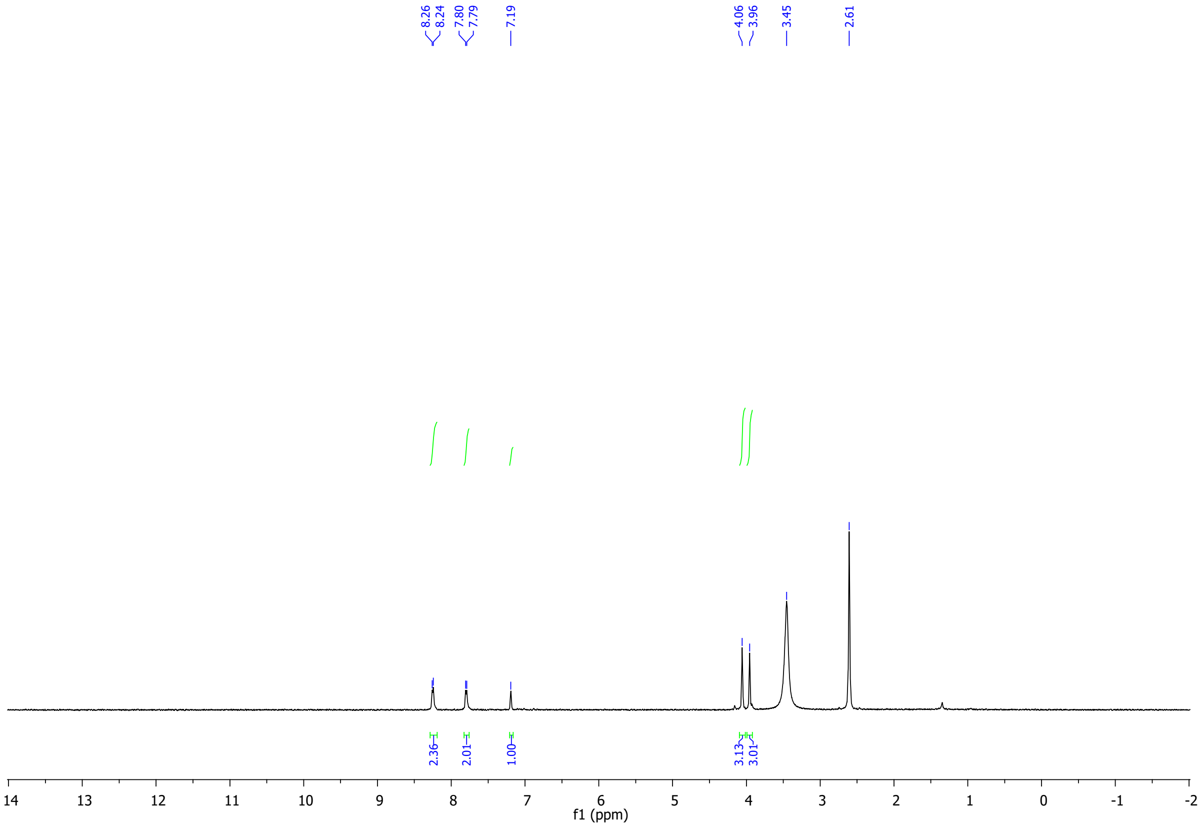
8.26
8.24
7.80
7.79
7.19
4.06
3.96
3.45
2.61
2.36
2.01
1.00
3.13
3.01
14 13 12 11 10 9 8 7 6 5 4 3 2 1 0 -1 -2
f1 (ppm)

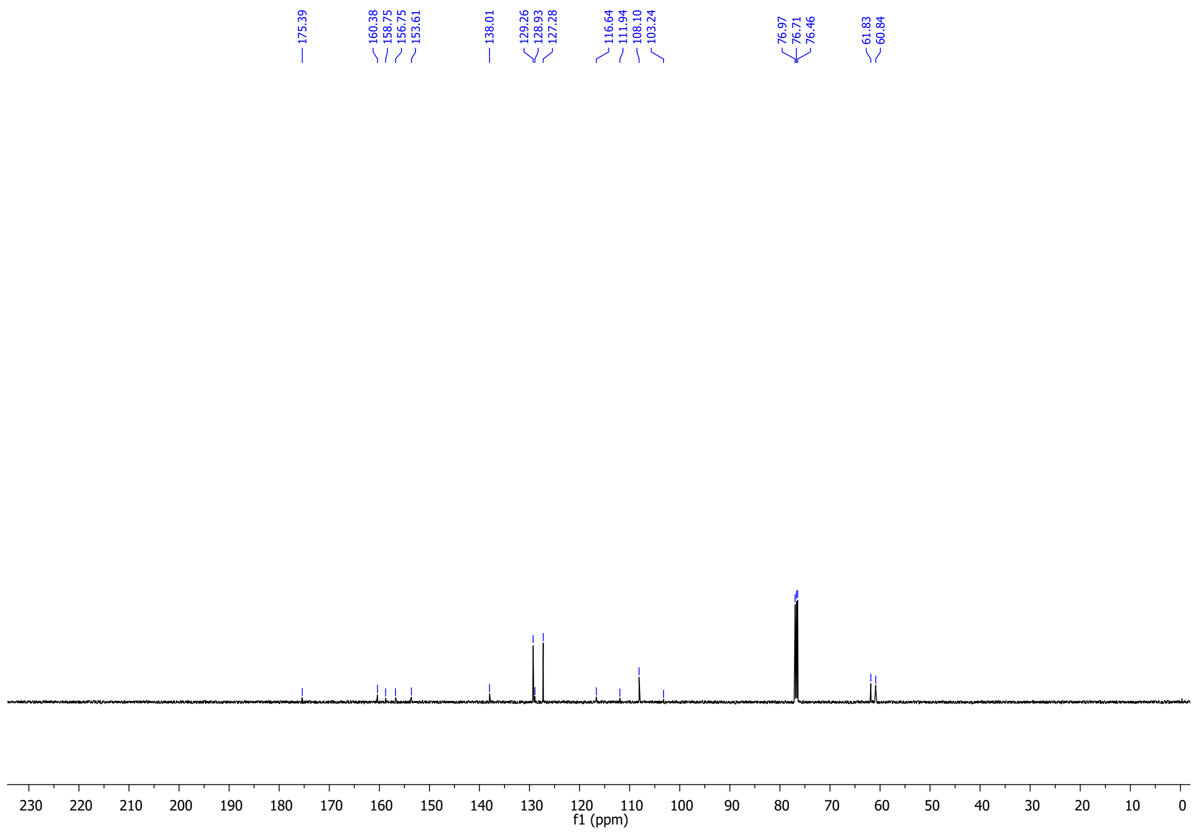
175.39
160.38
158.75
156.75
153.61
138.01
129.26
128.93
127.28
116.64
111.94
108.10
103.24
76.97
76.71
76.46
61.83
60.84
230 220 210 200 190 180 170 160 150 140 130 120 110 100 90 80 70 60 50 40 30 20 10 0
f1 (ppm)

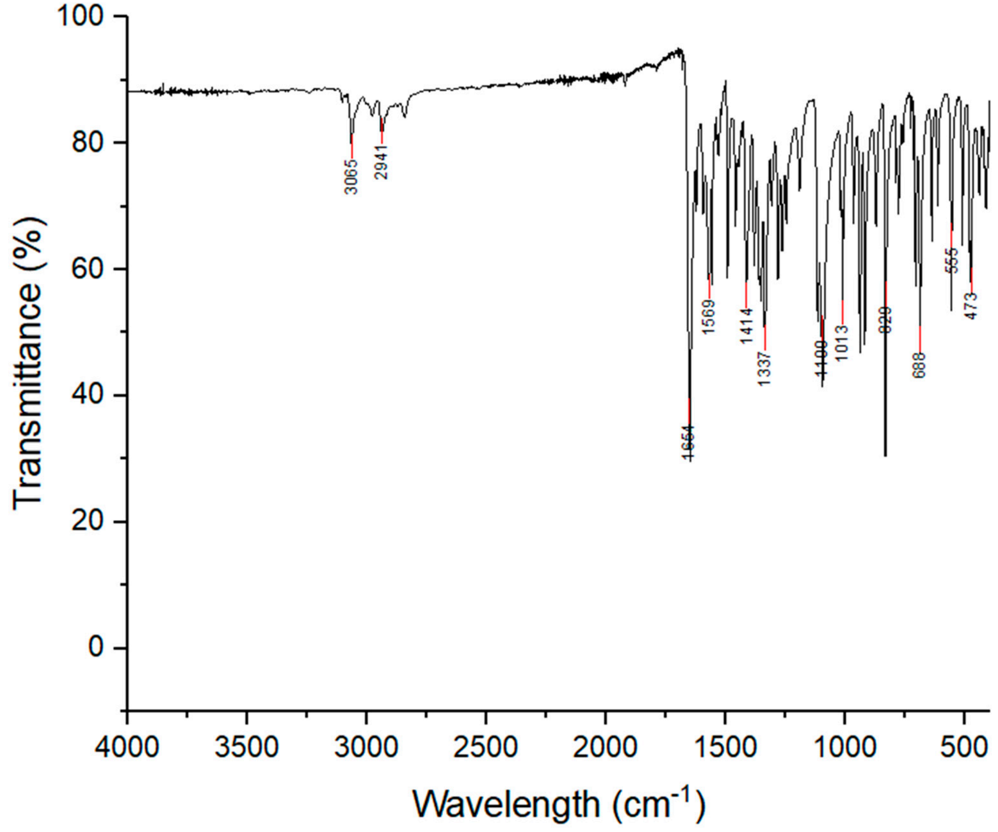

Figure S1.9. NMR (^{1}H- and ^{13}C in DMSO-d_6 at 500 and 125 MHz, respectively) and IR spectra of **3c**, respectively.

.

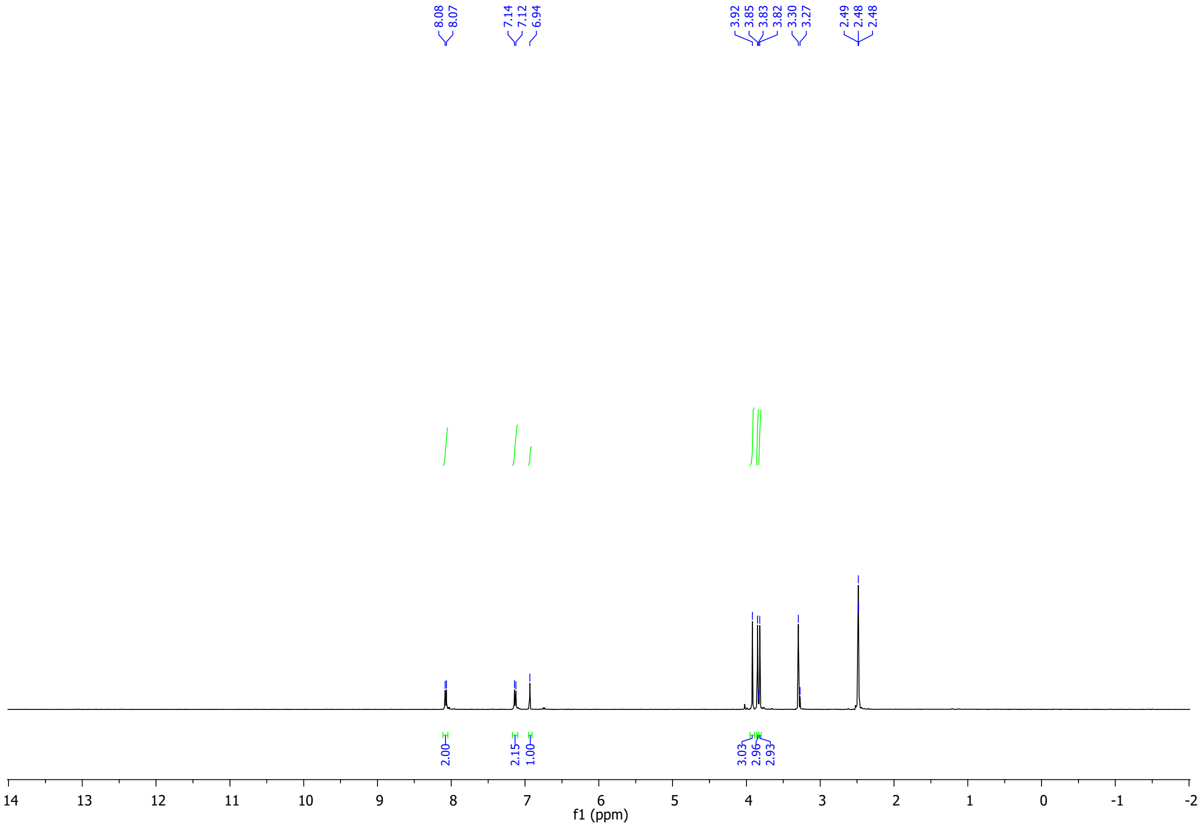
8.08
8.07
7.14
7.12
6.94
3.92
3.85
3.83
3.82
3.30
3.27
2.49
2.48
2.48
2.00
2.15
1.00
3.03
2.96
2.93
14 13 12 11 10 9 8 7 6 5 4 3 2 1 0 -1 -2
f1 (ppm)

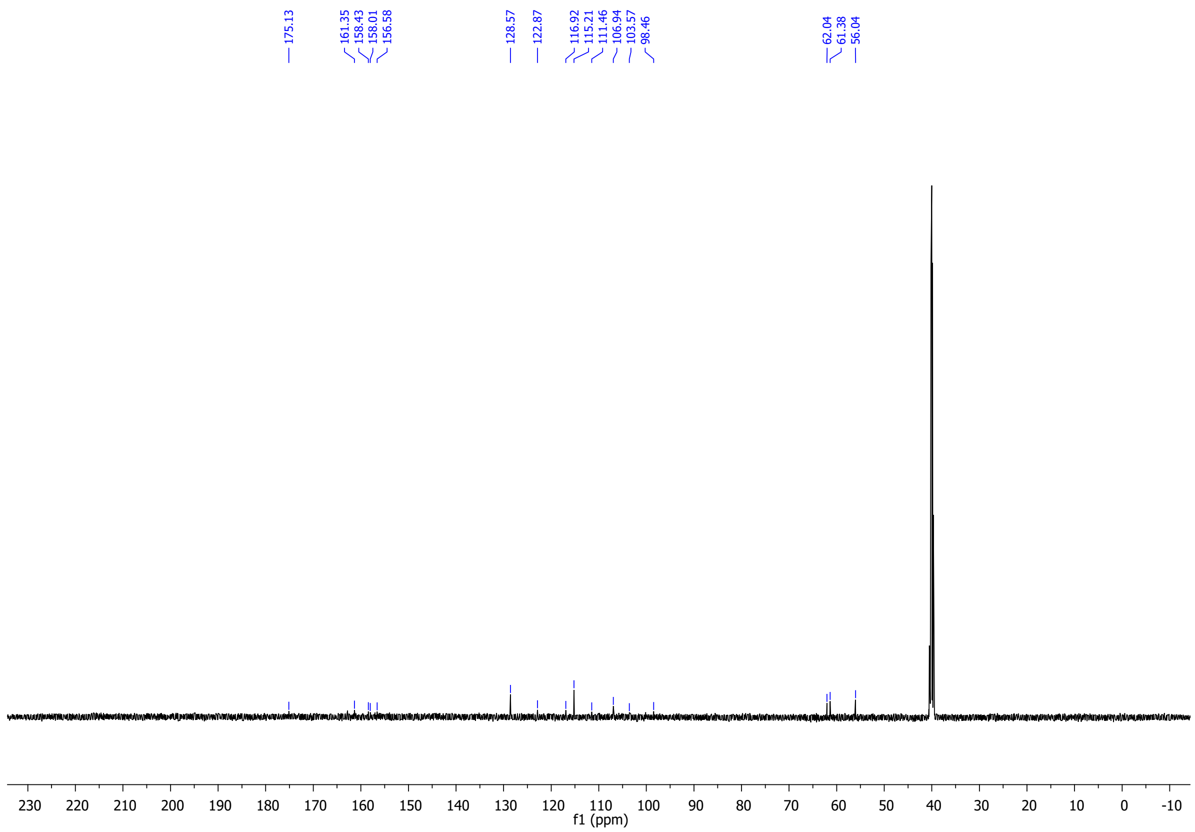
175.13
161.35
158.43
158.01
156.58
128.57
122.87
116.92
115.21
111.46
106.94
103.57
98.46
62.04
61.38
56.04
230
220
210
200
190
180
170
160
150
140
130
120
110
100
90
80
70
60
50
40
30
20
10
0
-10
f1 (ppm)

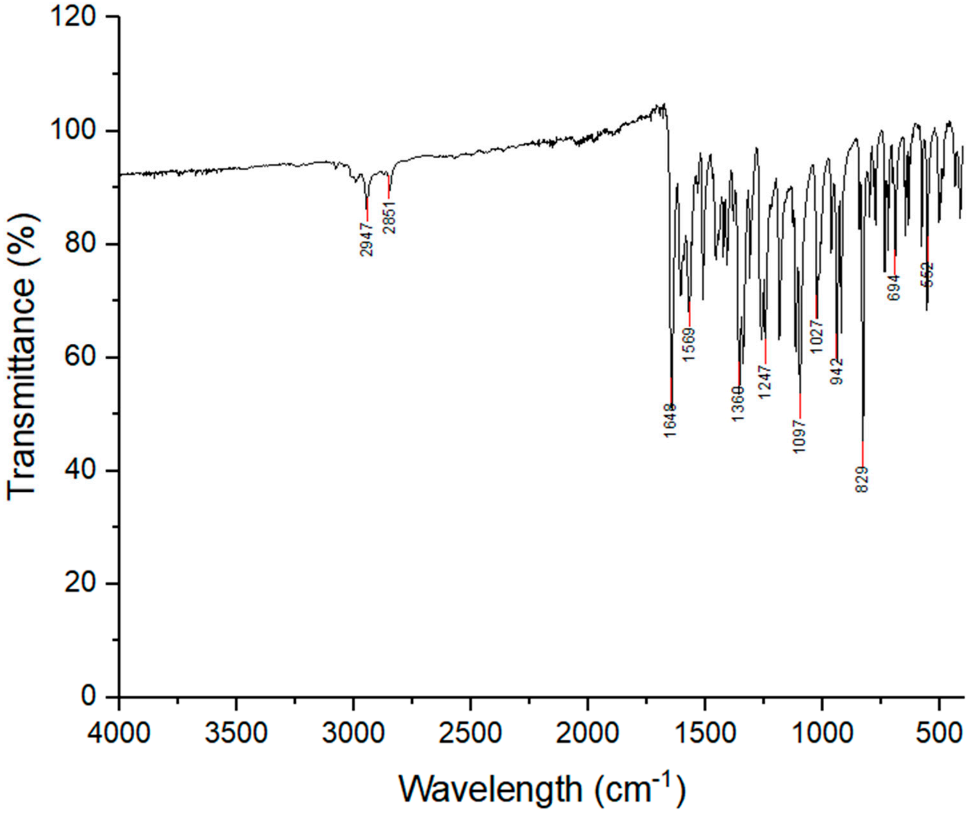

Figure S1.10. NMR (^{1}H- and ^{13}C in DMSO-d_6 at 500 and 125 MHz, respectively) and IR spectra of **3d**, respectively.

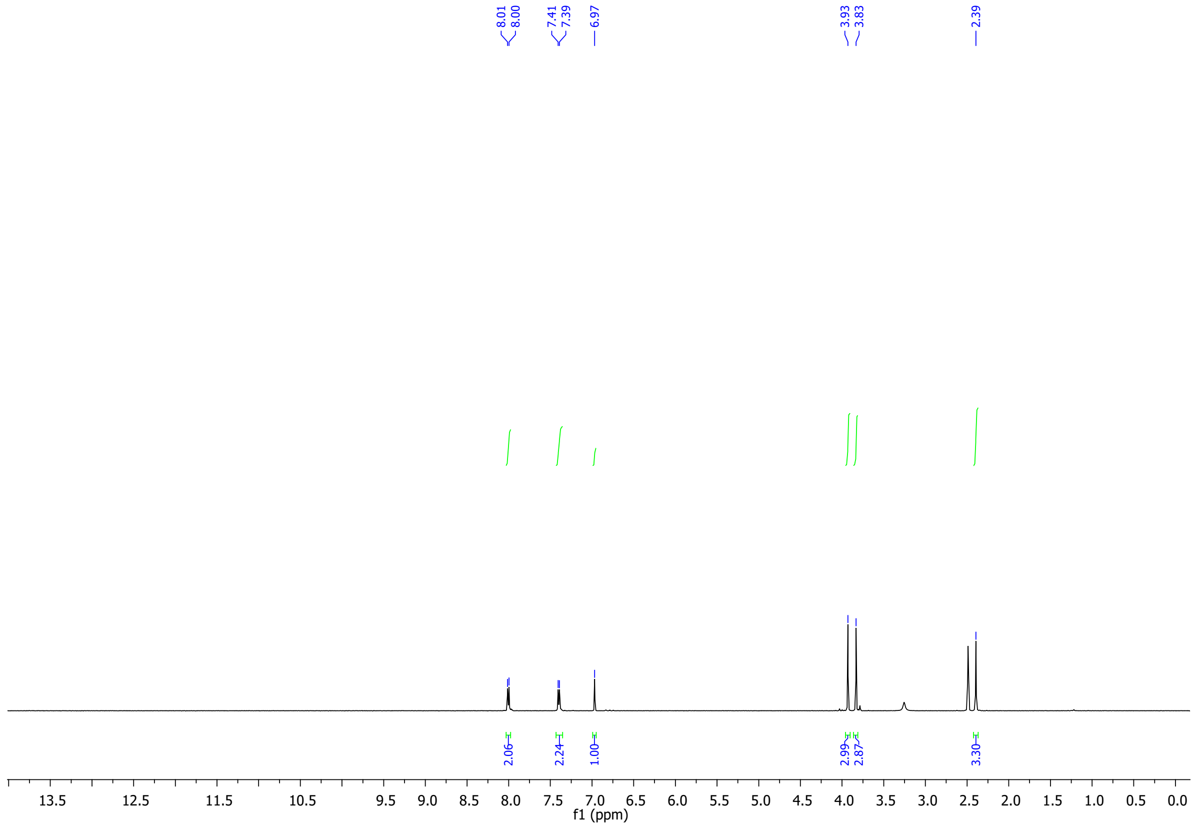
8.01
8.00
7.41
7.39
6.97
3.93
3.83
2.39
2.06
2.24
1.00
2.99
2.87
3.30
13.5
12.5
11.5
10.5
9.5
9.0
8.5
8.0
7.5
7.0
6.5
6.0
5.5
5.0
4.5
4.0
3.5
3.0
2.5
2.0
1.5
1.0
0.5
0.0
f1 (ppm)

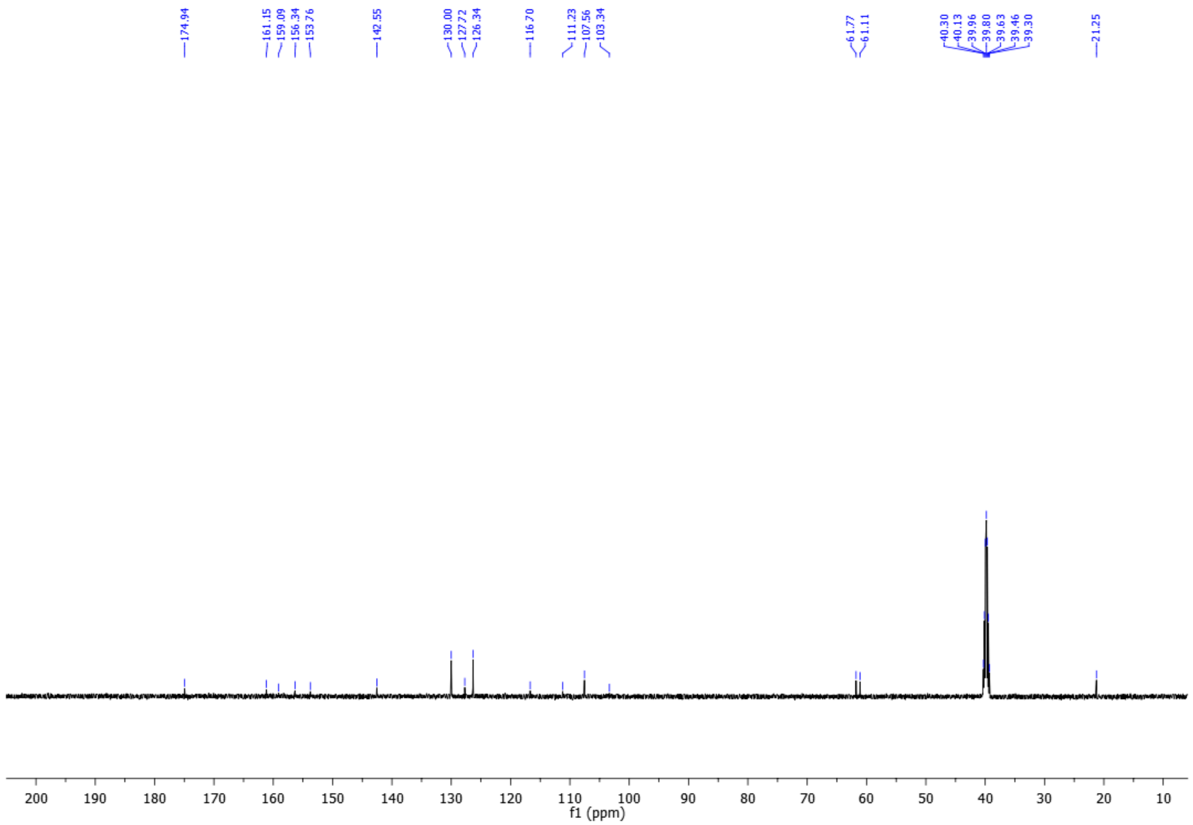
174.94
161.15
159.09
156.34
153.76
142.55
130.00
127.72
126.34
116.70
111.23
107.56
103.34
61.77
61.11
40.30
40.13
39.96
39.80
39.63
39.46
39.30
21.25
200 190 180 170 160 150 140 130 120 110 100 90 80 70 60 50 40 30 20 10
f1 (ppm)

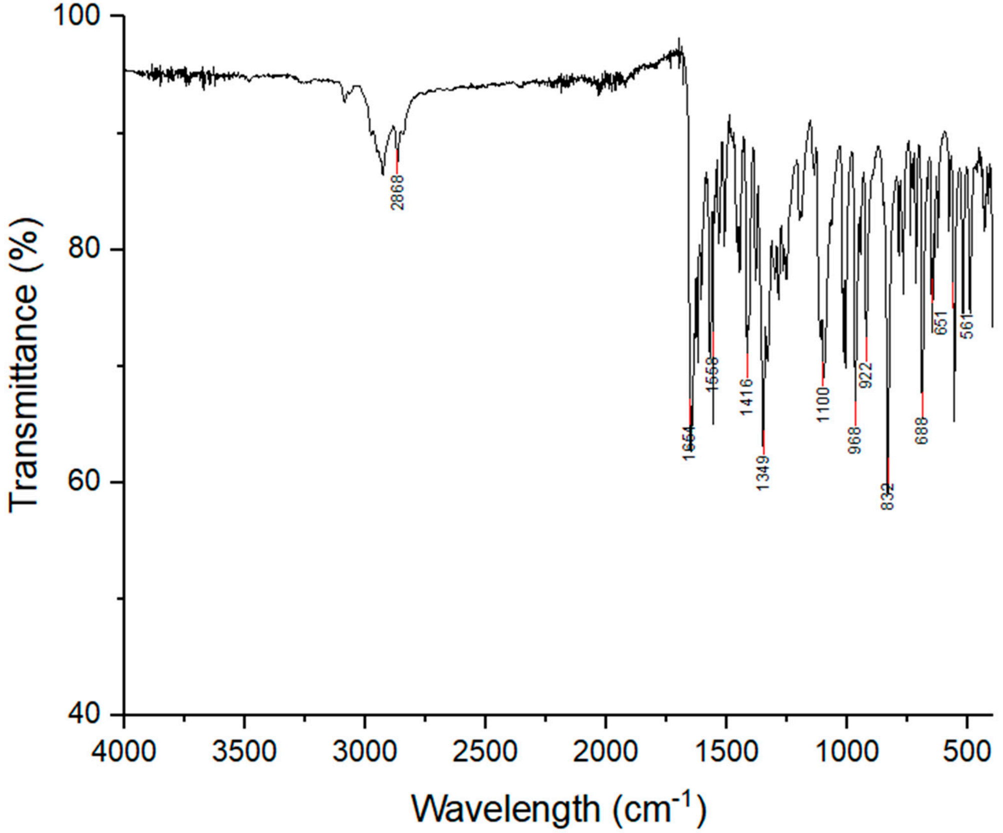

Figure S1.11. NMR (^{1}H- and ^{13}C in DMSO-d_6 at 500 and 125 MHz, respectively) and IR spectra of **3e**, respectively.

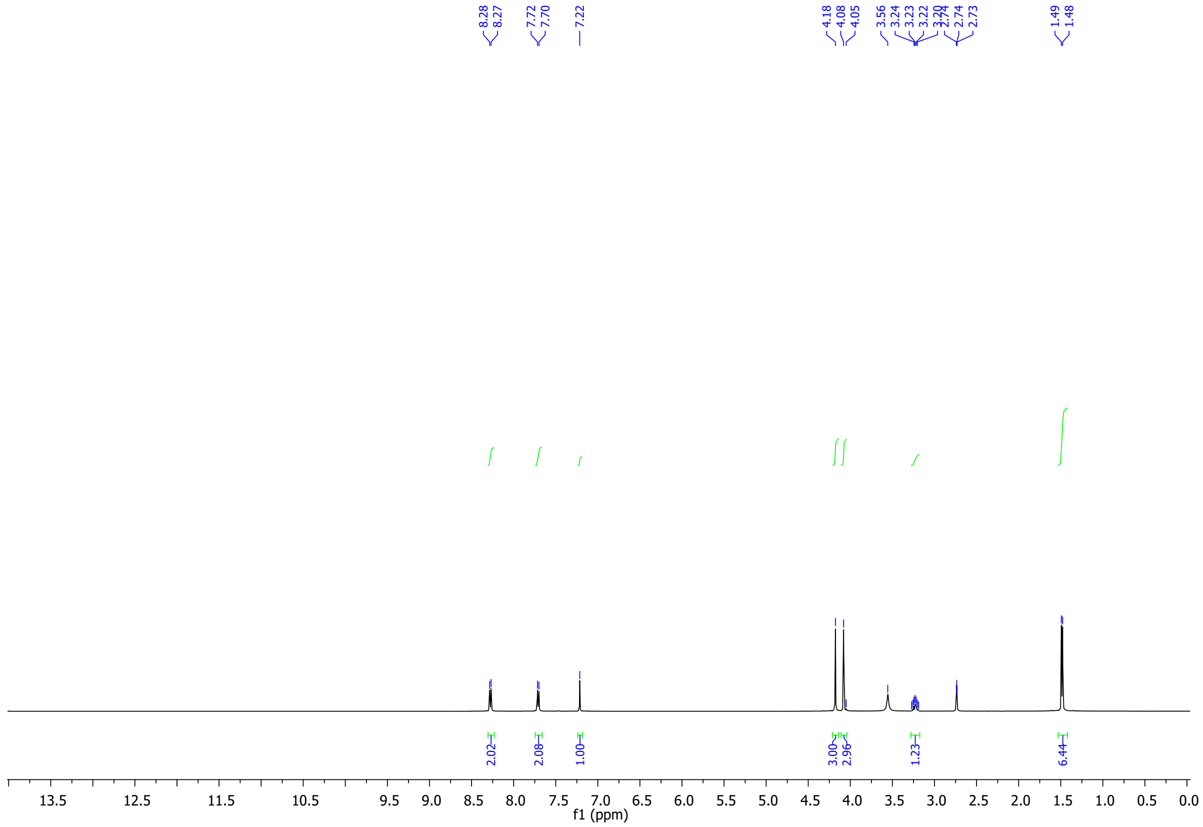
8.28
8.27
7.72
7.70
7.22
4.18
4.08
4.05
3.56
3.24
3.23
3.22
3.20
2.74
2.74
2.73
1.49
1.48
2.02
2.08
1.00
3.00
2.96
1.23
6.44
13.5 12.5 11.5 10.5 9.5 9.0 8.5 8.0 7.5 7.0 6.5 6.0 5.5 5.0 4.5 4.0 3.5 3.0 2.5 2.0 1.5 1.0 0.5 0.0
f1 (ppm)

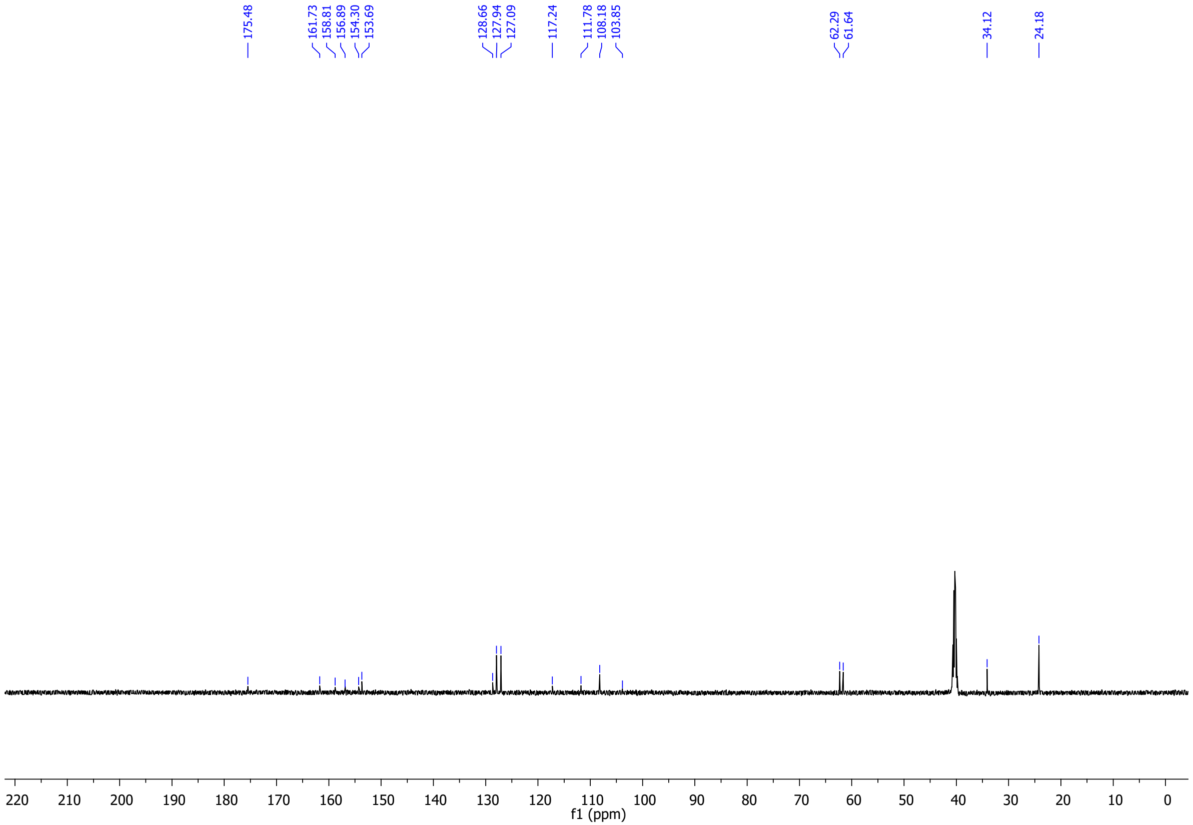
175.48
161.73
158.81
156.89
154.30
153.69
128.66
127.94
127.09
117.24
111.78
108.18
103.85
62.29
61.64
34.12
24.18
220 210 200 190 180 170 160 150 140 130 120 110 100 90 80 70 60 50 40 30 20 10 0
f1 (ppm)

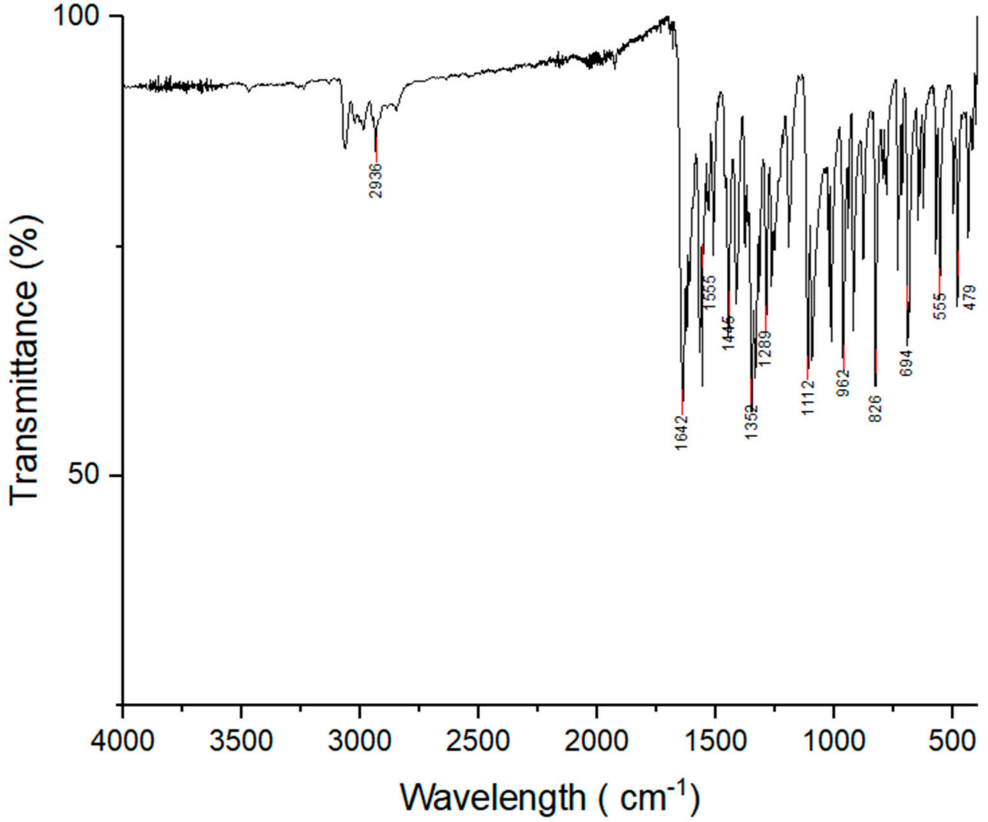

Figure S1.12. NMR (^{1}H- and ^{13}C in DMSO-d_6 at 500 and 125 MHz, respectively) and IR spectra of **3f**, respectively.

Table S1. Crystal data collection and structure refinement for **2a**.

	**2a**
**CCDC**	**2374158**
Empirical formula	$C_{17}H_{14}Br_2O_4$
Formula weight	442.10
Crystal system	Monoclinic
Space group	*P*21/n
a, b, c (Å)	9.1894(18), 15.651(3), 11.286(2)
β (°)	91.418(8)
Volume (Å^3)	1622.8(5)
Z	4
Density (calc) *g/cm*^{3}	1.810
μ (mm^{-1})	5.012
F(000)	872
Crystal size (mm^3)	0.279 x 0.219 x 0.218
$\theta_{min}/\theta_{max}$ (°)	2.824/25.486
Index ranges	-11 ≤ h ≤ 31, -18 ≤ k≤ 18, -13 ≤ -1 ≤ 13
Reflections collected	65435
Independent reflections	3009 [*R*(int) = 0.1213]
Data/restraints/parameters	3009 / 0 / 214
Goodness-of-fit on F^2	1.074
Final *R* indexes [$I \geq 2\sigma (I)$]	R_1 = 0.0519, wR_2 = 0.1154
Final R indexes [all data]	R_1 = 0.0663, wR_2 = 0.1236
Largest diff. peak/hole (*e.Å*$^{-3}$)	0.614 and -0.883

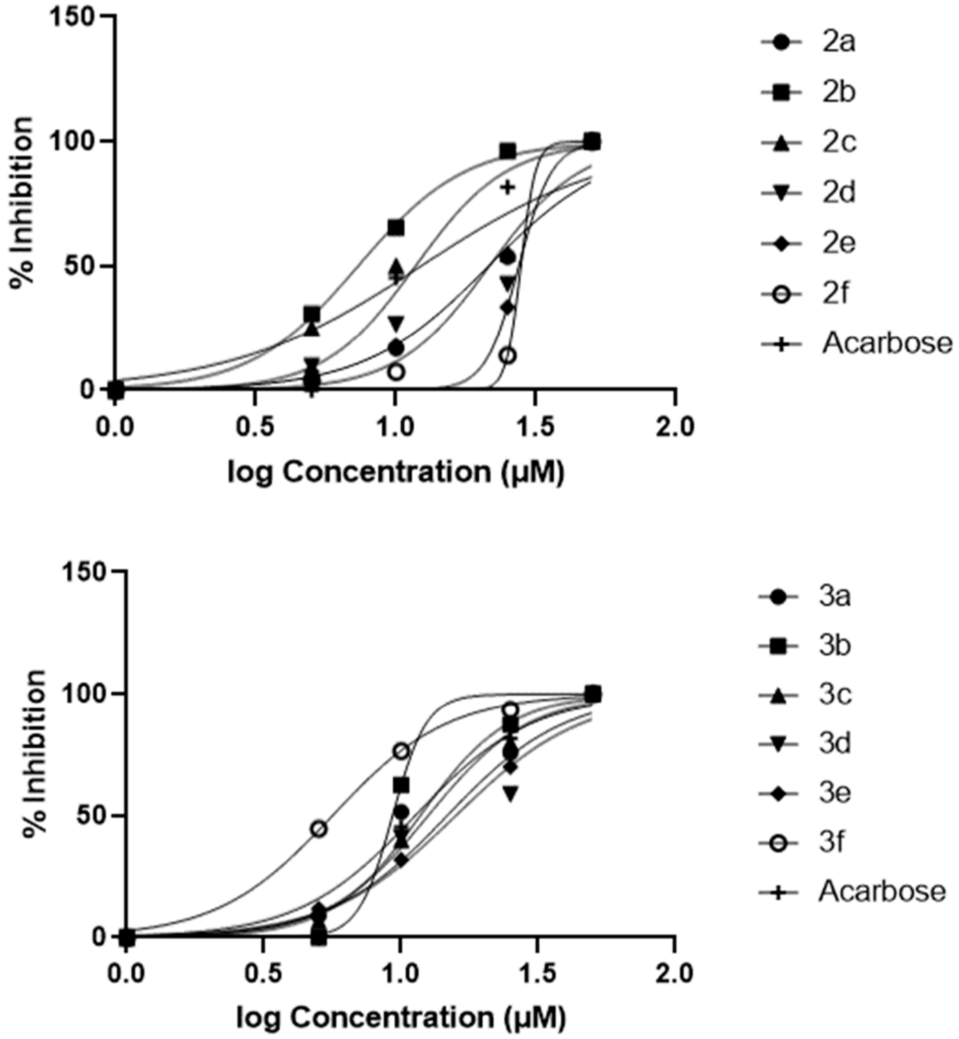

Figure S2: Curves use to calculate the IC_{50} values for series **2a–f** (a), **3a–f** (b) and acarbose against α-glucosidase.

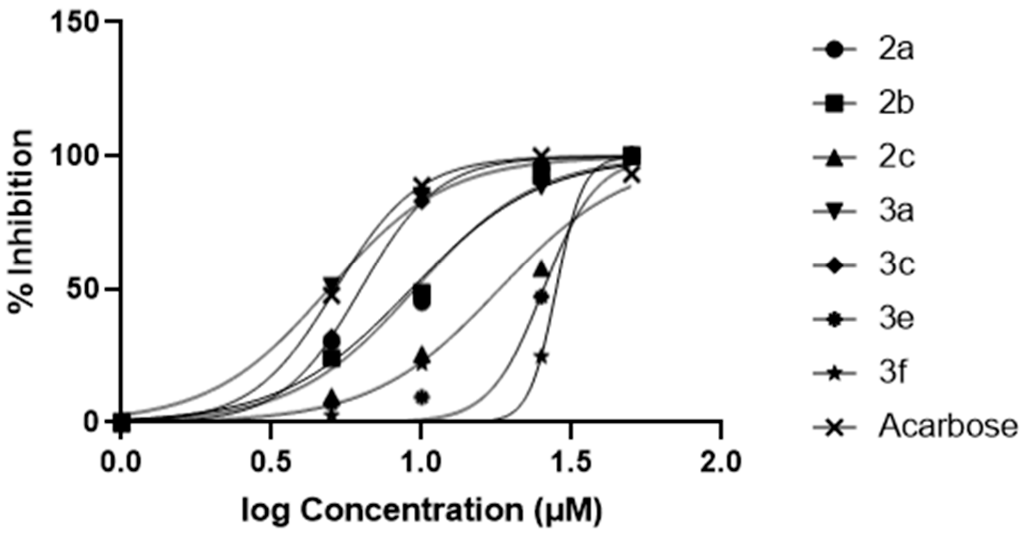

Figure S3: Curves use to calculate the IC_{50} values for series **2a–c**, **3a**, **3c**, **3e**, **3f** and acarbose against α-amylase.

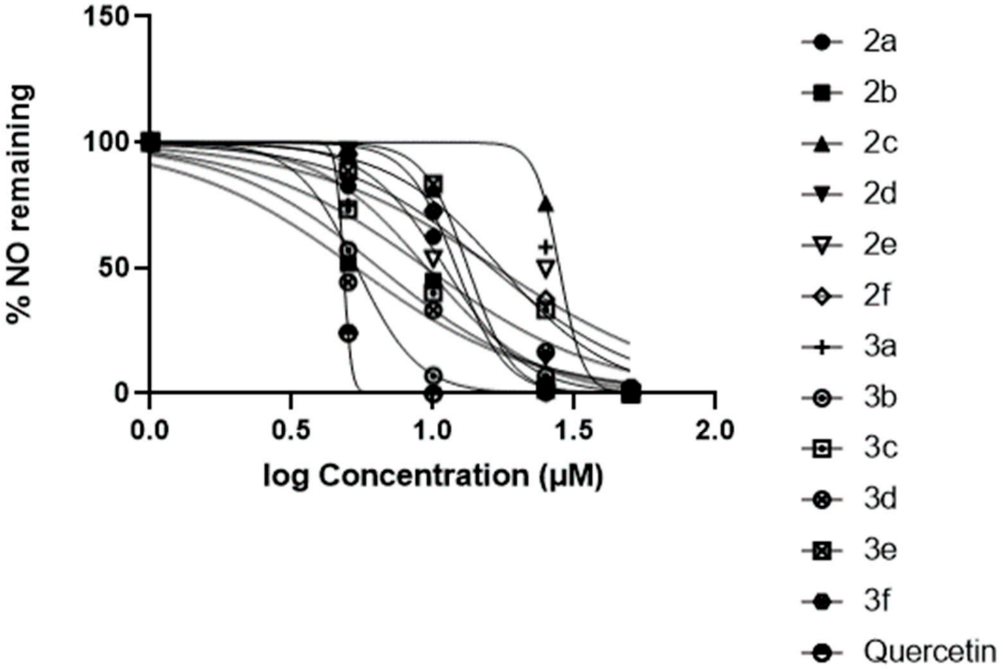

Figure S4: Curves use to calculate the IC_{50} values for series **2a–f**, **3a-f** and quercetin against for nitric oxide (NO) scavenging activity.

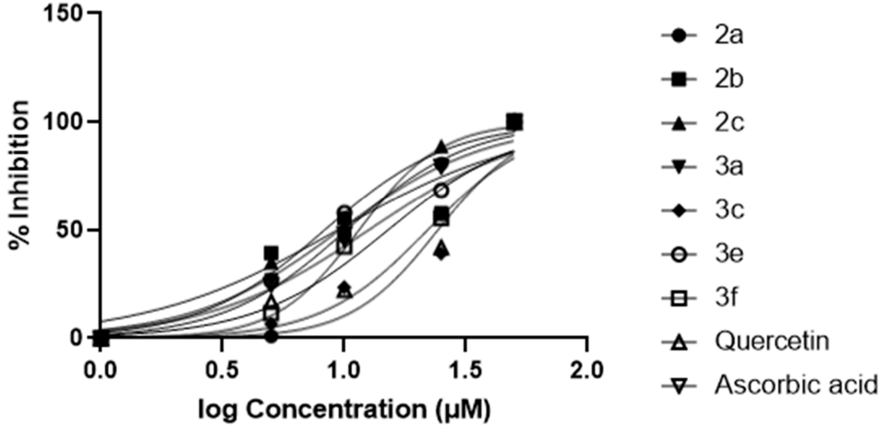

Figure S5: Curves use to calculate the IC_{50} values for series **2a–c**, **3a**, **3c**, **3e**, **3f**, quercetin and ascorbic acid against superoxide dismutase (SOD).

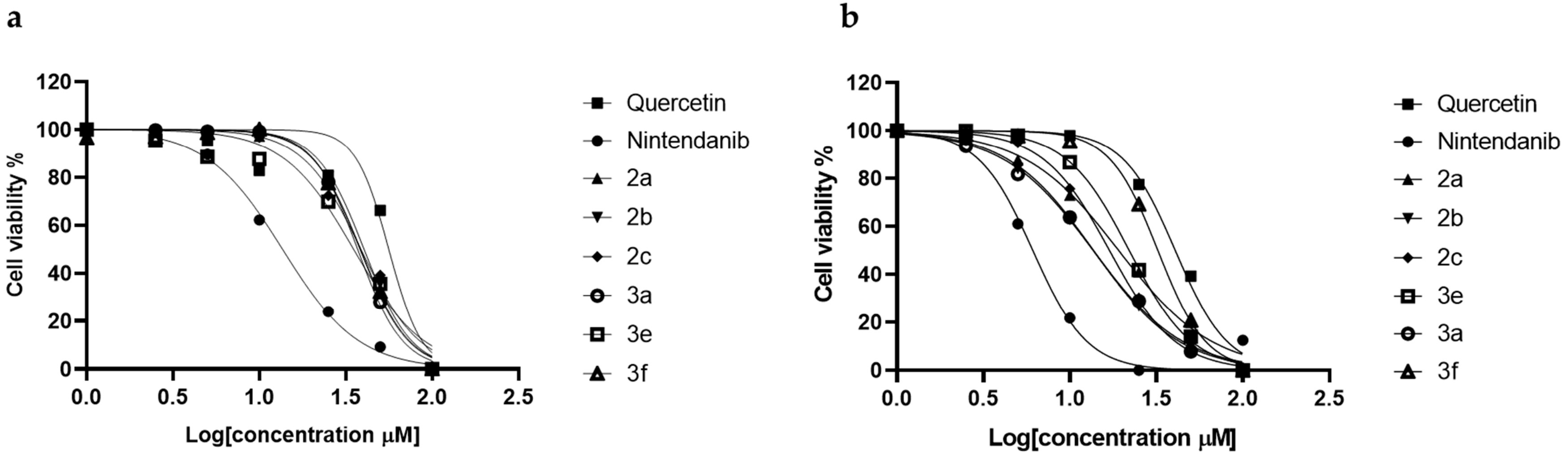

Figure S6: Curves used to calculate the IC_{50} values against the MCF-7 (a) and A549 (B) cell lines

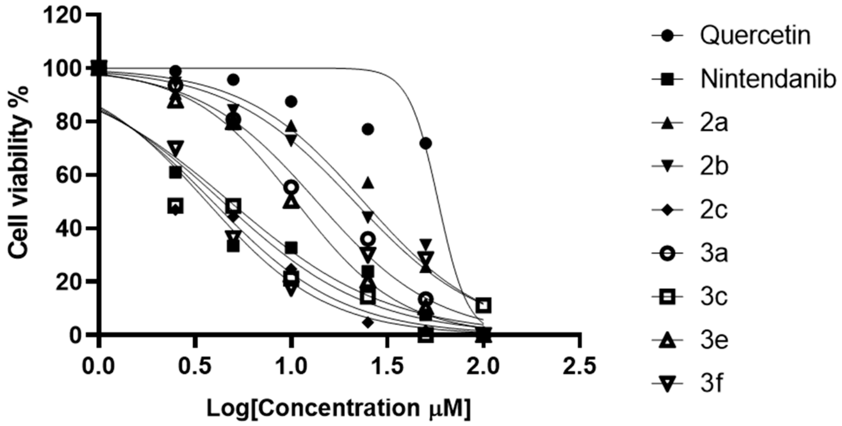

Figure S7: Curves used to calculate the IC_{50} values against the Vero cell line

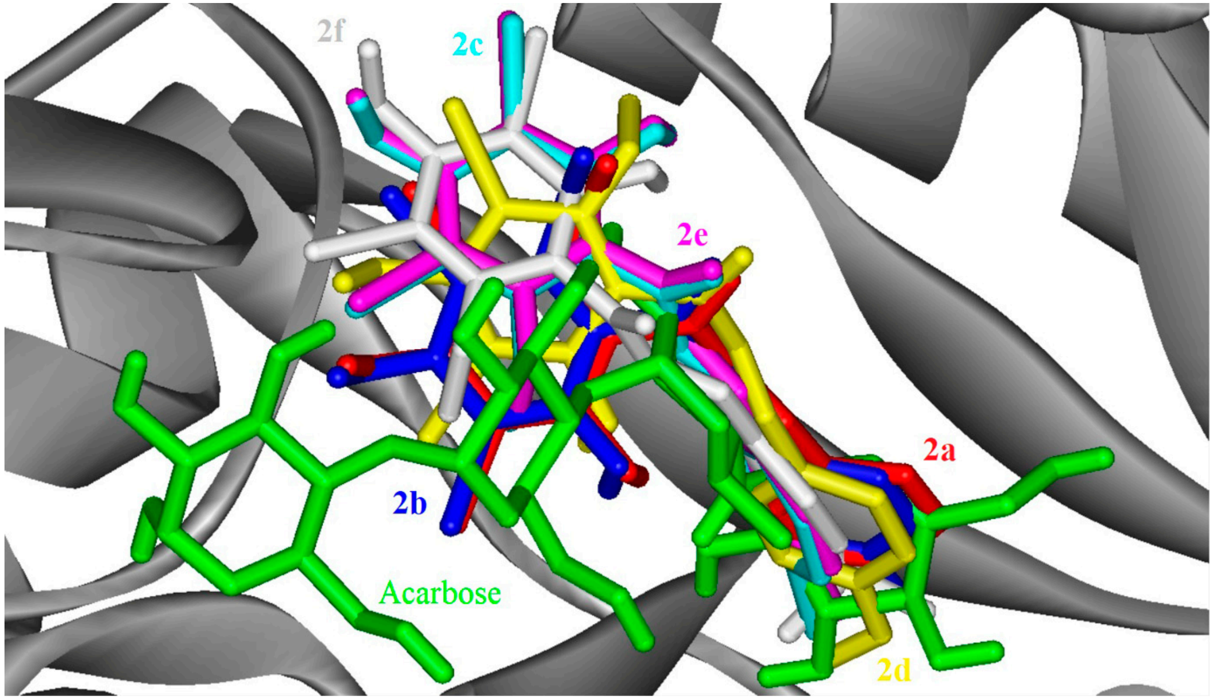

(a)

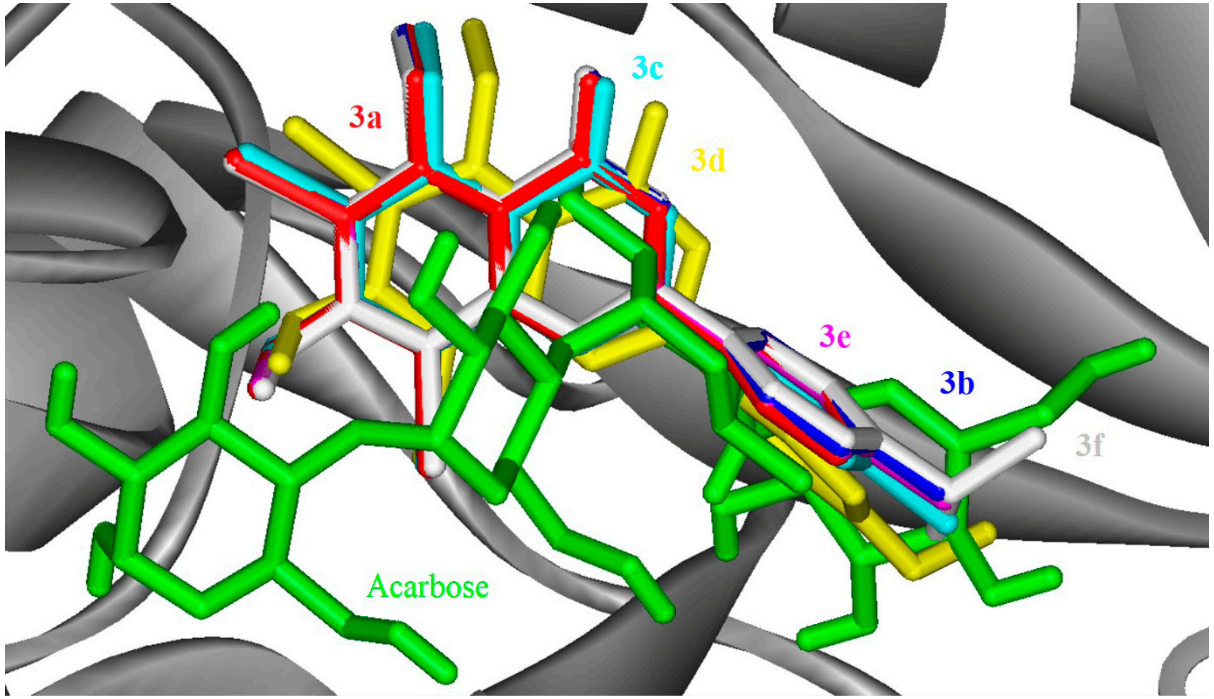

(b)

Figure S8. The interactions of compounds **2** (a) and **3** (b) with the human lysosomal acid-α-glucosidase (PDB id: 5NN8).

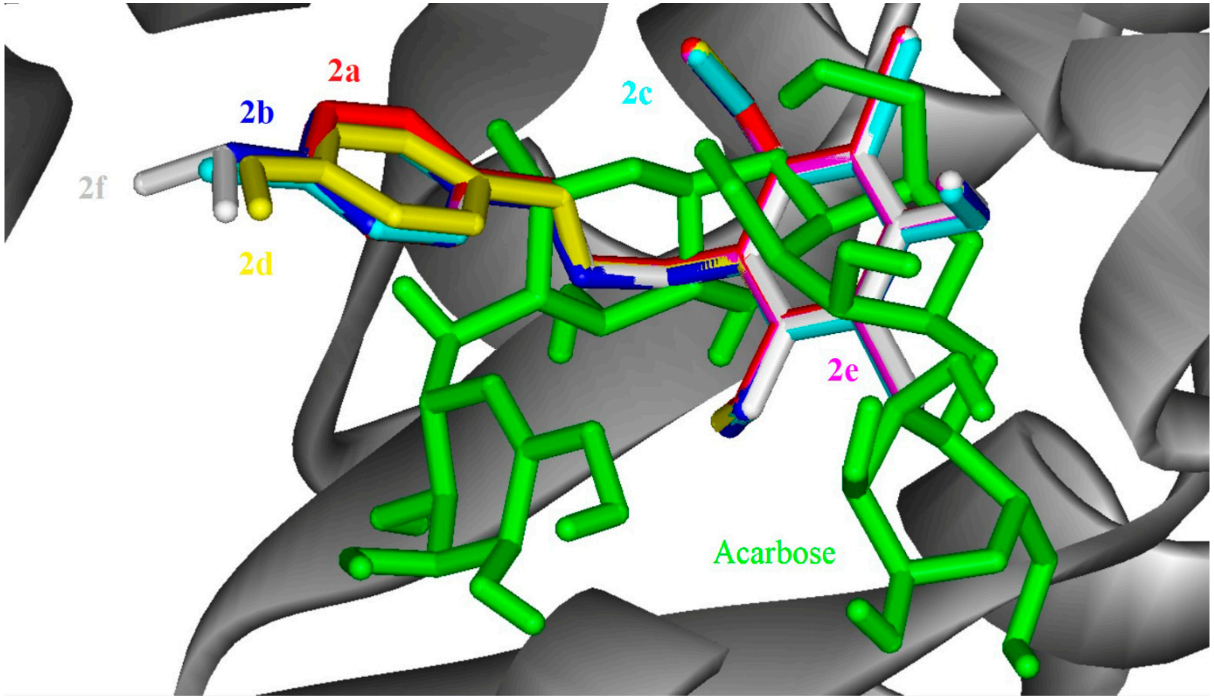

(a).

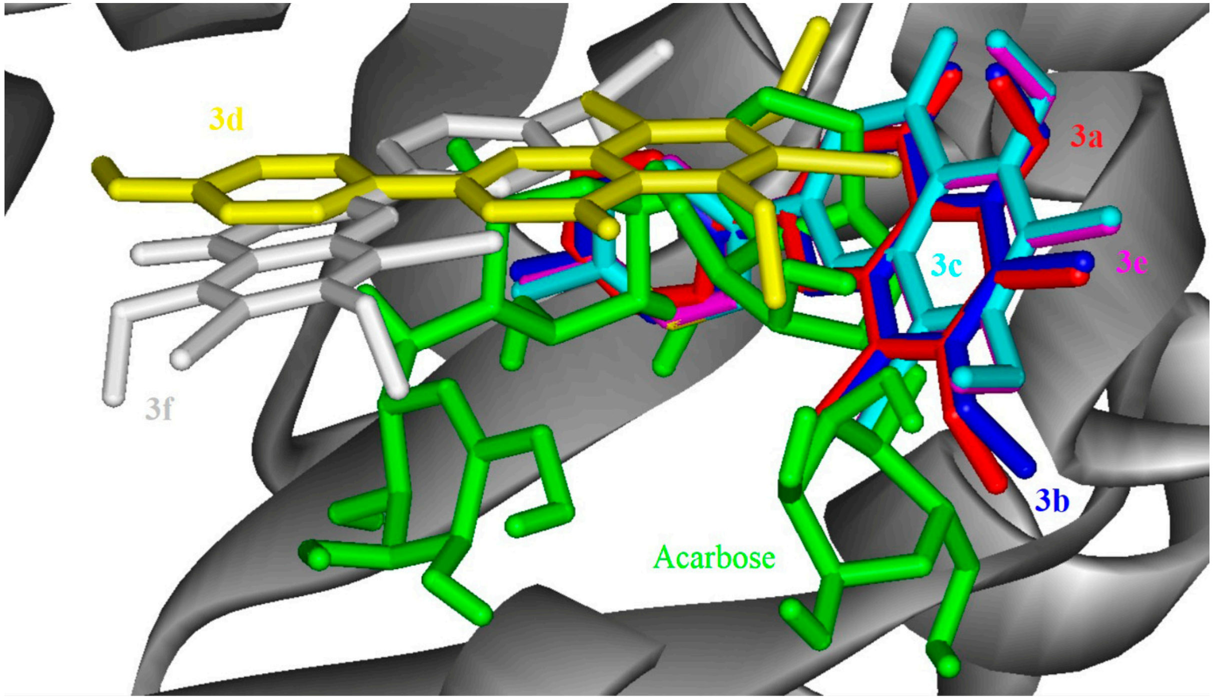

(b).

Figure S9. The interactions of compounds **2** (a) and **3** (b) with the pancreatic α-amylase (PDB: 5E0F).

Table S2. Estimated binding free energies of **2a–f** and **3a–f** against α-glucosidase (PDB id 5NN8) and α-amylase (PDB id 5E0F) from docking simulation.

**Compound**	**Binding free energy (kcal/mol)**	
	α-glucosidase	α-amylase
**2a**	-5.98	-6.61
**2b**	-5.87	-6.69
**2c**	-6.50	-7.26
**2d**	-6.45	-7.22
**2e**	-6.23	-7.04
**2f**	-6.83	-7.92
**3a**	-6.23	-6.87
**3b**	-6.13	-6.78
**3c**	-6.87	-7.04
**3d**	-6.71	-6.35
**3e**	-6.70	-7.05
**3f**	-7.73	-6.77

Table S3: The toxicity prediction of compounds **2** and **3** using ProTox 3.0

Compound	Predicted LD_{50} (mg/kg)	Predicted Toxicity Class (GHS)
**2a**	3000	5
**2b**	3000	5
**2c**	3000	5
**2d**	3000	5
**2e**	3000	5
**2f**	3000	5
**3a**	2570	5
**3b**	2570	5
**3c**	2570	5
**3d**	2570	5
**3e**	2570	5
**3f**	2570	5
